# A plug-and-play system for polycyclic tetramate macrolactam production and functionalization

**DOI:** 10.1186/s12934-024-02630-8

**Published:** 2025-01-10

**Authors:** Anna Glöckle, Sebastian Schuler, Manuel Einsiedler, Tobias A. M. Gulder

**Affiliations:** 1 Chair of Technical Biochemistry, Technische Universität Dresden, Bergstraße 66, 01069 Dresden, Germany; 2 Department of Natural Product Biotechnology, Helmholtz Institute for Pharmaceutical Research Saarland (HIPS), Helmholtz Centre for Infection Research (HZI) and Department of Pharmacy at Saarland University, PharmaScienceHub (PSH), Campus E8.1, 66123 Saarbrücken, Germany

**Keywords:** Polycyclic tetramate macrolactam, Combinatorial biosynthesis, Natural products, Promoter engineering, Heterologous expression

## Abstract

**Background:**

The biosynthesis of the natural product family of the polycyclic tetramate macrolactams (PoTeMs) employs an uncommon iterative polyketide synthase/non-ribosomal peptide synthetase (iPKS/NRPS). This machinery produces a universal PoTeM biosynthetic precursor that contains a tetramic acid moiety connected to two unsaturated polyene side chains. The enormous structural and hence functional diversity of PoTeMs is enabled by pathway-specific tailoring enzymes, particularly cyclization-catalyzing oxidases that process the polyene chains to form distinct ring systems, and further modifying enzymes.

**Results:**

Ikarugamycin is the first discovered PoTeM and is formed by the three enzymes IkaABC. Utilizing the iPKS/NRPS IkaA, we established a genetic plug-and-play system by screening eight different strong promoters downstream of *ikaA* to facilitate high-level heterologous expression of PoTeMs in different *Streptomyces* host systems. Furthermore, we applied the system on three different PoTeM modifying genes (*ptmD*, *ikaD*, and *cftA*), showing the general utility of this approach to study PoTeM post-PKS/NRPS processing of diverse tailoring enzymes.

**Conclusion:**

By employing our plug-and-play system for PoTeMs, we reconstructed the ikarugamycin biosynthesis and generated five derivatives of ikarugamycin. This platform will generally facilitate the investigation of new PoTeM biosynthetic cyclization and tailoring reactions in the future.

**Supplementary Information:**

The online version contains supplementary material available at 10.1186/s12934-024-02630-8.

## Background

**Fig. 1:**
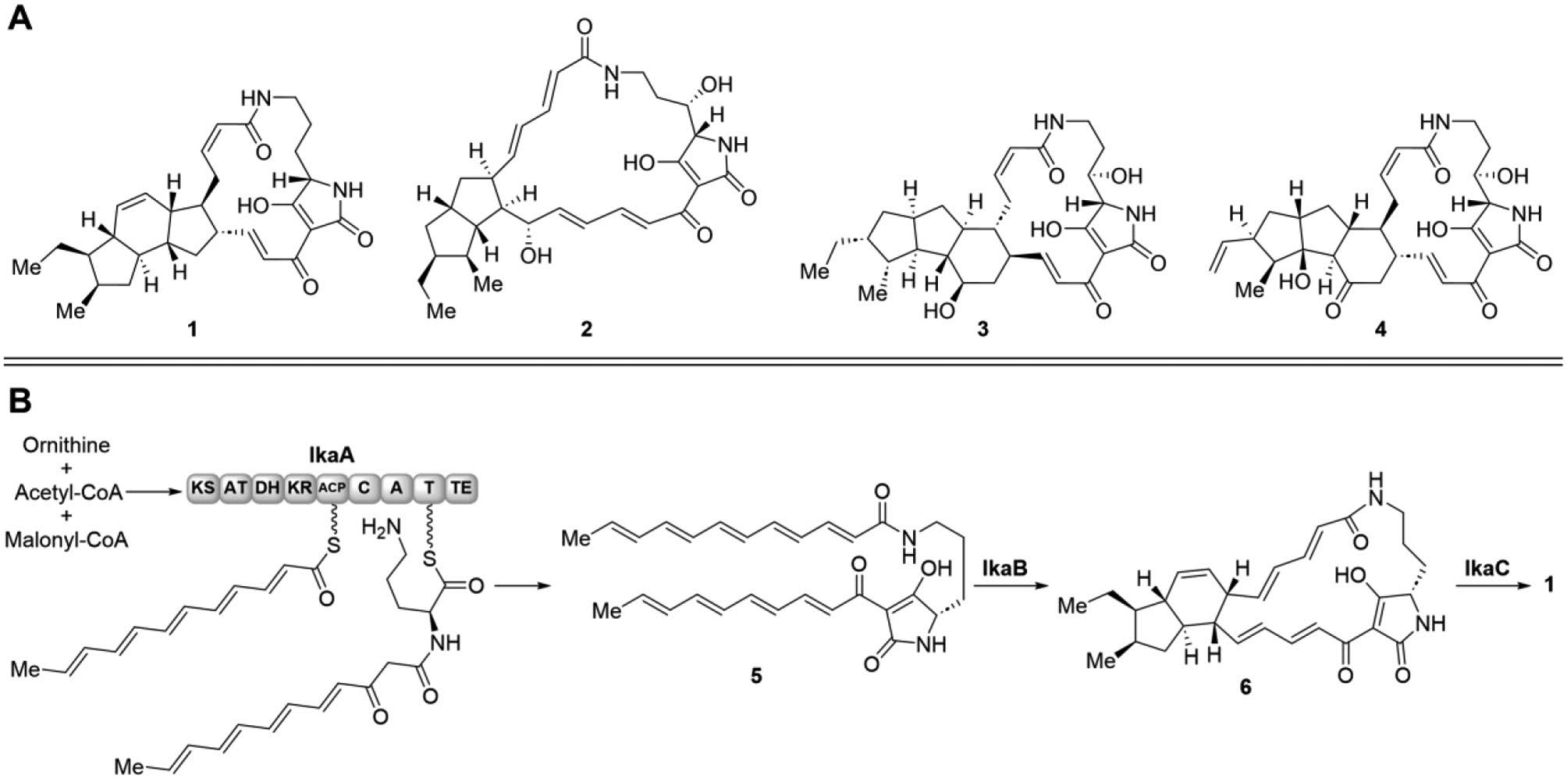
Examples of PoTeMs and their biosynthesis. (**A**) Prominent PoTeM examples ikarugamycin (**1**), alteramide A (**2**), HSAF (**3**), and frontalamide A (**4**). (**B**) Biosynthesis of **1**: the common intermediate lysobacterene A (**5**) derived from the iPKS/NPRS IkaA is cyclized in a stepwise manner to bicyclic **6** by IkaB and to the final 5-6-5-cyclization pattern in **1** by IkaC

Polycyclic tetramate macrolactams (PoTeMs) constitute a growing class of complex and structurally diverse natural products mostly isolated from diverse microorganisms [1]. Depending on their individual structures, PoTeMs exhibit a great number of different pharmacological effects such as antibiotic, antifungal, and cytotoxic properties. Some important representatives are ikarugamycin (**1**) from *Streptomyces phaeochromogenes* var. *ikaruganensis* [2], alteramide A (**2**) from *Alteromonas* sp. [3], HSAF (heat-stable antifungal factor, **3**) from *Lysobacter enzymogenes* strain C3 [4, 5], and frontalamide A (**4**) from *Streptomyces* sp. SPB78 (Fig. 1A) [6]. 

The general organization of PoTeM biosynthetic gene clusters (BGCs) is highly conserved. The central part is a hybrid iPKS/NRPS that generates lysobacterene A (**5**), the common tetramic acid precursor of all PoTeMs [1]. Downstream of this core biosynthetic machinery, flavin-dependent phytoene dehydrogenase homologs (PhyDHs) and nicotinamide-cofactor-dependent alcohol dehydrogenase (ADH) homologs are located [1]. These catalyze formation of the polycyclic ring system typical to PoTeMs. For example, in the biosynthesis of **1**, the PhyDH IkaB catalyzes formation of the two outer rings (one of which putatively formed by a Diels–Alder reaction), while the ADH homolog IkaC installs the inner ring (Fig. 1B). In addition, several PoTeM BGCs encode further modifying enzymes, such as cytochrome P450 enzymes (e.g., *ikaD*, *cftA*, and *ftdF*) [7] and/or PoTeM hydroxylases (e.g., *SD*, *ftdA*) [8] for the (oxidative) decoration of the PoTeM core structure.

With the growing knowledge on PoTeM biosynthesis [1, 5–7, 9–24], targeted approaches to directly identify new PoTeMs were established. Early approaches employed degenerate primers targeting the iPKS/NRPS system to screen unsequenced bacterial strains for the presence of PoTeM BGCs, which led, i.a., to the identification of the BGC encoding **1** [12] as well as to the discovery of the clifednamides and their pathway [25]. Most recent work applied genome mining on sequenced genomes, for example leading to the discovery of pseudoamides A−C from *Pseudoalteromonas elyakovii* ATCC 700519 [20], sahamides A−F from *Saccharopolyspora hirsuta* DSM 44795 [21], koyanamide A from *S. koyangensis* SCSIO 5802 [26], clifednamides D−J from *Kitasatospora* sp. S023 [27], somamycins A−D from *S. somaliensis* SCSIO ZH66 [28], combamides A−D from *Streptomyces* sp. S10 [29], pactamides A−F from *S. pactum* SCSIO 02999 [17], compound D from *S. griseus* IFO 13350 [30], and others [18, 31]. These approaches applied, i.a., promoter engineering and heterologous expression to activate silent BGCs.

The increasing number of sequenced genomes and their analysis with genome mining tools such as antiSMASH [32] reveals an unexpectedly high abundance of PoTeM BGCs in both, Gram-positive and Gram-negative bacteria [1]. Since many of these pathways are still unstudied and remain silent during cultivation of the natural producer under standard laboratory expression conditions, we aimed at developing a plug-and-play platform for heterologous expression to facilitate the production of PoTeMs. The main goals were to generate a platform that (1) is applicable to all PoTeMs, (2) enables the production of PoTeMs in sufficiently high yields, (3) enables a fast and effective cloning, and (4) reliably expresses all genes of interest, particularly focusing on PoTeM tailoring genes.

## Methods

### Reagents, primers, and bacterial strains

The listed commercial materials were purchased from the following manufacturers: LB medium, TB medium, CASO bouillon, soluble starch, nalidixic acid (NA), kanamycin sulfate (kan), chloramphenicol (cam), dipotassium phosphate (K_2_HPO_4_), tryptone, yeast extract, mannitol, and agar from Carl Roth (Karlsruhe, Germany); SERVA DNA stain Clear G from Serva (Heidelberg, Germany); dry magnesium sulfate (MgSO_4_), sodium chloride (NaCl), ammonium sulfate ((NH_4_)_2_SO_4_), calcium carbonate (CaCO_3_), iron sulfate (FeSO_4_ × 7 H_2_O), manganese chloride (MnCl_2_ × 4 H_2_O), zinc sulfate (ZnSO_4_ × 7 H_2_O) from Grüssing (Filsum, Germany); apramycin sulfate (apra) from Glentham Life Sciences (Wiltshire, UK); PeqGOLD Plasmid Miniprep Kit I (C-Line), and chemical solvents (methanol, ethyl acetate, acetonitrile) from VWR (Darmstadt, Germany); deoxynucleotides (dNTPs), Q5 High-Fidelity DNA polymerase, Antarctic phosphatase, restriction enzymes (if available high fidelity), NEBuilder^®^ HiFi DNA Assembly Cloning Kit, T4 DNA polymerase, and Monarch^®^ DNA Gel Extraction Kit from NEB (Frankfurt am Main, Germany); T4-DNA ligase and PCR Purification Kit from Jena Bioscience (Jena, Germany). All kits and enzymes were used according to the manufacturer’s recommended protocol unless otherwise stated. Primers were purchased from Sigma Aldrich (Darmstadt, Germany) at quality “deprotected and desalted”. All used bacterial strains are listed in Table S7.

### Primer design and PCR

Primers were designed using the NEBuilder assembly tool (www.nebuilder.neb.com) and evaluated using Oligo Calc [33]. Gibson homology arms were composed of at least 18 nt with a calculated melting temperature of ≥ 50 °C. HiFi DNA assemblies were simulated using SnapGene software (www.snapgene.com), thereby excluding multiple binding sites of the primers. Primers used in this study are listed in Table S8.

PCRs for insert generation were performed using Q5 High Fidelity DNA Polymerase (NEB, Germany). Colony-screening-PCR was performed using Taq DNA polymerase (NEB, Germany). Clones were picked and resuspended in 50 µL dH_2_O. 5 µL of cell suspension were directly used as template DNA. Cycling was conducted in a T100 Thermal Cycler (Biorad, Germany) or a LifeECO Thermal Cycler (Biozym, Germany).

### DNA assembly

All DNA assembly methods were conducted in a 20 µL reaction batch containing 0.02 pmol vector DNA and 0.1 pmol insert DNA. For conventional cloning, 1× T4-ligase reaction buffer and 0.12 U T4-ligase were added and incubated at 16 °C over night. Ligation reactions were heat-inactivated at 65 °C for 10 min. For HiFi-DNA assembly 1× HiFi DNA assembly master mix was added and incubated at 50 °C for 1 h. For SLIC, 1× NEBuffer 2.1 and 1 µL T4-polymerase were added on ice, incubated 10 min at room temperature, and stored on ice for 10 min.

### Cloning strategy

Expression plasmids were obtained in four individual cloning steps based on the Direct Pathway Cloning (DiPaC) approach (Figure S2) [34, 35]. In a first step, the iPKS/NRPS gene *ikaA* was added to the expression plasmid pSET152-ermE*. The vector was digested with StuI, and *ikaA* (9.4 kb) was amplified by PCR from pSET152-ermE*::*ikaABC* adding homology arms. The two DNA fragments were assembled using HiFi DNA assembly. The different tested promoters were ordered as synthetic genes from Eurofins Genomics in a pEX-A2 vector (Table S9). The promoters were excised from the plasmids by digestion with StuI and XbaI, dephosphorylated, and subsequently ligated into pSET152-ermE*::*ikaA*, which in turn was linearized with StuI and XbaI, to obtain the basic expression constructs for the new plug-and-play system.

For this study, the two cyclizing genes from the ikarugamycin BGC, *ikaB* and *ikaC*, and three different tailoring genes (*ikaD*, *ptmD*, and *cftA*) were added to the plug-and-play platform. Therefore, *ikaBC* was amplified as one piece (2.9 kb) by PCR simultaneously adding homology arms and assembled with the StuI-digested and dephosphorylated basic expression constructs using HiFi DNA assembly. The three modifying genes were amplified by PCR using either gDNA (*ikaD*) or synthesized genes (*ptmD* and *cftA*) as templates. Genes were introduced by digestion of the expression vectors and inserts with XbaI, dephosphorylation, and ligation to obtain the final expression constructs for this study. *cftA* was amplified with homology arms and integrated using SLIC [36]. All cloning steps were verified by analytical restriction digests and sequencing (cf. ESI, Chap. 8).

### Heterologous expression in *Streptomyces *sp. and extraction of PoTeMs

The expression vectors were individually integrated into *S. albus* DSM 40313, *S. lividans* TK24, and *S. coelicolor* M1154 using intergenetic conjugation with *E. coli* ET12567/pUZ8002. Therefore, expression constructs were transformed into *E. coli* ET12567/pUZ8002 by electroporation. PCR-verified clones were grown in LB medium containing apra, cam, and kan (37 °C, 200 rpm) until an OD_600_ of 0.4–0.6 was reached. Cells were washed twice with 10 mL ice-cold LB without antibiotic (4000 rpm, 5 min) and resuspended in 500 µL LB without antibiotic. Spores were resuspended in 500 µL 2×YT (16 g/L tryptone, 10 g/L yeast extract, and 5 g/L NaCl, pH 7.0) and heat-activated at 50 °C for 10 min. Cells and spores were combined, harvested (4000 rpm, 2 min), plated on MS agar (20 g/L mannitol, 20 g/L soya flour, 20 g/L agar) supplemented with 10 mm MgCl_2_ and 60 mm CaCl_2_, and cultivated at 30 °C. After 16–20 h, conjugation plates were overlaid with 1 mg NA and 1 mg apra. Colonies were obtained after 4–7 days (30 °C) and verified by colony-screening-PCR.

*Streptomyces* sp. were first grown in CASO supplemented with apra and NA for 3–5 days (28 °C, 200 rpm). After upscaling to a 20 mL CASO culture, 5 mL of a dense culture were used to inoculate 50 mL ISP-4 (10 g/L soluble starch, 1 g/L K_2_PO_4_, 1 g/L MgSO_4_, 1 g/L NaCl, 2 g/L (NH_4_)_2_SO_4_, 2 g/L CaCO_3_, 1 mg/L FeSO_4_ × 7 H_2_O, 1 mg/L MnCl_2_ × 4 H_2_O, 1 mg/L ZnSO_4_ × 7 H_2_O) without antibiotics. The main culture was harvested (6000 rpm, 10 min) after 7 days incubation (28 °C and 200 rpm). Pellet and supernatant were extracted individually. The pH of the supernatant was set to 5 using 1 m HCl_aq_ and the samples were extracted three times with ethyl acetate. The combined organic phases were dried over MgSO_4_, filtered, and the solvent was removed in vacuo. The cells were resuspended in 20 mL methanol/acetone (1:1), sonicated for 30 min in a sonicating bath, and centrifuged (10 min, 6000 rpm). The supernatant was dried in vacuo.

### HPLC-MS analysis

Organic extracts were analyzed using a computer-controlled Jasco HPLC System composed of a MD-2010 Plus Multiwavelength Detector, a DG-2080-53 3-Line Degasser, two PU-2086 Plus Intelligent Pumps, an AS-2055 Plus Intelligent Sampler, a MIKA 1000 dynamic mixing chamber, a 100 µL sample loop (Portmann Instruments AG Biel-Benken), and a LCNetII/ADC. For LC-MS measurements, this system was coupled to an exPressIon^®^ MS instrument (Advion) containing a single-quadrupole mass analyzer used in combination with a N118LA nitrogen generator (Peak Scientific) and a RV12 high vacuum pump (Edwards). For chromatographic separation, a Eurosphere C8 column with precolumn (Knauer 10XE084E2J, 100 × 3 mm, 100-5 C8 A) was utilized. H_2_O (A) and acetonitrile (B), both supplemented with 0.05% TFA, with a flow rate of 1 mL/min were used as solvents. The gradient was as follows: 0–2 min 100% A, 2–10 min 100–55% A, 10–20 min 55% A, 20–24 min 0% A, 24–28 min 100% A.

For every construct, the extracts of the medium and the cells were analyzed individually. The relative quantities of the PoTeMs were determined by integrating the corresponding peak. The final analysis used the average values from the biological triplicates (extract of the medium and the cells combined). Error bars represent the standard deviation.

### HR-MS

For high-resolution mass spectrometry and MS/MS measurements after HPLC separation, a Bruker UHPLC consisting of an Elute autosampler and an HPG 1300 pump was used. This was coupled to an Impact II mass spectrometer with an ESI source and Q-TOF mass analyzer manufactured by Bruker. The following parameters were used: solvents: A = H_2_O + 0.05% formic acid (FA), B = ACN + 0.05% FA; separation method: 0–2 min: 95% A, 2–25 min: 95–5% A, 25–28 min: 5% A, 28–30 min: 95% A; flow rate: 0.3 mL/min; column: Intensity Solo 2 C18, 100 × 2.1 mm (in column oven: 40 °C). For MS/MS, auto-MS/MS mode with 20–50 eV collision energy (N_2_) was used. The system was controlled by Bruker Compass^®^ HyStar software; analysis was conducted with Bruker Compass^®^ Data Analysis software.

### Compound purification

The PoTeMs were purified from raw extracts by semi-preparative HPLC, either using a system made by Jasco (composed of a UV-1575 Intelligent UV/VIS Detector, two PU-2086 Intelligent Pumps, a MIKA 1000 dynamic mixing chamber, a 5000 µL sample loop (Portman Instruments AG Biel-Benken), a LC-NetII/ADC, and a Rheodyne injection valve; Galaxie software) or from Knauer (consisting of a degasser, a P 6.1 L pump, a 1000 µL injection port, and a Smartline UV detector 2500; ClarityChrom) with detection at 220 nm. H_2_O (A) and acetonitrile (B), both supplemented with 0.05% TFA, were used as solvents. Individual separation conditions were: Capsimycin G (**8**, 10.5 min): column: Eurospher II, 100-5 C18A, 250 × 16 mm; gradient: 0–2 min: 75% A, 2–22 min: 75–25% A, 22–32 min: 0% A; flow rate 5 mL/min. Butremycin (**9**, 21,5 min): column: Eurospher II, 100-5 C8A, 250 × 8 mm; gradient: 0–2 min: 95% A, 2–22 min: 95–0% A, 22–26 min: 0% A, 26–30 min: 95% A; flow rate 3 mL/min. Clifednamides A (**10**, 35.6 min) and C (**11**, 22.2 min): column Eurospher II 100-5 C18A, 250 × 8 mm; gradient: 0–2 min: 75% A, 2–37 min: 75–40% A, 37–50 min: 0% A; flow rate 5 mL/min.

### NMR

Nuclear Magnetic Resonance (NMR) spectra were recorded on Bruker AVANCE II 300, ASCEND 500, and AVANCE III 600 spectrometers at ambient temperature. The chemical shifts are given in δ-values (ppm) relative to TMS (^1^H, ^13^C). ^1^H and ^13^C spectra were referenced internally using the residual proton resonances of the deuterated solvents (DMSO-*d*_5_: δ_H_ = 2.50 ppm; pyridine-*d*_4_: δ_H_ = 7.22 ppm) and the corresponding carbon shifts (DMSO-*d*_6_: δ_C_ = 39.52 ppm; pyridine-*d*_5_: δ_C_ = 123.87 ppm), respectively. The coupling constants *J* are given in Hertz [Hz] and were determined assuming first-order spin-spin coupling. The following abbreviations were used for the allocation of signal multiplicities: s – singlet, bs – broad singlet, d – doublet, bd – broad doublet, t – triplet, bt – broad triplet, q – quartet, qnt – quintet, sxt – sextet, spt – septet, m – multiplet, or any combination thereof.

## Results

### Establishing a plug-and-play system for PoTeM production

**Fig. 2:**
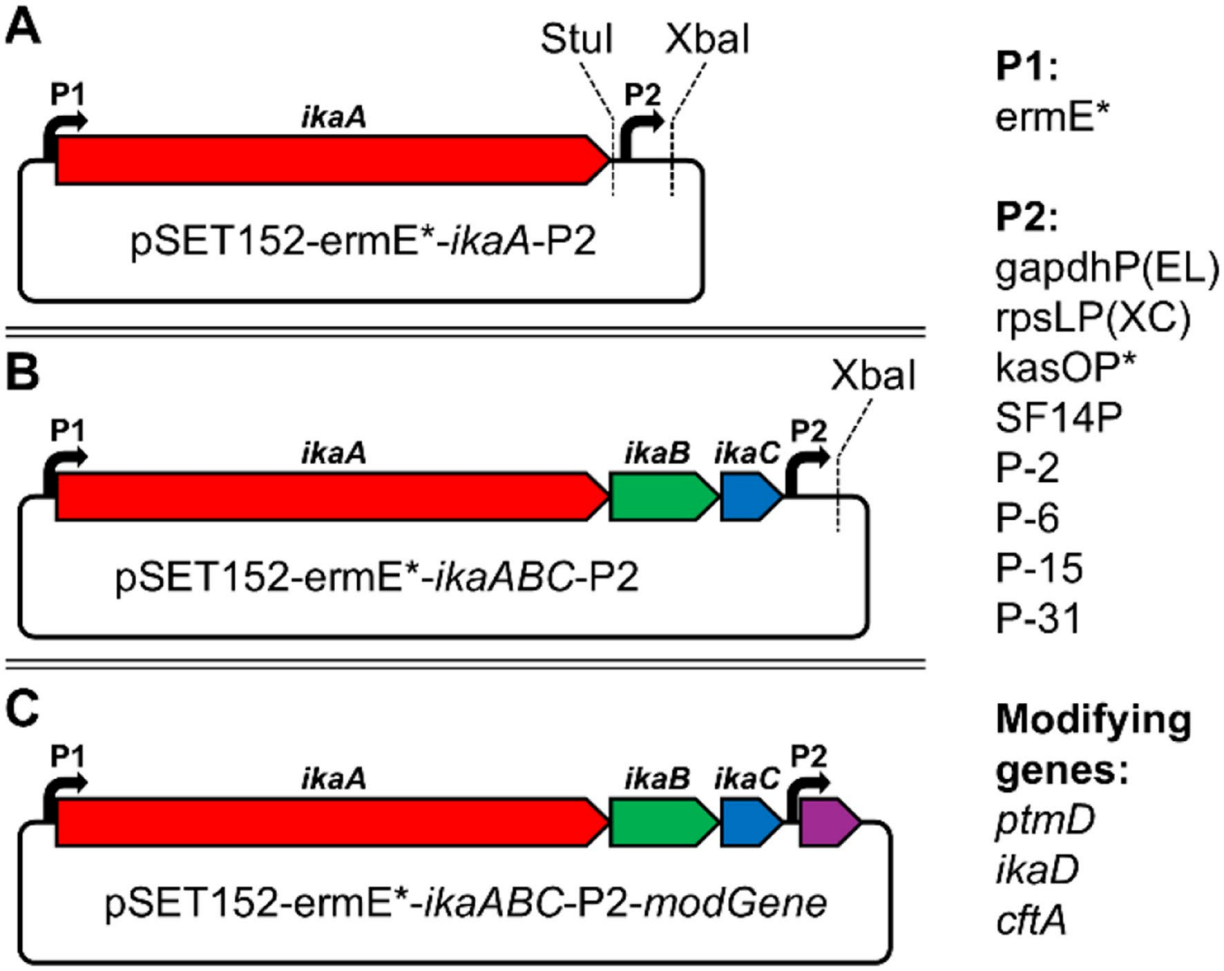
Plug-and-play system for the heterologous expression of PoTeMs. The system is based on a pSET152 vector and designed for *Streptomyces* expression. (**A**) The basic expression vector contains the iPKS/NRPS *ikaA* under control of an ermE* promoter (P1) and a second promoter (P2) downstream of the gene. (**B**) The expression vector contains all essential genes *ikaABC* for the biosynthesis of **1**. The construct can be used to investigate the influence of modifying enzymes on **1**. (**C**) Plug-and-play system containing either *ptmD*, *ikaD*, or *cftA* under the individual control of eight different, strong constitutive promoters (P2). Red: *ikaA*, green: *ikaB*, blue: *ikaC*, and purple tailoring genes *ptmD*/*ikaD*/*cftA*. P1: ermE* and P2: gapdhP(EL), rpsLP(XC), kasOP*, SF14P, P-2, P-6, P-15, or P-31

To establish an efficient recombinant expression platform, we decided to use *Streptomyces* sp. as heterologous host, since the majority of PoTeM BGCs natively derive from this species [1]. As all currently known PoTeM biosynthetic pathways rely on the prototypical iPKS/NRPS system to provide the common precursor molecule **5**, we installed *ikaA* from *Streptomyces* sp. Tü6239 [12, 37] under the control of the strong constitute promoter ermE* [38] (P1) into a pSET152 vector backbone by Direct Pathway Cloning (DiPaC) [35] to form the basis of the plug-and-play platform (Fig. 2A). We selected *ikaA*, as previous studies had shown successful heterologous expression of **1** in *E. coli* [12] and *Streptomyces* [14] using the *ikaABC* gene cassette, proving that the precursor **5** should be produced in sufficient amounts. The resulting construct was tested by expression in *S. albus* DSM 40313 and the production of **5** was confirmed by HPLC-MS (Figure S1). Downstream of *ikaA*, we installed two unique restriction sites (StuI and XbaI) flanking a second promoter (P2). The StuI site can be utilized to integrate genes encoding cyclization enzymes of choice. For establishing our system, we exemplarily used the ikarugamycin tailoring genes *ikaBC*, which were integrated by HiFi DNA assembly [39]. The XbaI site was designed to readily integrate genes involved for late-stage PoTeM functionalization by ligation cloning or SLIC [36]. Since promoter activities are moderately studied in *Streptomyces* [40], we integrated eight different promoters to determine optimal expression conditions for individual genes (Fig. 2). These included gapdhP(EL), which is located upstream of the gapdh operon in *Eggerthella lenta*, and rpsLP(XC), which is located upstream of the operon coding for the 30S ribosomal proteins S12 and S7 and elongation factors in *Xylanimonas cellulosilytica*, since they were reported as most active ones among a set of various promoters derived from house-keeping genes of different organisms [41]. kasOP* was previously generated by targeted and random mutagenesis of a promoter positioned upstream of a PKS in *S. coelicolor* [42]. It showed an upregulated expression of up to five times compared to ermE* [42]. The phage I19 promoter SF14P was isolated from a *Streptomyces* strain and also showed a high expression level [43]. The last four promoters P-2, P-6, P-15, and P-31 were discovered in a large study that compared non-transcribed intergenic regions in *S. albus* J1074 and showed a significant activating effect [44]. 

### Bispecific monooxygenase IkaD

As we focused on enabling investigations on late-stage tailoring enzymes in this study, we integrated *ikaBC* into the StuI site by Gibson assembly (Fig. 2B, Figure S2), thus delivering the ikarugamycin core structure **1** in high yields upon heterologous expression in *Streptomyces* (Figure S1). This construct was further used to probe the influence of different modifying genes acting on PoTeM core structures, exemplarily for **1**, under the control of the eight selected constitutive promoters. As proof-of-concept, *ikaD* was chosen as the first modifying enzyme. This bispecific P450 monooxygenase was initially characterized from the gene cluster of **1** from *S. xiamenensis* 318 [45] and was intensively investigated in vivo and in vitro. It adds an epoxide at the C-13/14 of its native substrate **1**, resulting in capsimycin B (**7**) [7, 45]. Additionally, IkaD hydroxylates **7** at C-29 resulting in capsimycin G (**8**) [7, 45]. The activity of IkaD acting on the 5-5-cyclic pattern of combamides was also elucidated in vivo and in vitro [16, 23]. 

**Fig. 3:**
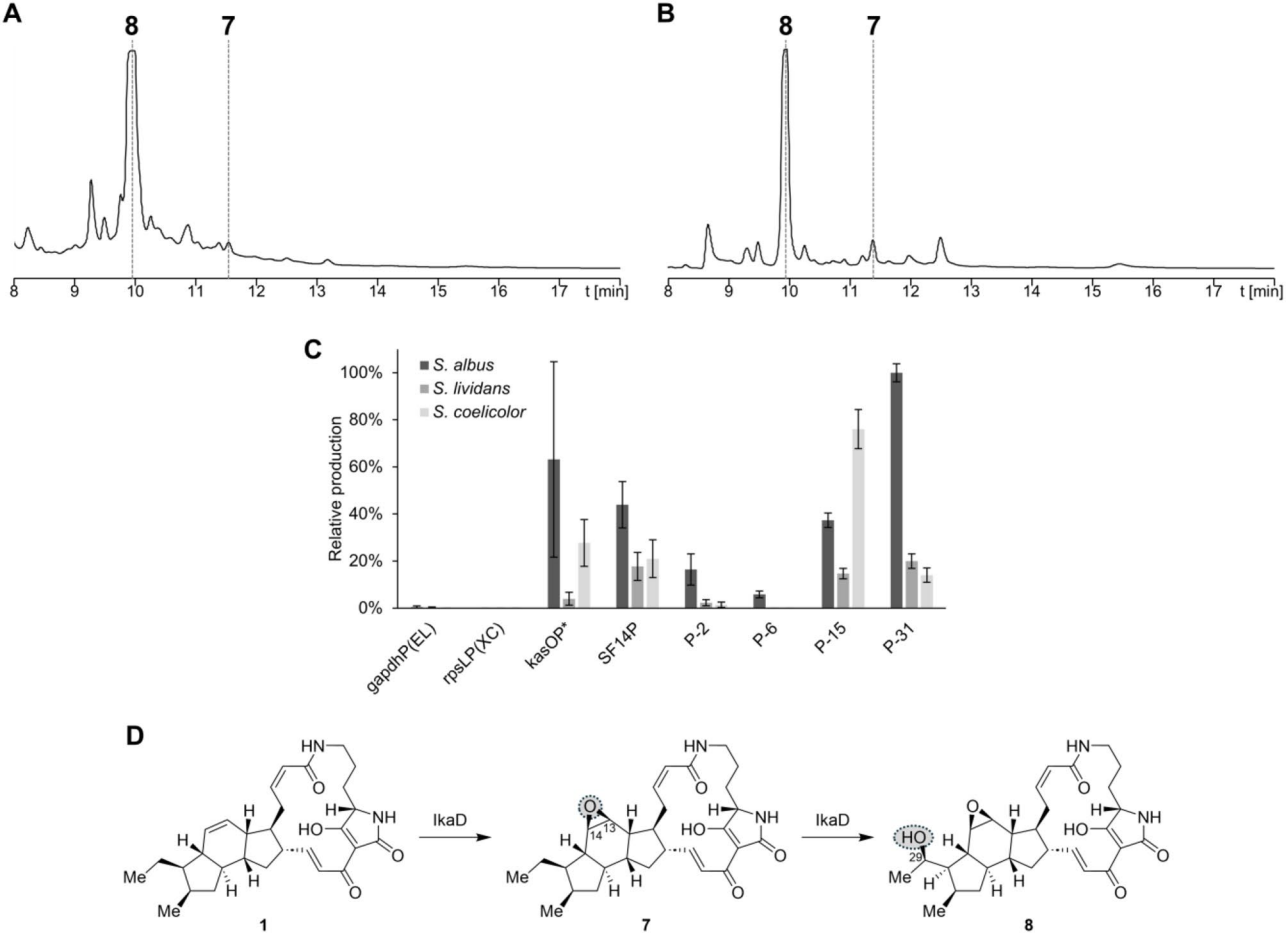
Conversion of **1** to **7**/**8** by IkaD using different promoters and *Streptomyces* expression hosts. HPLC-UV (220 nm) chromatogram of the extracts of (**A**) the medium and (**B**) the cells, exemplarily shown for expression with *S. albus* DSM 40313 equipped with pSET152-ermE*-*ikaABC*-kasOP*-*ikaD*. (**C**) Relative amount of capsimycin G (**8**) produced by IkaC with tailoring gene expression under the control of eight different promoters in three different stains: *S. albus* DSM 40313 (black), *S. lividans* TK24 (dark grey), and *S. coelicolor* M1154 (light grey). The yields of all tested second promoters were calculated relative to P-31, the construct with the highest average conversion rate. (**D**) Stepwise modification of **1** to **7** and **8** by IkaD

*ikaD* was added to the expression platform containing *ikaABC* under the control of the eight different second promoters. The presence of the restriction site (XbaI) enabled a fast insertion by classical ligation cloning. The constructs were expressed in three different *Streptomyces* strains (*S. albus* DSM 40313, *S. lividans* TK24, and *S. coelicolor* M1154) under the conditions resulting in the highest yields of **1** in previous expression experiments (see above). For every promoter, biological triplicates were cultured, extracted, and analyzed by LC-MS for PoTeM quantification. In *S. albus*, **1** was almost completely converted to the two oxidatively modified compounds **7** and **8** (Fig. 3AB). The major product was the double-modified ikarugamycin derivative **8** (*m/z* 511.2835), whereas the yield of the single-modified intermediate **7** (*m/z* 495.2850) was significantly lower. Both compounds were found in the extracts of both, the medium and the cells with higher amounts in the medium (cf. Figure S3). Additionally, trace amounts of further PoTeM decomposition products were detected in the extract of the medium. These compounds were generated under acidic extraction conditions, which led to a nucleophilic epoxide-opening by methanol or water [45]. The yield of **8** significantly depended on the used strain and promoter (Fig. 3C). Generally, the expression worked best in *S. albus* and barely in *S. lividans*. In *S. albus*, the utilization of P-31 as second promoter resulted in the highest average production of **8**, which was exceeded by certain clones using kasOP*. SF14P and P-15 also yielded decent amounts of compound **8**, and promoters P-2 and P-6 enabled production of **8** in low amounts. With the remaining promoters (gapdhP(EL) and rpsLP(XC)), no conversion of **1** was observed, which indicates that these were completely inactive. The expression in *S. coelicolor* resulted in significantly lower yields, but again kasOP* and SF14P belonged to the best-performing promoters. The best promoter in *S. coelicolor* was P-15. In *S. lividans*, PoTeM expression was generally very low. The most active promoters were kasOP* and P-31.

For structural validation, large-scale fermentation was performed with *S. albus* and the well-performing expression construct containing the kasOP* promoter. Compound isolation yielded 2–3 mg/L of compound **8**, and its structure was validated by 1D and 2D NMR (cf. ESI, Table S2, Figures S15–20). Consistent with literature, IkaD introduces two modifications to **1**: an epoxide at the position of the former double bond (C-13/14) and a hydroxy group at C-29 (Fig. 3D) [7, 45]. In addition, based on the HRMS data and the higher retention time, **7** was unambiguously identified as the single-modified product of IkaD.

### PoTeM hydroxylase PtmD

**Fig. 4:**
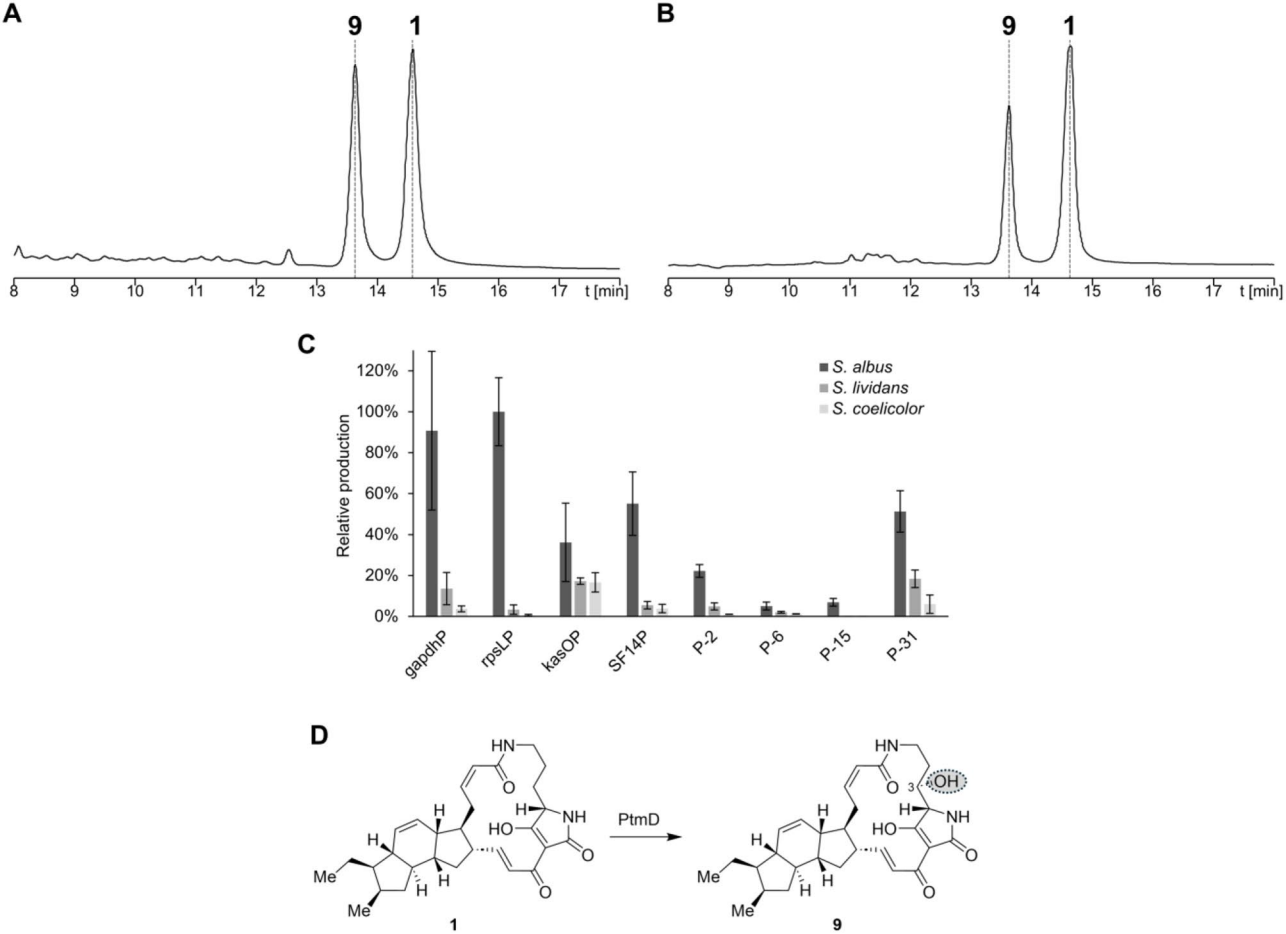
Conversion of **1** to **9** by PtmD using different promoters and *Streptomyces* expression hosts. HPLC-UV (220 nm) chromatogram of the extracts of (**A**) the medium and (**B**) the cells, exemplarily shown for expression with *S. albus* DSM 40313 equipped with pSET152-ermE*-*ikaABC*-gapdhP(EL)-*ptmD*. (**C**) Relative amount of butremycin **9** produced by PtmD, with tailoring gene expression under the control of eight different promoters in three different strains: *S. albus* DSM 40313 (black), *S. lividans* TK24 (dark grey), and *S. coelicolor* M1154 (light grey). The yields of all tested second promoters were calculated relative to rpsLP(XC), the construct with the highest average conversion rate. (**D**) Modification of **1** to **9** by PtmD

We next investigated a modifying enzyme that naturally acts on 5-5-6-cyclic PoTeMs to determine whether it can also be applied for modifications on 5-6-5-cyclic **1**. We selected *ptmD* from the pactamide gene cluster, described as a hydroxylase whose regioselectivity has not been fully characterized [17, 18]. The *ptmD* gene was efficiently inserted downstream of the eight different second promoters using ligation cloning and the expression was again performed in the three different *Streptomyces* strains. The analysis of the extracts revealed that **1** remained the primary product under all tested conditions. However, a new PoTeM (**9**) with a shorter retention time and an *m/z* 495.2850 was also detected, consistent with a hydroxylated derivative of **1** ([M + H]^+^ calculated *m/z* 495.2853; Fig. 4AB). As observed for IkaD above, *S. albus* yielded the highest amounts of the modified PoTeM (Fig. 4C). The conversion of **1** in the two other strains was even lower than during experiments with IkaD. Interestingly, the promoters gapdhP(EL) and rpsLP(XC), which were essentially inactive with IkaD, yielded the highest conversion rates for PtmD. Except for P-6 and P-15, all other promoters also yielded acceptable conversion rates. For structural validation, the fermentation of the *S. albus* strain harboring the construct containing the gapdhP(EL) promoter was scaled up (2 L), and 1.8 mg/L of compound **9** were isolated.

Initially, *ptmD* was presumed to function as a hydroxylase at position C-31 of pactamide A [17]. However, it was later hypothesized to act as a PoTeM hydroxylase, adding a hydroxy group at the C-3 position [18]. This is also supported by its sequence similarity to PoTeM hydroxylases (over 50% amino acid identity) and its location upstream of the iPKS/NRPS, where commonly PoTeM hydroxylases can be found [17]. However, no detailed investigation has been conducted so far. A comparison of 1D NMR data of **9** and **1** revealed differences in the region of the ornithine-derived molecular portion. The signals of the two hydrogens at C-3, which appear in the region between δ_H_ 1.69–1.88 ppm in **1**, were missing. Instead, new signals at 3.80 and 5.09 ppm appeared (cf. ESI. Figure S21). Together with 2D NMR analysis (cf. ESI, Figures S22–24), the hydroxy group was localized at C-3 (Figure S25), and the diastereoselectivity was confirmed by extensive comparison of literature NMR data with those of our isolated substance (cf. Table S4 and Figures S26/S27). Thus, PtmD was confirmed to act as a PoTeM hydroxylase that converts **1** to butremycin (**9**; Fig. 4D) [8]. Furthermore, it was shown that PtmD, which derives from a BGC encoding a 5-5-6-ring PoTeM, can also function on the 5-6-5-carbocyclic system.

### Applying the plug-and-play system for PoTeM modification

**Fig. 5:**
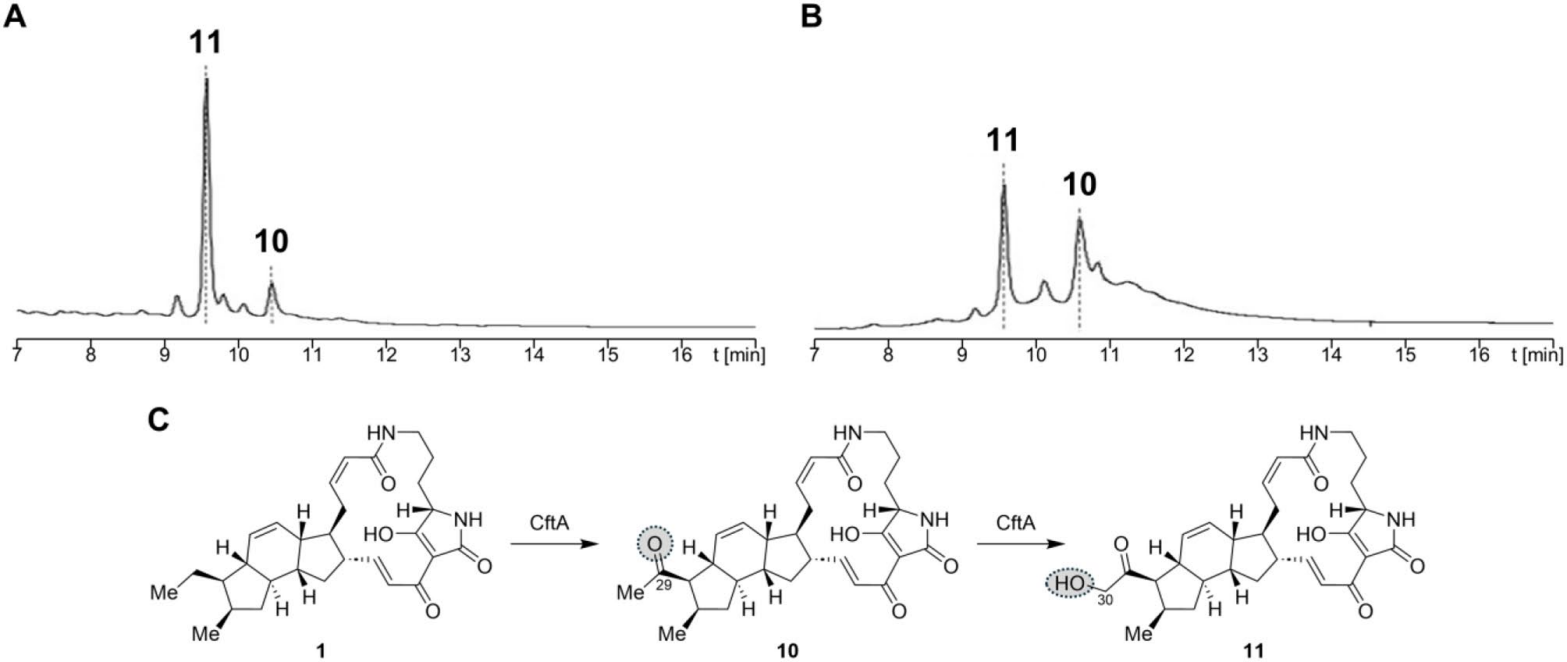
Conversion of **1** to **10** and **11** by CftA. HPLC-UV (220 nm) chromatogram of the extracts of (**A**) medium and (**B**) the cells of *S. albus* DSM 40313. **1** was efficiently converted by CftA to clifednamide A (**10**) and clifednamide C (**11**) using gapdhP(EL) as second promoter. (**C**) Stepwise modification of **1** to **10** and **11** by CftA

**Scheme 1:**
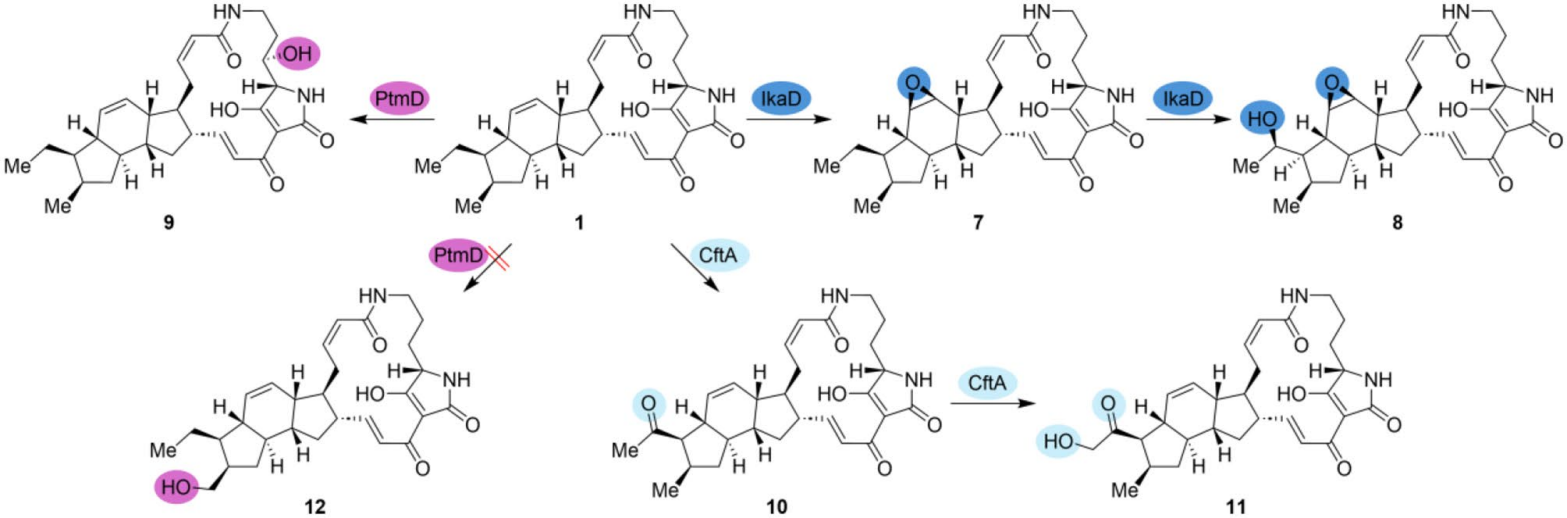
Conversion of **1** by PtmD, IkaD, and CftA. The natural products butremycin (**9**), capsimycin B (**7**), and capsimycin G (**8**), as well as clifednamide A (**10**) and clifednamide C (**11**), were produced by the three modifying enzymes, respectively. Hydroxylation at position C-31 by PtmD was not observed

To apply the gained information about our plug-and-play system, we tested a third modifying gene found in PoTeM BGCs. CftA derives from the clifednamide gene cluster and is a P450 monooxygenase [46]. For the investigation of its function, we only applied a single expression condition (Fig. 5). We used *S. albus*, which was the most productive strain during both previous screenings, and used gapdhP(EL) as second promoter, since this construct yielded the highest conversion with PtmD. The analysis of the corresponding cell extracts showed complete conversion of **1** to two new products **10** (*m/z* 493.2692) and **11** (*m/z* 509.2631). Due to the high expression level, both compounds were readily isolated (**10**: 2 mg/L; **11**: 4–5 mg/L), and their structures were elucidated by 1D and 2D NMR (cf. ESI, Tables S5/S6, Figures S28–S38). In both compounds, a ketone was located at the ethyl moiety at C-29 (Scheme 1). Compound **11** additionally possessed a neighboring hydroxy group at C-30. Bifunctionality and the regioselectivity of CftA, resulting in clifednamide A (**10**) and clifednamide C (**11**), were thus in accordance with previous studies in vivo [27, 47] and in vitro [7, 47]. 

## Discussion

To discover the full diversity of PoTeMs, heterologous expression became irreplaceable, since many BGCs remain silent under laboratory conditions in their native hosts. To set the stage for investigations of new PoTeM BGCs, particularly to study functions of tailoring enzymes, our generated plug-and-play system contains *ikaA* for a reliable production of the uniform PoTeM precursor molecule **5**. Oxidoreductases from all kinds of PoTeM BGCs can be easily introduced and tested for their activity in vivo. For the establishment of the system, the production of **1** using *ikaBC* was employed as a first proof of this method. Furthermore, modifying enzymes can be introduced to this vector downstream of a second promoter. Thereby, the functionality of the bifunctional monooxygenases IkaD and CftA was confirmed and the function of PtmD was reassigned as C-3 PoTeM hydroxylase (Scheme 1). Previous studies have already shown that it can be beneficial to replace the native iPKS/NRPS by a well-studied homologous version to ensure production of a sufficient amount of the central precursor **5** [20, 21, 23]. Within this work, we additionally show that different promoters controlling genes encoding tailoring enzymes can dramatically influence modified PoTeM production yields. Besides the broadly used kasOP*, P-31 and SF14P yielded good gene expression of PoTeM modifying genes. Surprisingly, we observed differences between the expression of the monooxygenase *ikaD* and the hydroxylase *ptmD*. For *ikaD*, also the promoter P-15 yielded a high conversion of **1** to **7** and **8** (Scheme 1). Compound **1** was almost completely converted by the expression of the cytochrome P450 monooxygenases (IkaD and CftA) through our system.

However, the promoters gapdhP(EL) and rpsLP(XC) were inactive for *ikaD* but proved to be the most active for *ptmD*. This highlights the value of testing various promoters to optimize the activity of tailoring enzymes and maximize turnover efficiency. One reason for the different outcomes when using different tailoring-enzyme-encoding gene versus promoter combinations might be the exact timing and duration of individual promoter activities at different growth phases of the heterologous host system: for rather unstable products, such as epoxides **7** and **8**, late-stage activity might be most beneficial, whereas more stable products are produced at higher levels for promoters with early and/or long-lasting activity. Furthermore, the plug-and-play system was successfully used in different *Streptomyces* strains. *S. albus* DSM40313 was generally found to be the most productive strain, whereas *S. lividans* TK24 barely produced the expected PoTeMs. *S. coelicolor* M1154 also yielded decent amounts and had slightly less side-products (cf. Figure S5), which potentially facilitates product isolation from complicated product mixtures. Overall, recombinant production of PoTeMs seems to be best performed in *S. albus* host strains, with promoters being selected based on the stability of the expected products, with kasOP* or P-31 being ideally suited for rather unstable and gapdhP(EL) or rpsLP for stable products. While this work nicely demonstrates the utility of the established plug-and-play system for late-stage functionalization of ikarugamycin (**1**) by oxygenation, further tailoring and also different PoTeM cyclization enzymes remain to be tested. In this context, it will be particularly important to expand its applicability to PoTeM biosynthetic genes from Gram-negative organisms. As such genes have successfully been utilized in *Streptomyces* recombinant hosts in the past, e.g., for Gram-negative *Pseudoalteromonas* sp. [20], the extension of the plug-and-play system to such applications should readily be possible.

## Conclusions

Overall, we established a genetic plug-and-play system that readily facilitated the production of oxidatively modified PoTeM analogs by late-stage functionalization of **1** using IkaD (products **7** and **8**), CftA (**10** and **11**), and PtmD (**9**) (Scheme 1). This system and our insights into production levels in three different *Streptomyces* sp. host strains combined with several constitutive promoters can now be further applied to the investigation of new PoTeM BGCs and/or modifying enzymes, making novel PoTeM derivatives accessible for biological activity screenings.

## Electronic supplementary material

Below is the link to the electronic supplementary material.

Supplementary Material 1

## Data Availability

All data generated or analyzed in this study are included in this published article [and its supplementary information files]. All raw data files are available from the corresponding author on request.

## Abbreviations

ADH: Nicotinamide-cofactor-dependent alcohol dehydrogenase
apra: Apramycin
BGC: Biosynthetic gene cluster
cam: Chloramphenicol
DiPaC: Direct pathway cloning
iPKS: Iterative polyketide synthase
kan: Kanamycin
LB: Lysogeny broth
NA: Nalidixic acid
NRPS: Non-ribosomal peptide synthetase
PhyDH: Flavin-dependent phytoene dehydrogenase homolog
PoTeM: Polycyclic tetramate macrolactam
TB: Terrific broth
