# Supplementary material for: A plug-and-play system for polycyclic tetramate macrolactam production and functionalization
Source: Microb Cell Fact. 2025 Jan 10;24:13. doi: 10.1186/s12934-024-02630-8 (PMC11724479; doi:10.1186/s12934-024-02630-8)
Supplement: Supplementary file 1 — Supplementary Material 1 [file 12934_2024_2630_MOESM1_ESM.pdf]

Supplementary Information

A Plug-and-Play System for Polycyclic Tetramate Macrolactam Production and Functionalization

Anna Glöckle[a,ǂ], Sebastian Schuler[a,ǂ], Manuel Einsiedler[a,b], Tobias A. M. Gulder[a,b,*]

[a] Chair of Technical Biochemistry, Technische Universität Dresden, Bergstraße 66, 01069 Dresden, Germany.
[b] Helmholtz Institute for Pharmaceutical Research Saarland (HIPS), Department of Natural Product Biotechnology, Helmholtz Centre for Infection Research (HZI) and Department of Pharmacy at Saarland University, PharmaScienceHub (PSH), Campus E8.1, 66123, Saarbrücken, Germany.
ǂ Contributed equally to this work.
*E-mail: tobias.gulder@helmholtz-hips.de

Contents

1. Analysis of the basic plug-and-play system 2
2. Cloning strategy to establish the plug-and-play system 3
3. Details on the analysis of the extracts 4
4. HR-MS analysis of the produced PoTeMs 7
5. NMR analysis of the produced PoTeMs 9
   Ikarugamycin (1) 9
   Capsimycin G (8) 13
   Butremycin (9) 17
   Clifednamide A (10) 22
   Clifednamide C (11) 26
6. Bacterial strains, primer, plasmids, and sequences 30
   Strains 30
   Primers 30
   Plasmids 31
   Gene sequences 33
   Sequences of the used promoters 37
7. Cloning 39
8. Literature 55

1. Analysis of the basic plug-and-play system

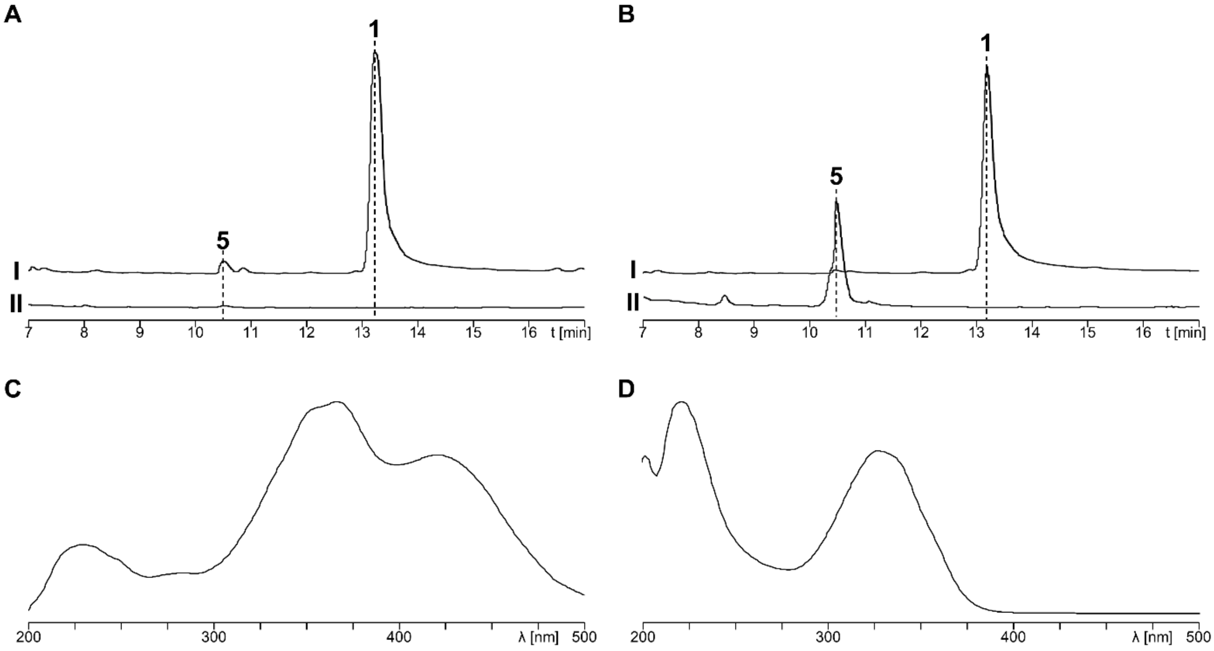

Figure S1. Analysis of the PoTeMs produced by the basic plug-and-play constructs. The plasmids pSET152-ermE*::*ikaABC* gapdhP(EL) (**I**) and pSET152-ermE*::*ikaA*-gapdhP(EL) (**II**) were expressed in *S. albus* DSM 40313. The extracts of **A.** the medium and **B.** the cells were analyzed by HPLC-UV (**I**: 220 nm and **II**: 365 nm). Both plasmids result in the production of lysobacterene A (**5**). Within the cells, IkaBC efficiently converted **5** into ikarugamycin (**1**), which subsequently diffused into the medium. The yield of lysobacterene A (**5**) was limited due to its instability. Harsh extraction conditions and exposure to light can lead to the degradation of compound **5**, especially in the extracts of the medium (**A**). The UV spectra of **C.** lysobacterene A (**5**) and **D.** ikarugamycin (**1**) are shown.

2. Cloning strategy to establish the plug-and-play system

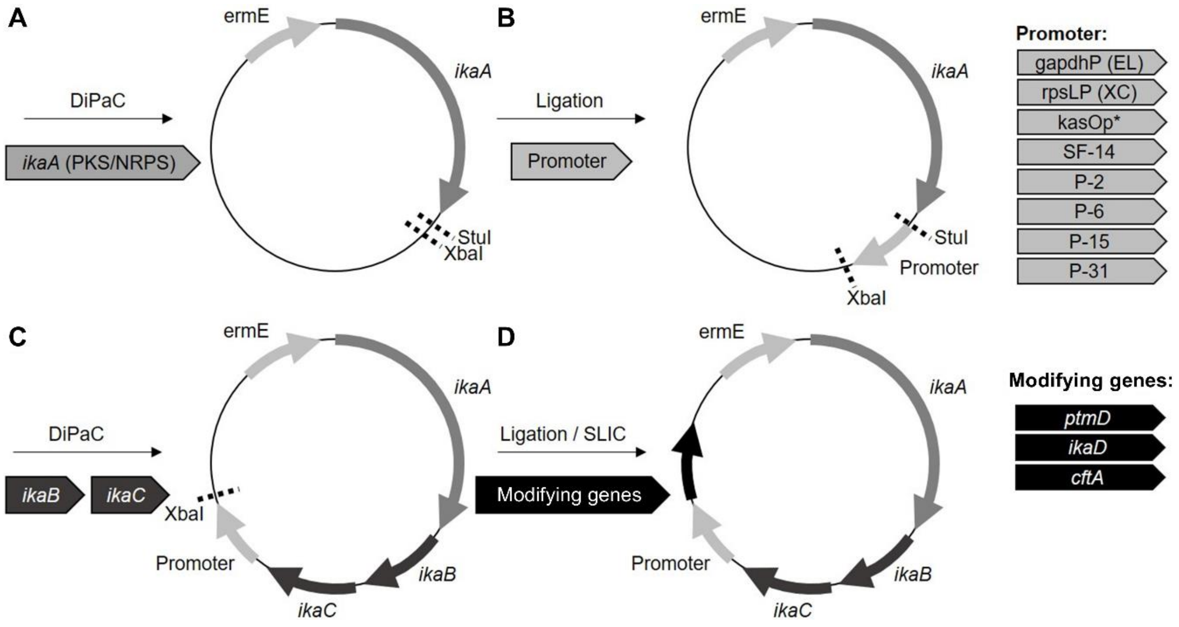

Figure S2. Cloning strategy to establish the PoTeM plug-and-play system. **A.** The iPKS/NRPS gene *ikaA* was introduced into pSET152-ermE* by DiPaC. **B.** Eight different promoters were added to build the basic eight expression vectors for this plug-and-play system by ligation cloning. **C.** The two additional genes from the biosynthetic gene cluster encoding **1** (*ikaBC*) were added by DiPaC. **D.** The expression system was finalized by adding three different modifying genes (*ptmD*, *ikaD*, and *cftA*) by ligation cloning.

3. Details on the analysis of the extracts

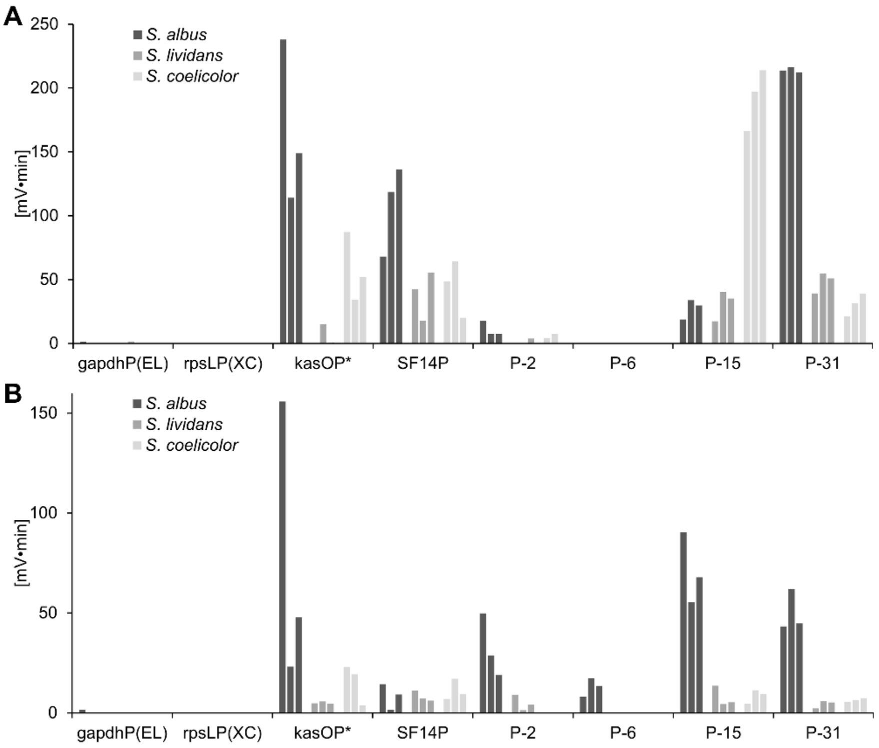

Figure S3. Detailed analysis of the conversion of **1** by IkaD using different promoters and *Streptomyces* expression hosts. Compound **8** was found in the extracts of **A.** the medium and **B.** the cells. *S. albus* DSM 40313 (black), *S. lividans* TK24 (dark grey), and *S. coelicolor* M1154 (light grey).

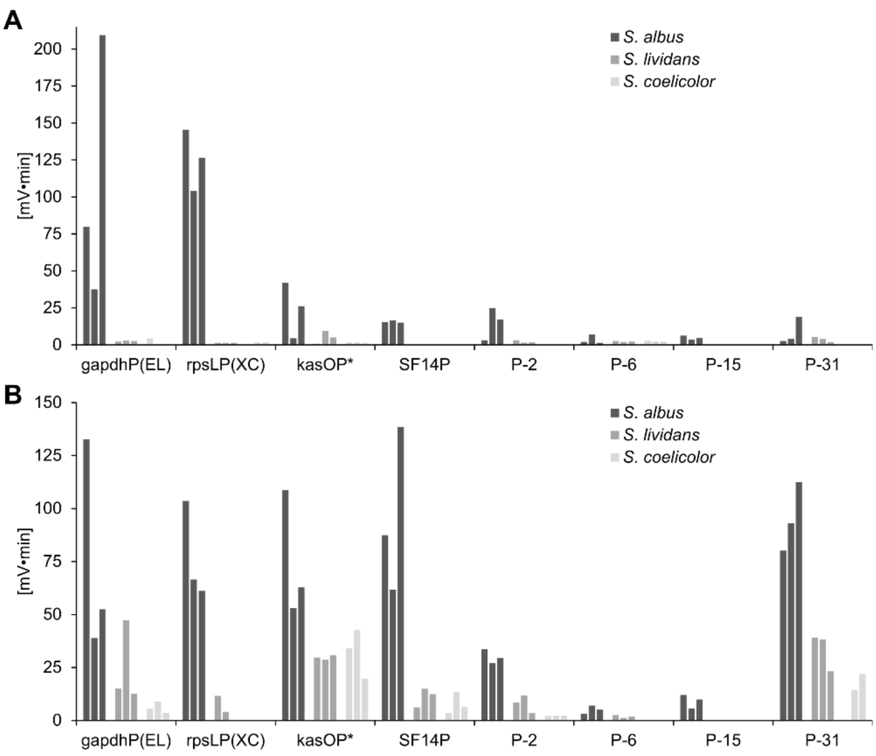

Figure S4. Detailed analysis of the conversion of **1** by IkaD using different promoters and *Streptomyces* expression hosts. Compound **9** was found in the extracts of **A.** the medium and **B.** the cells. *S. albus* DSM 40313 (black), *S. lividans* TK24 (dark grey), and *S. coelicolor* M1154 (light grey).

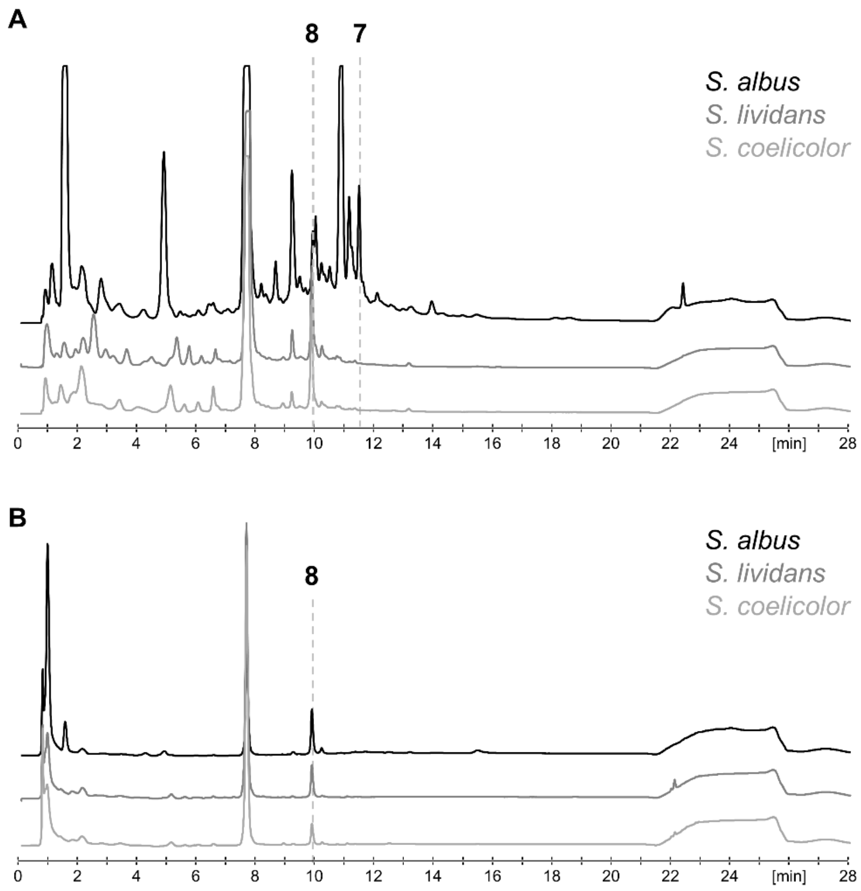

Figure S5. Comparison of the expression of pSET152-ermE*-*ikaABC*-SF14P-*ikaD* in three different expression hosts. **A.** Extract of the medium and **B.** cell extracts. *S. albus* DSM 40313 (black), *S. lividans* TK24 (dark grey), and *S. coelicolor* M1154 (light grey)

4. HR-MS analysis of the produced PoTeMs

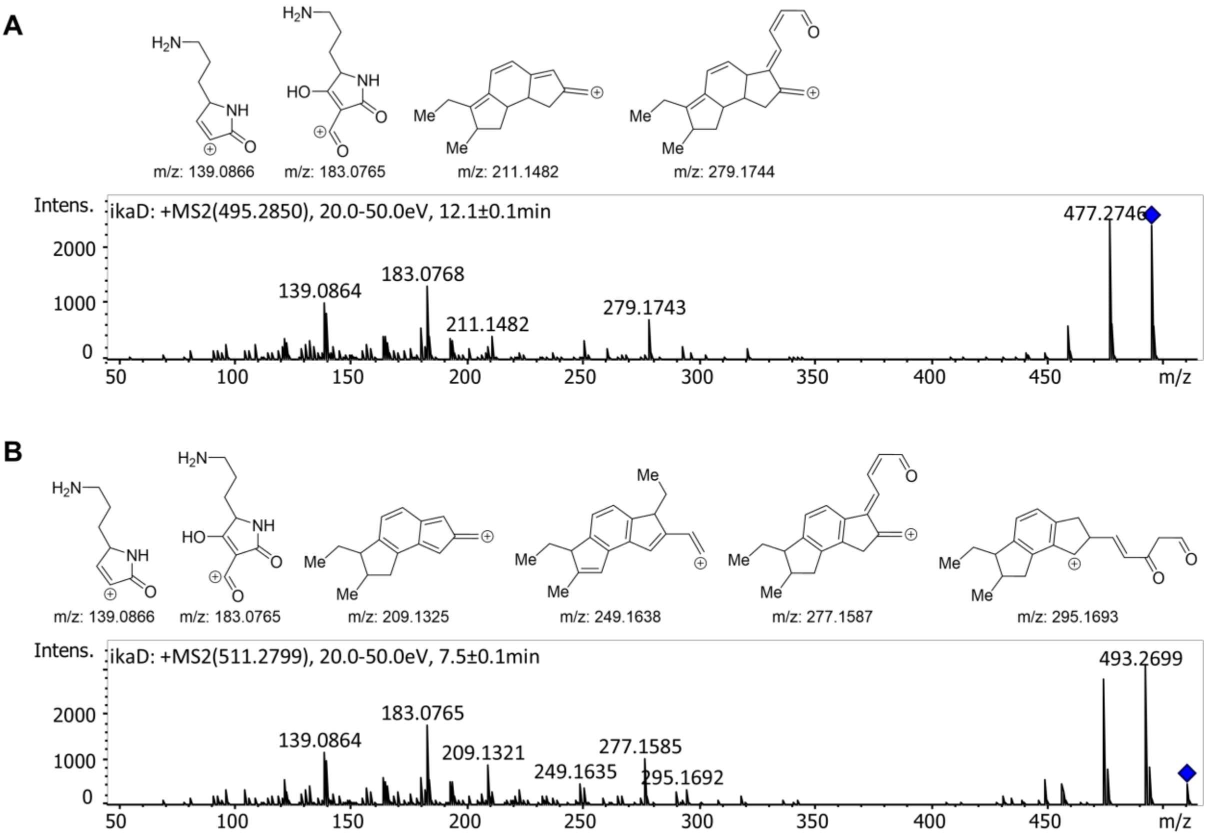

Figure S6. MS/MS analysis of the PoTeM modified by IkaD. **A.** *m/z* found: 495.2850, representing **7** ($[M+H]^+$ calculated *m/z* 495.2853). **B.** *m/z* found: 511.2799, representing **8** ($[M+H]^+$ calculated *m/z* 511.2803). Blue squares label mother ions.

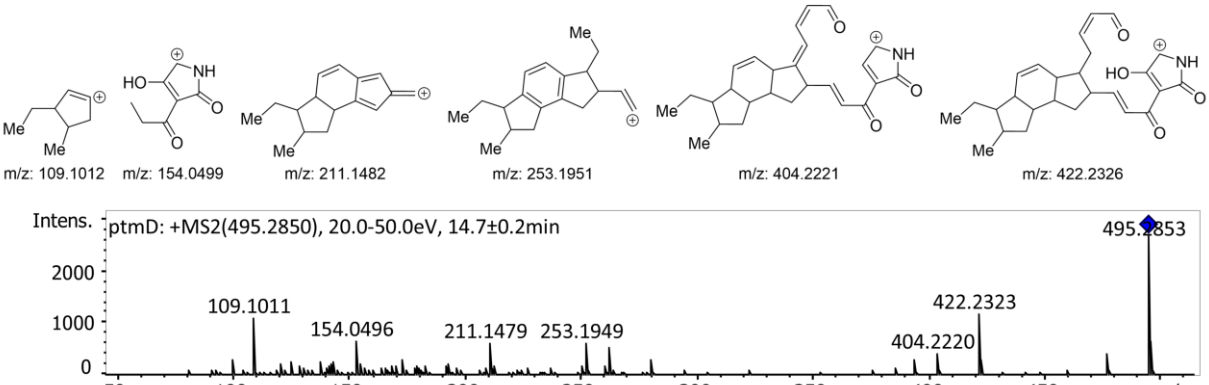

Figure S7. MS/MS analysis of the PoTeM modified by PtmD. *m/z* found: 495.2850, representing **9 (**$[M+H]^+$ calculated *m/z* 495.2853). Blue square labels mother ion.

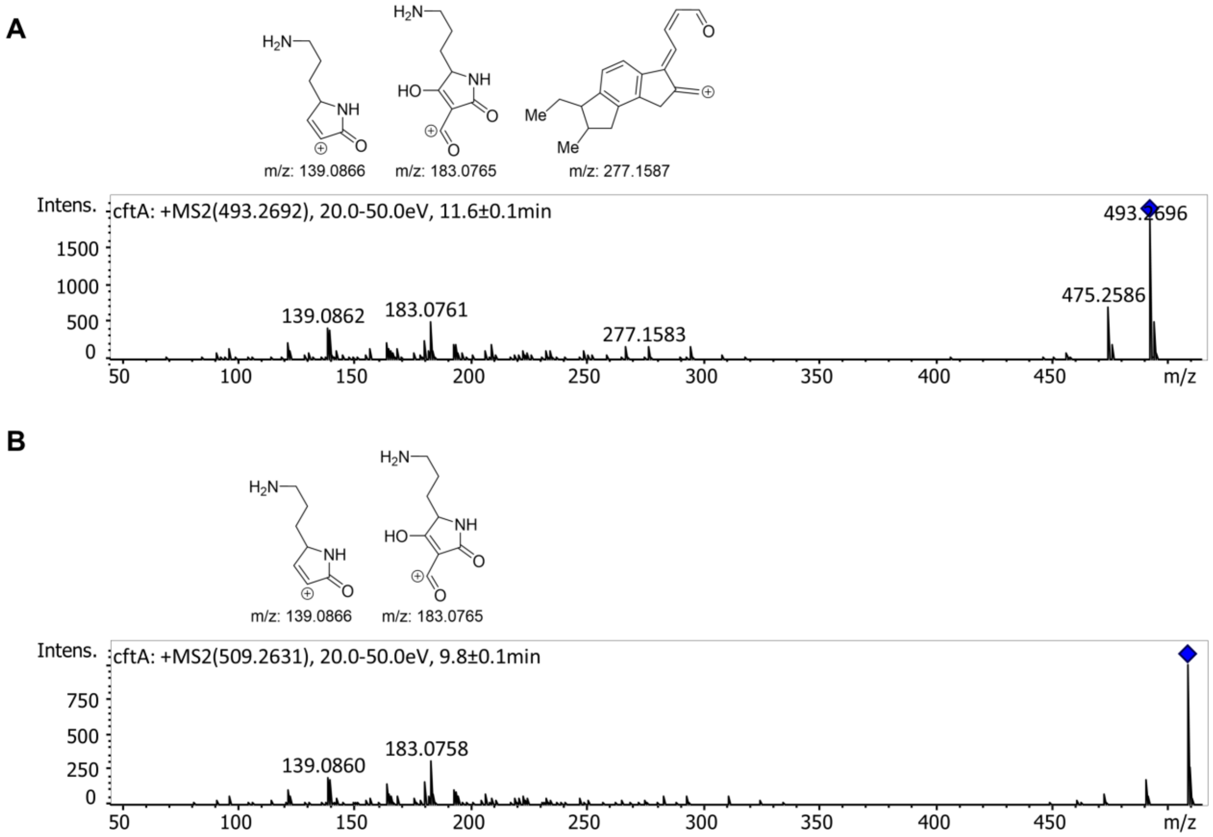

Figure S8. MS/MS analysis of the PoTeM modified by CftA. **A.** *m/z* found: 493.2692, representing **10** ($[M+H]^+$ calculated *m/z* 493.2697). **B.** *m/z* found: 509.2631, representing **11** ($[M+H]^+$ calculated *m/z* 509.2646). Blue squares label mother ions.

5. NMR analysis of the produced PoTeMs

Ikarugamycin (1)

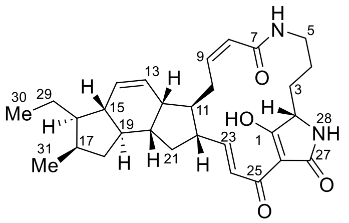

Table S1. ^{1}H, ^{13}C NMR (600 MHz, DMSO-d_6) of ikarugamycin (**1**).

	**δ_C**	**δ_H (m, *J*)**	**COSY**
1	195.9	/	
2	61.1	3.83 (d, 5.9 Hz)	3
3	26.8	1.88–1.81 (m), 1.75–1.69 (m)	2, 3, 4
4	20.4	1.40–1.35 (m), 1.10–1.03 (m)	3, 4, 5
5	38.2	3.25–3.20 (m), 2.48–2.42 (m)	4, 5, 6
6	/	7.87 (t, 5.6 Hz)	5
7	165.5	/	
8	124.3	5.76 (d, 12.7 Hz)	9, 10
9	139.3	5.98 (td, 11.1, 3.8 Hz)	8, 10
10	24.8	3.56–3.51 (m), 2.28–2.19 (m)	8, 9, 10, 11
11	48.2	1.55–1.45 (m)	10, 22
12	42.6	2.53–2.49 (m)	13, 14, 20
13	128.8	5.73 (dt, 9.9, 2.8 Hz)	12, 14, 15
14	130.6	5.91 (dt, 2.1 Hz)	12, 13, 15
15	46.7	1.56–1.50 (m)	13, 14, 16, 19
16	46.5	1.34–1.28 (m)	15, 17
17	32.5	2.28–2.19 (m)	16, 18, 31
18	38.1	0.66 (td, 12.0, 6.9 Hz),<br>2.09 (dt, 12.8, 7.8 Hz)	17, 18, 19
19	48.3	1.20–1.11 (m)	15, 18, 20
20	41.2	2.06–2.00 (m)	12, 19
21	36.1	2.06–2.00 (m), 1.28–1.19 (m),	21, 22
22	49.6	2.32–2.41 (m)	11, 21, 23
23	150.3	6.64 (dd, 15.5, 10.2 Hz)	22, 24
24	122.0	6.98 (d, 15.5 Hz)	23
25	171.4	/	
26	100.8	/	
27	175.2	/	
28	/	8.69 (s)	
29	21.1	1.50–1.42 (m), 1.34–1.28 (m),	30
30	13.2	0.91 (t, 7.2 Hz)	29
31	17.7	0.86 (d, 7.1 Hz)	17

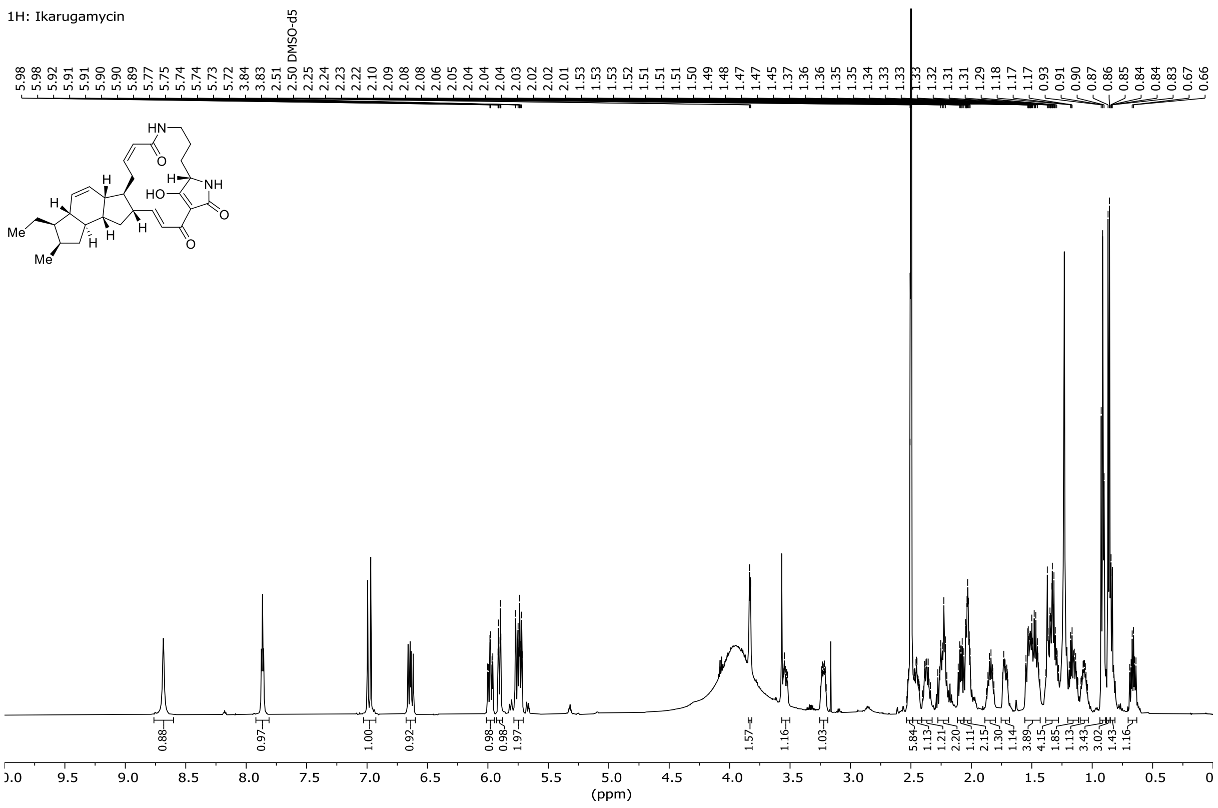

Figure S9. ^{1}H NMR of ikarugamycin (**1**).

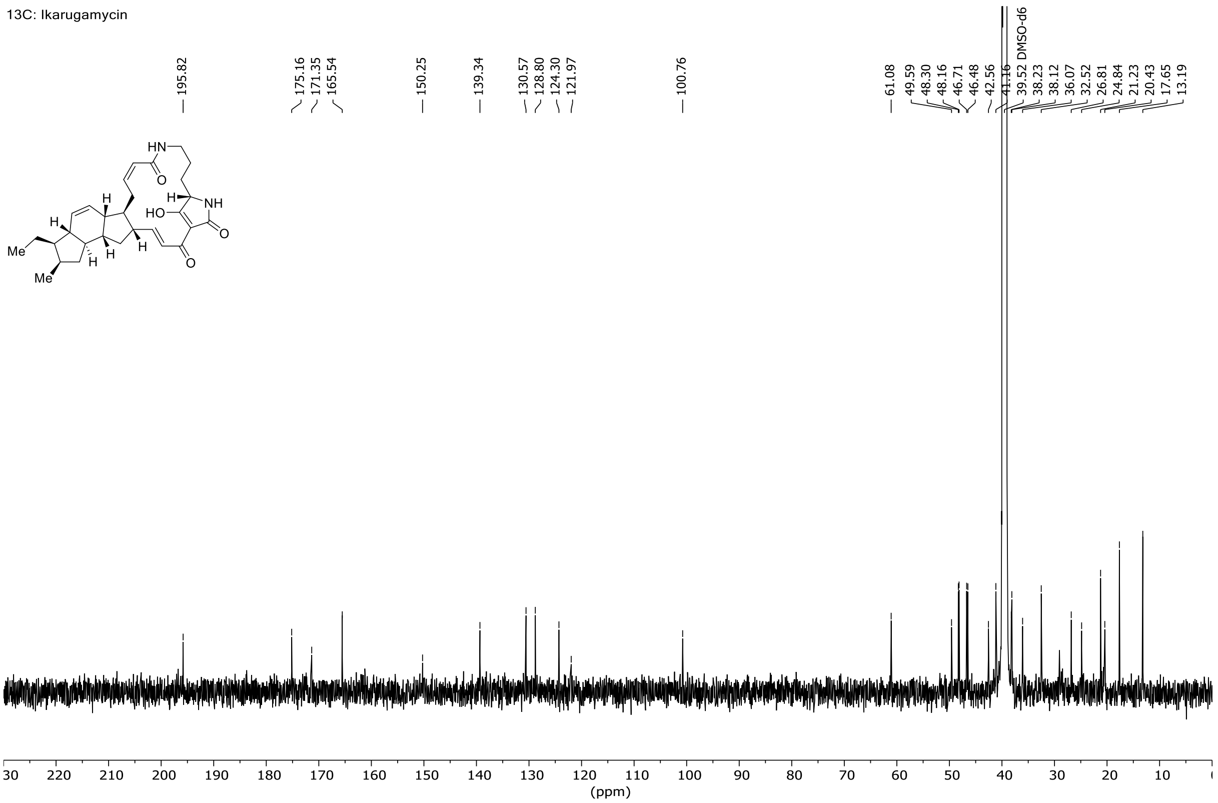

Figure S10. $^{13}C\{^{1}H\}$ NMR of ikarugamycin (**1**).

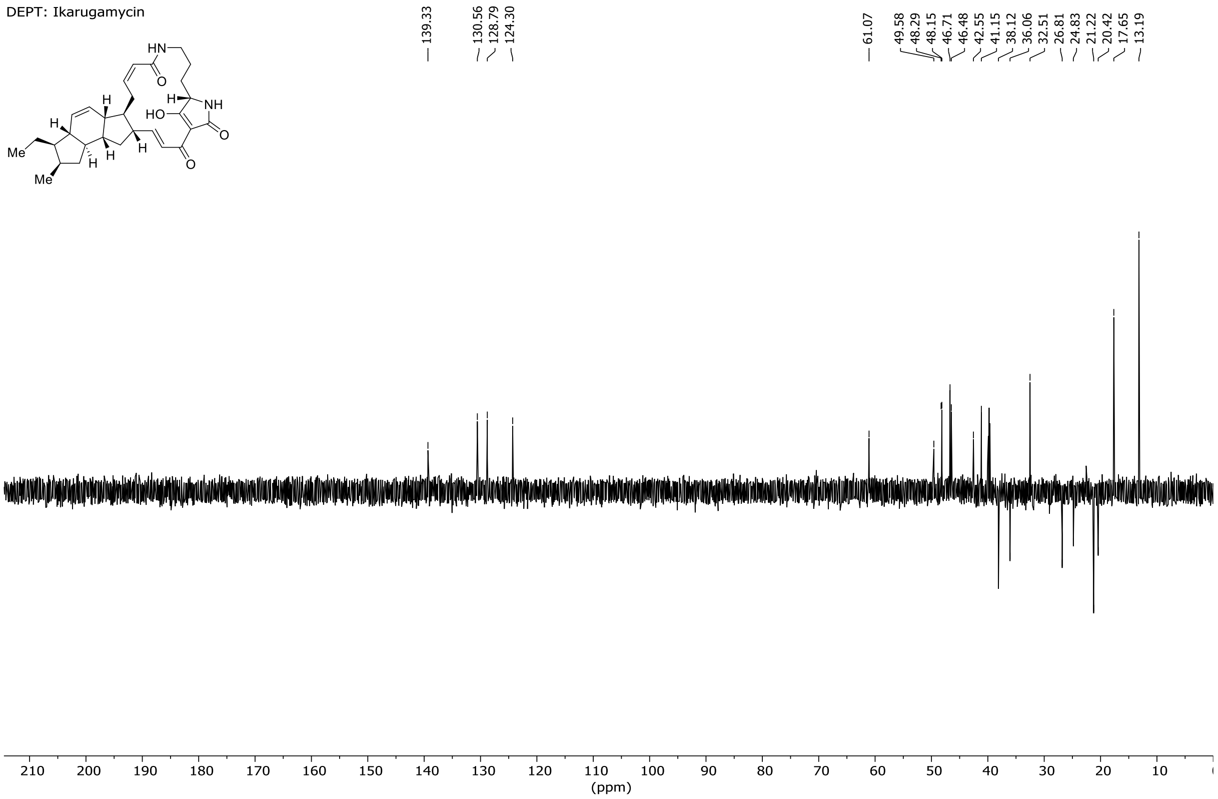

Figure S11. ^{13}C DEPT NMR of ikarugamycin (**1**).

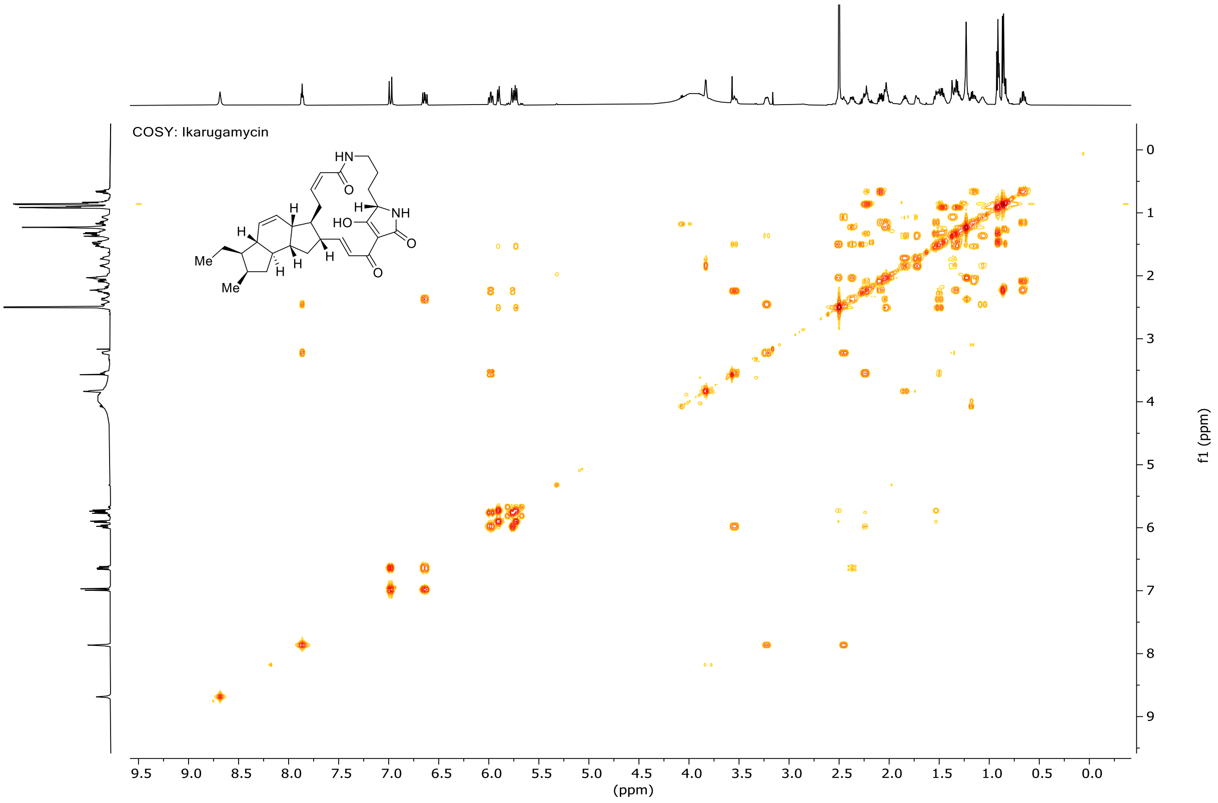

Figure S12. ^{1}H–^{1}H-COSY NMR of ikarugamycin (**1**).

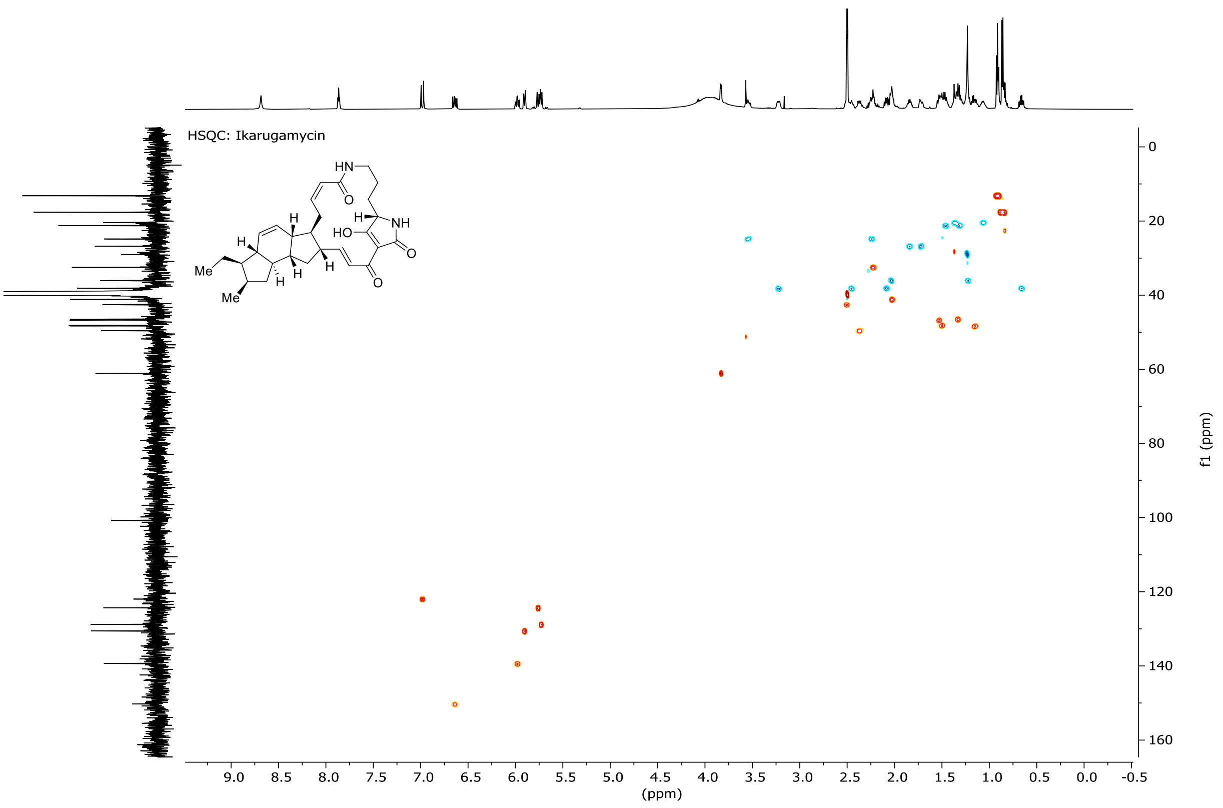

Figure S13. ^{1}H–^{13}C-HSQC NMR of ikarugamycin (**1**).

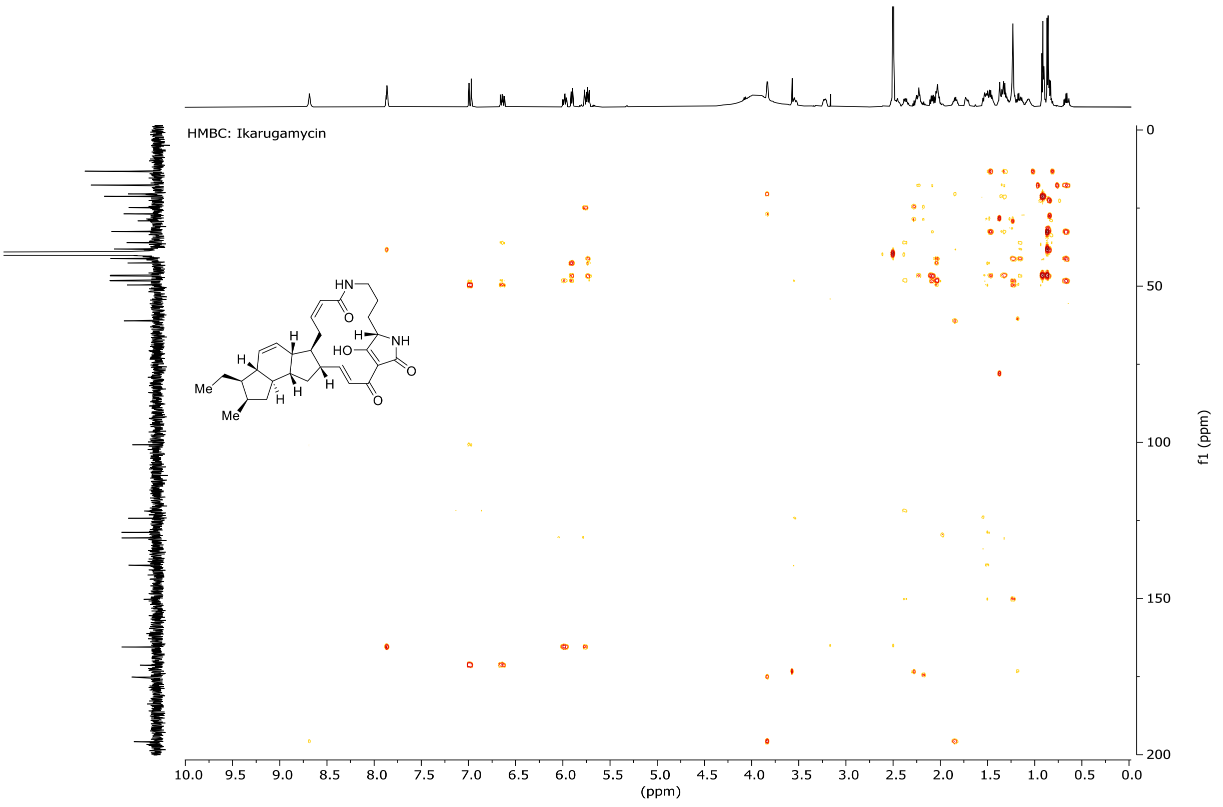

Figure S14. ^{1}H–^{13}C-HMBC NMR of ikarugamycin (**1**).

Capsimycin G (8)

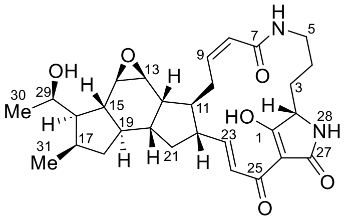

Table S2. ^{1}H, ^{13}C NMR (600 MHz, DMSO-d_6) of capsimycin G (**8**).

	δ_C	δ_H (m, *J*)	COSY
1	195.9	/	
2	61.1	3.83 (bd, 5.6 Hz)	3
3	26.8	1.89–1.81 (m), 1.76–1.69 (m)	2, 4
4	20.4	1.40–1.32 (m), 1.11–1.03 (m)	3, 5
5	38.2	3.27–3.20 (m), 2.48–2.41 (m)	4, 6
6	/	7.89 (t, 5.6 Hz)	5
7	165.5	/	
8	124.4	5.79 (d, 11.6 Hz)	9, 10
9	139.0	6.03 (td, 11.0, 3.5 Hz)	8, 10
10	25.2	3.68–3.61 (m), 2.35–2.28 (m)	8, 9, 11
11	45.4	1.71–1.64 (m)	10, 12, 22
12	40.0	2.32–2.26 (m)	11, 13, 20
13	53.0	2.90 (d, 4.0 Hz)	12, 14
14	57.4	3.18 (dd, 4.0, 1.7 Hz)	13, 15
15	46.7	0.87 (td, 11.9, 2.0 Hz)	14, 16, 19
16	51.4	1.76 (dt, 11.4, 9.4 Hz)	15
17	33.2	2.29–2.22 (m)	18, 31
18	38.7	2.01–1.94 (m), 0.58 (td, 11.9, 8.4 Hz)	17, 19
19	47.1	1.12 (qd, 11.6, 6,6)	15, 18, 20
20	40.7	1.69–1.62 (m)	12, 19
21	36.3	2.02–1.95 (m), 1.19–1.12 (m)	22
22	48.9	2.34 (qd, 10.7, 7.7 Hz)	11, 21, 23
23	149.8	6.64 (dd, 15.5, 10.3 Hz)	22, 24
24	122.2	7.00 (d, 15.6 Hz)	23
25	171.1	/	
26	100.9	/	
27	175.1	/	
28	/	8.69 (bs)	
29	66.5	3.70 (dq, 8.8, 6.2 Hz)	
30	23.4	1.22 (d, 6.2 Hz)	
31	18.2	1.01 (d, 7.1 Hz)	17

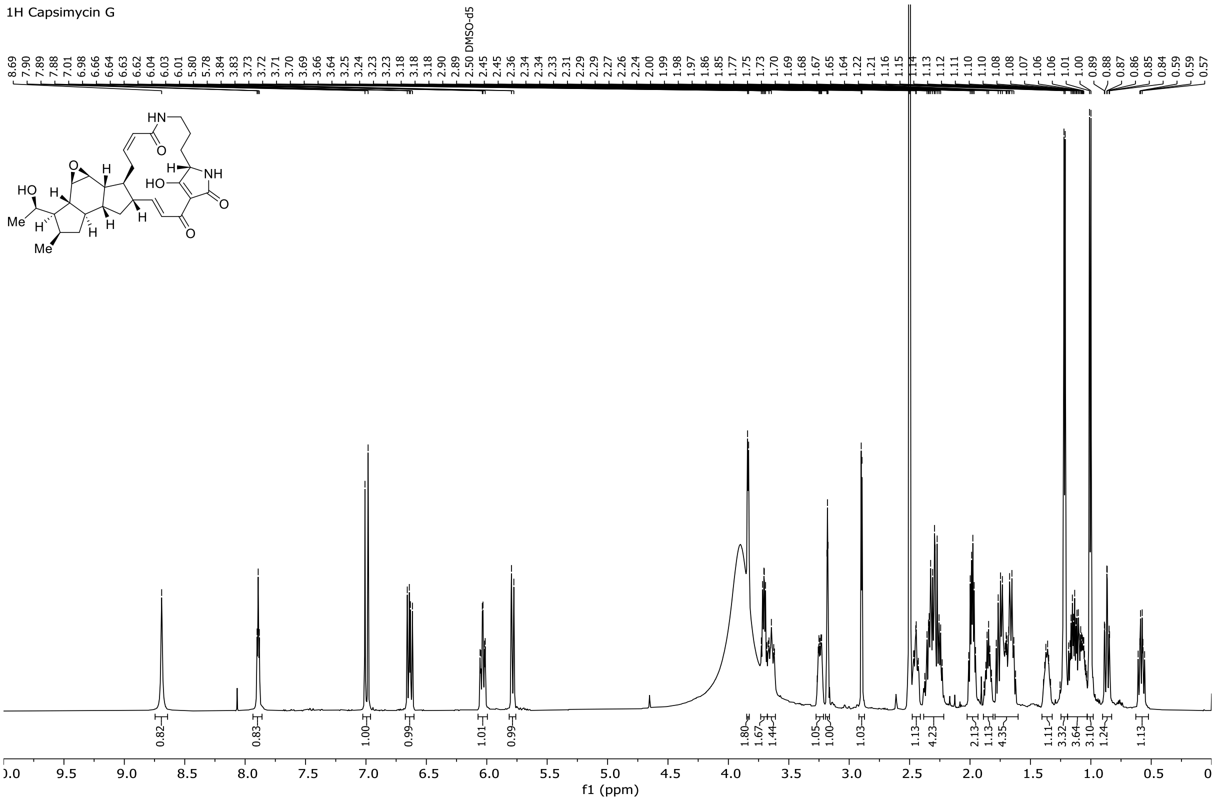

Figure S15. ^{1}H NMR of capsimycin G (**8**).

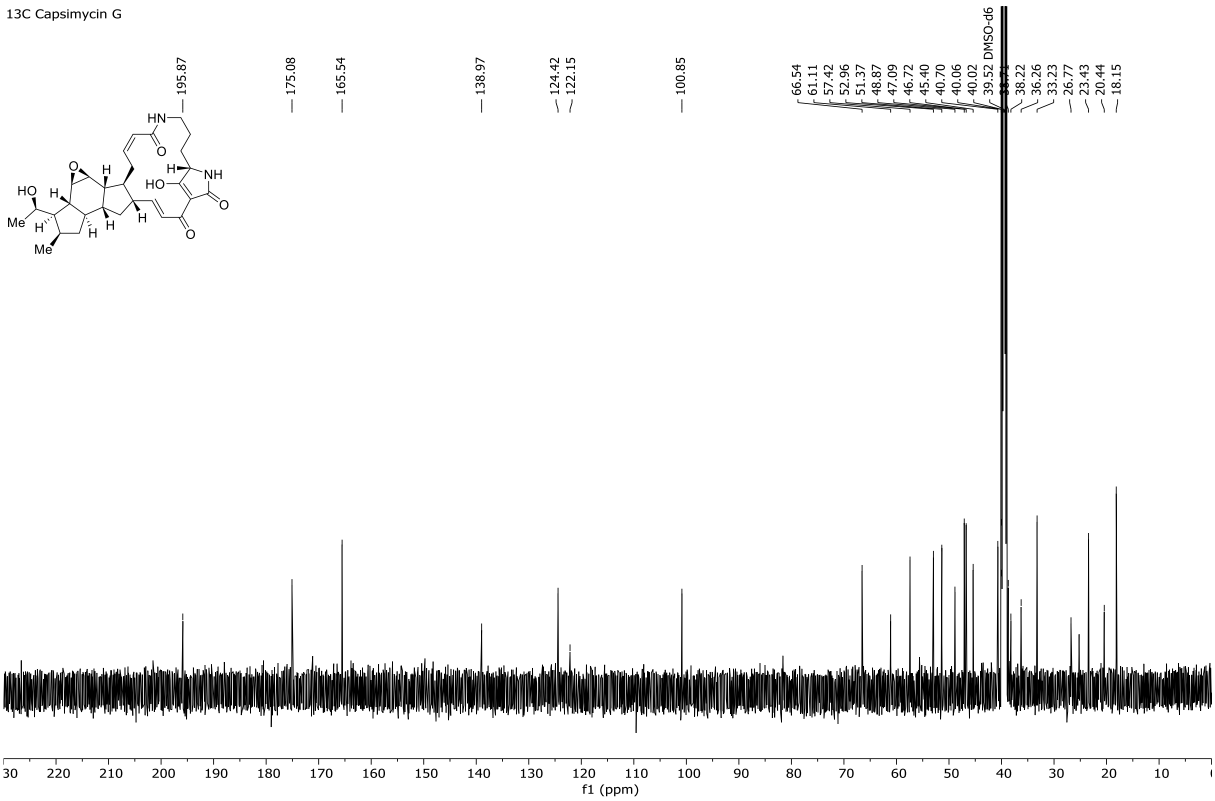

Figure S16. $^{13}C\{^{1}H\}$ NMR of capsimycin G (**8**).

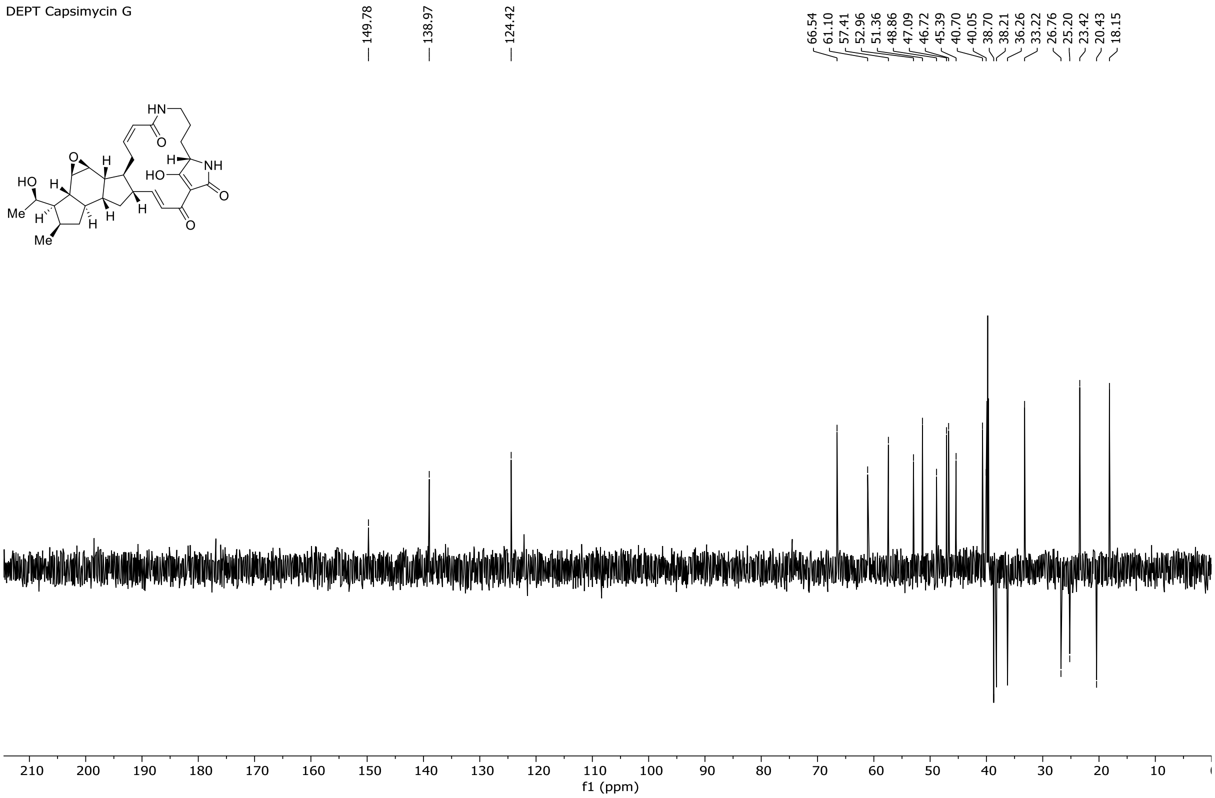

Figure S17. $^{13}C\{^{1}H\}$ DEPT NMR of capsimycin G (**8**).

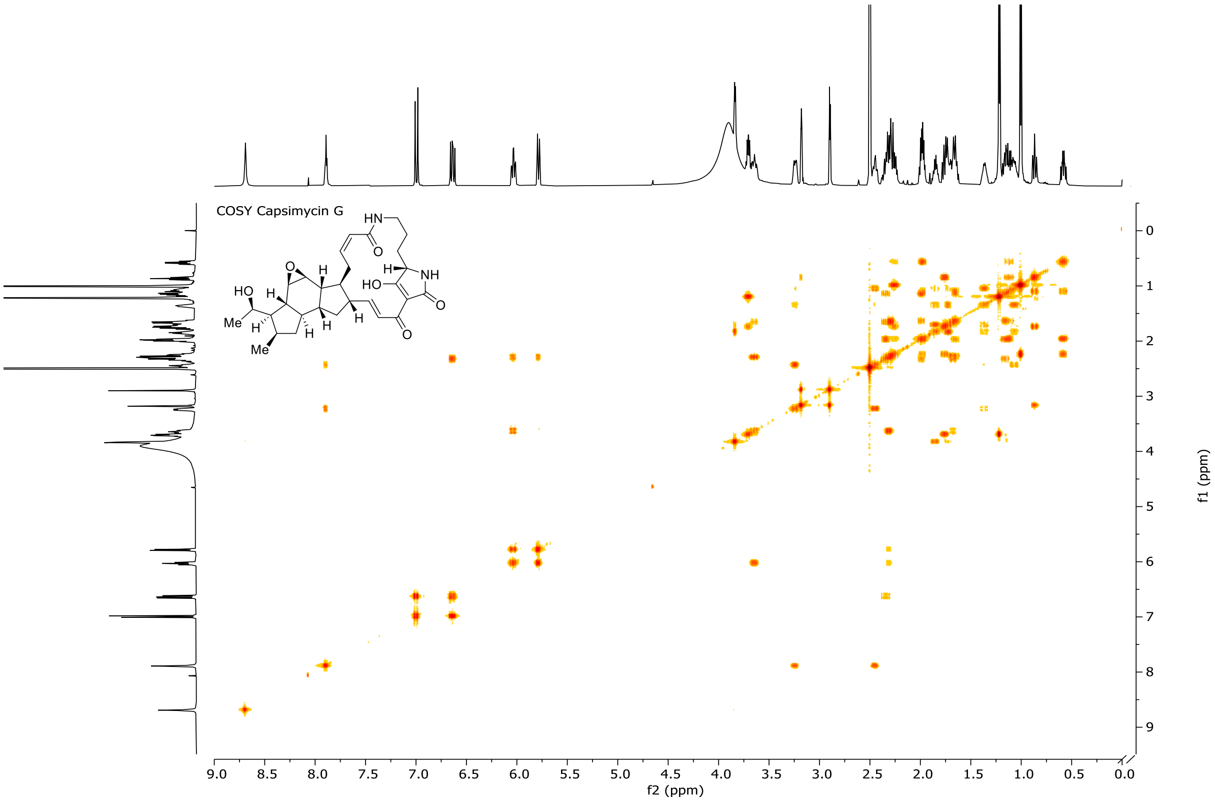

Figure S18. ^{1}H–^{1}H-COSY NMR of capsimycin G (**8**).

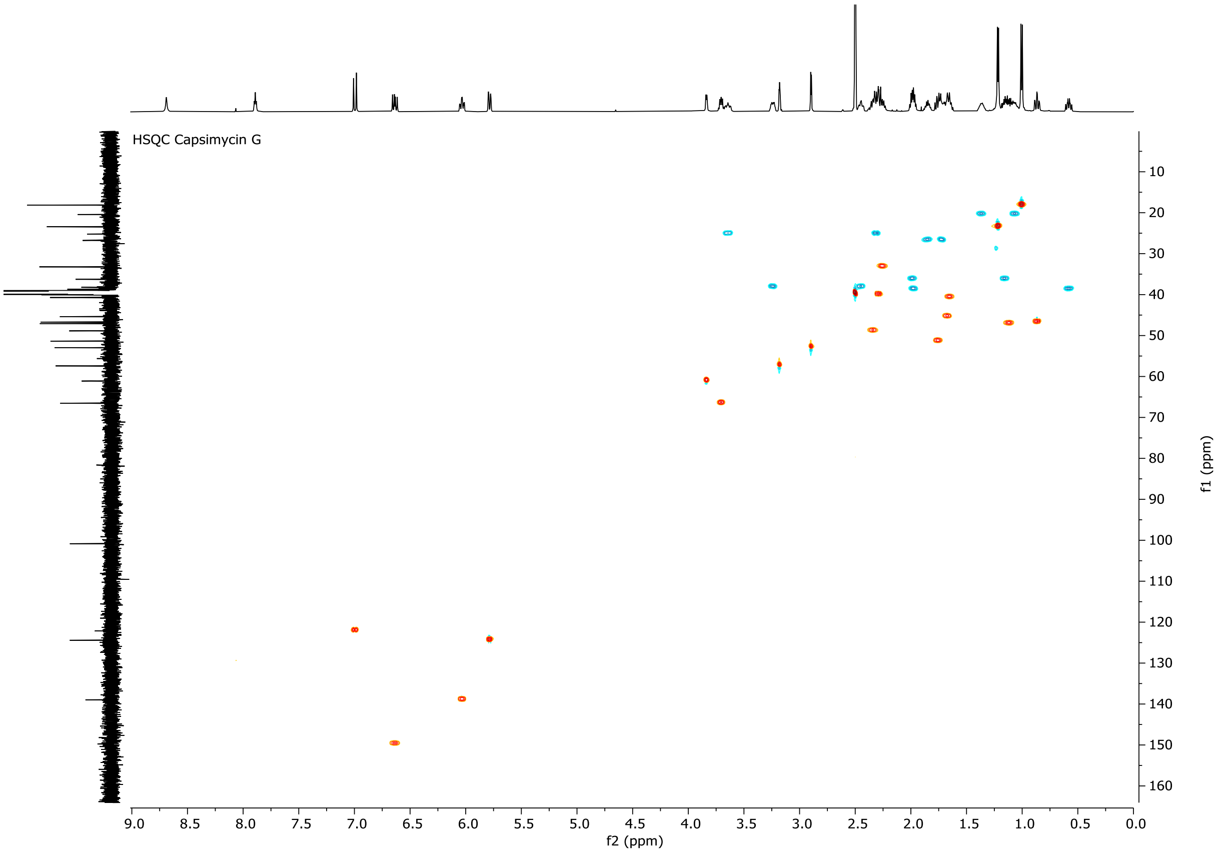

Figure S19. ^{1}H–^{13}C-HSQC NMR of capsimycin G (**8**).

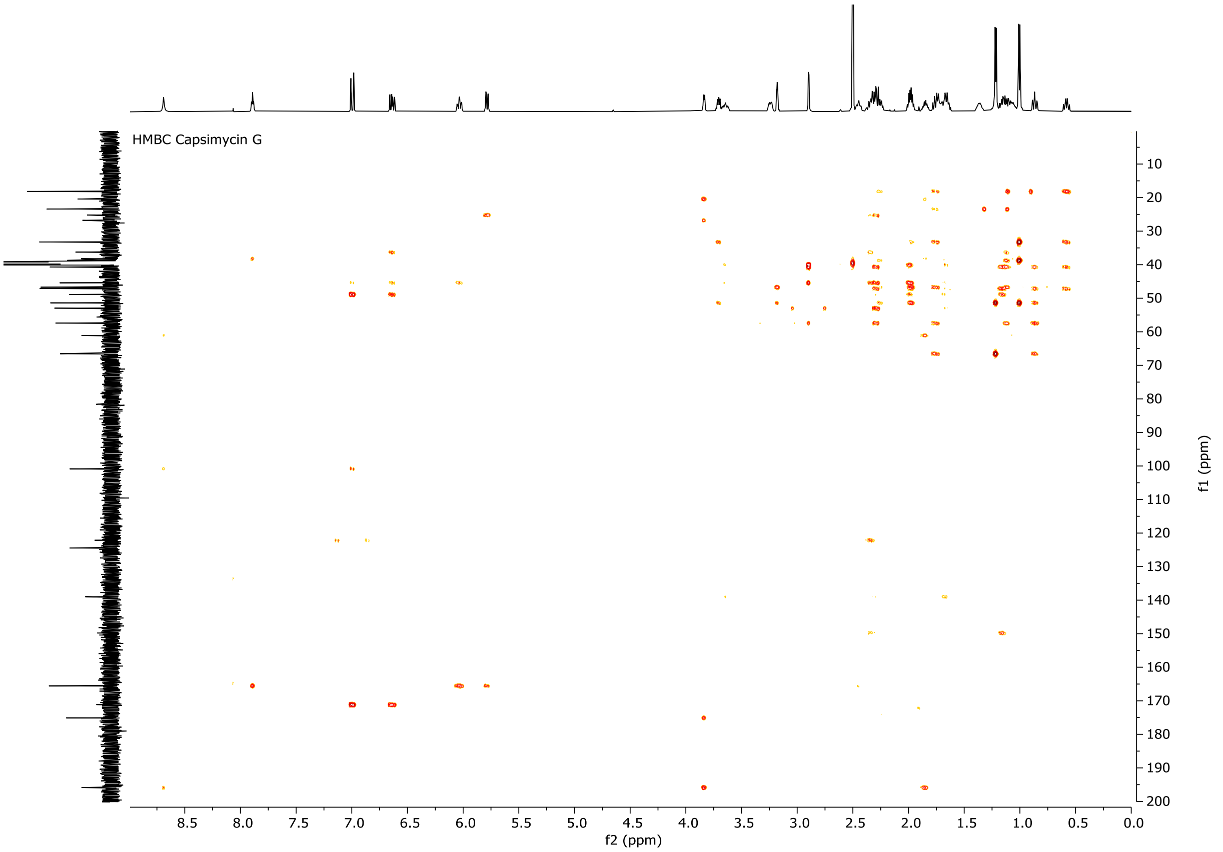

Figure S20. ^{1}H–^{13}C-HMBC NMR of capsimycin G (**8**).

Butremycin (9)

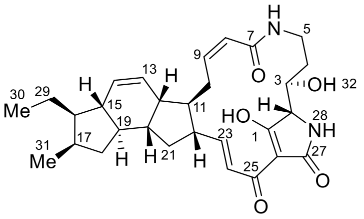

Table S3. ^{1}H, ^{13}C NMR (500 MHz, DMSO-d_6) of butremycin (**9**).

	δ_C	δ_H (m, *J*)	COSY
1	ND	/	
2	68.3*	3.87 (bs)	
3	70.3*	3.82–3.78 (m)	4, 32
4	31.0*	1.38–1.28 (m), 1.25–1.16 (m)	3, 5
5	36.2*	3.34–3.25 (m), 2.64–2.56 (m)	4, 5, 6
6	/	7.98 (t, 5.7 Hz)	5
7	165.2**	/	
8	124.2*	5.75 (d, 10.3 Hz)	9, 10
9	139.1*	6.01 (td, 11.2, 3.0 Hz)	8, 10
10	24.4*	3.70–3.60 (m), 2.17–2.08 (m)	8, 9, 11
11	48.1*	1.56–1.44 (m)	10
12	42.0*	2.56–2.50 (m)	13, 14, 20
13	128.7*	5.72 (dt, 9.9, 3.0 Hz)	12, 14, 15
14	130.5*	5.90 (d, 9.9 Hz)	12, 13, 15
15	46.8*	1.56–1.44 (m)	13, 14, 16
16	46.4*	1.38–1.28 (m)	15, 17
17	32.4*	2.26–2.21 (m)	16, 31
18	38.1*	2.06–2.16 (m), 0.67 (td, 12.0, 6.8 Hz)	18, 19
19	48.3*	1.19–1.11 (m)	18
20	41.1*	2.06–1.97 (m)	12
21	36.0*	2.06–1.97 (m), 1.38–1.28 (m)	22
22	49.2*	2.38–2.31 (m)	21, 23
23	150.4*	6.67–6.60 (m)	22, 24
24	121.9*	6.91 (d, 15.5 Hz)	23
25	ND	/	
26	ND	/	
27	ND	/	
28	/	8.87 (s)	
29	21.1*	1.51–1.44 (m), 1.34–1.28 (m)	30
30	13.1*	0.92 (t, 7.0 Hz)	29
31	17.6*	0.86 (d, 7.1 Hz)	17
32	/	5.09 (bs)	3

* ^{13}C shift taken from HSQC. ** ^{13}C shift taken from HMBC. ND not detected.

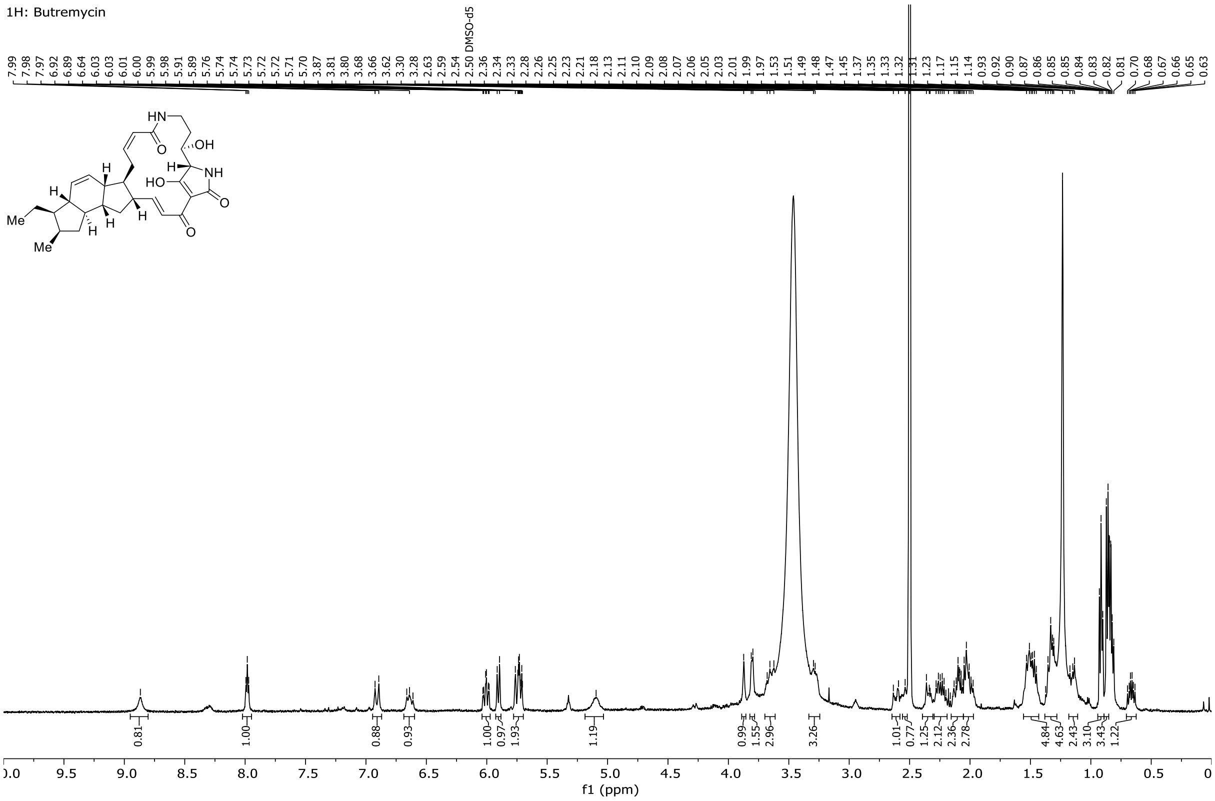

Figure S21. ^{1}H NMR of butremycin (**9**).

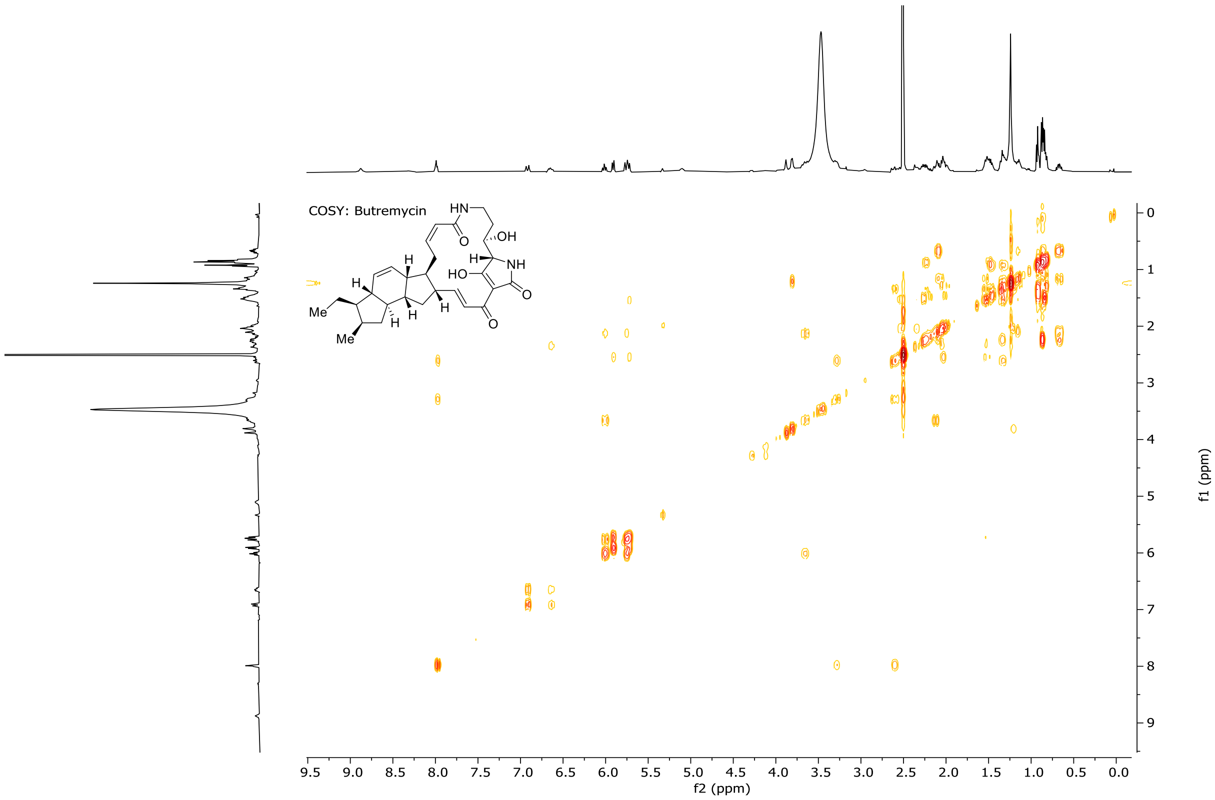

Figure S22. ^{1}H–^{1}H-COSY NMR of butremycin (**9**).

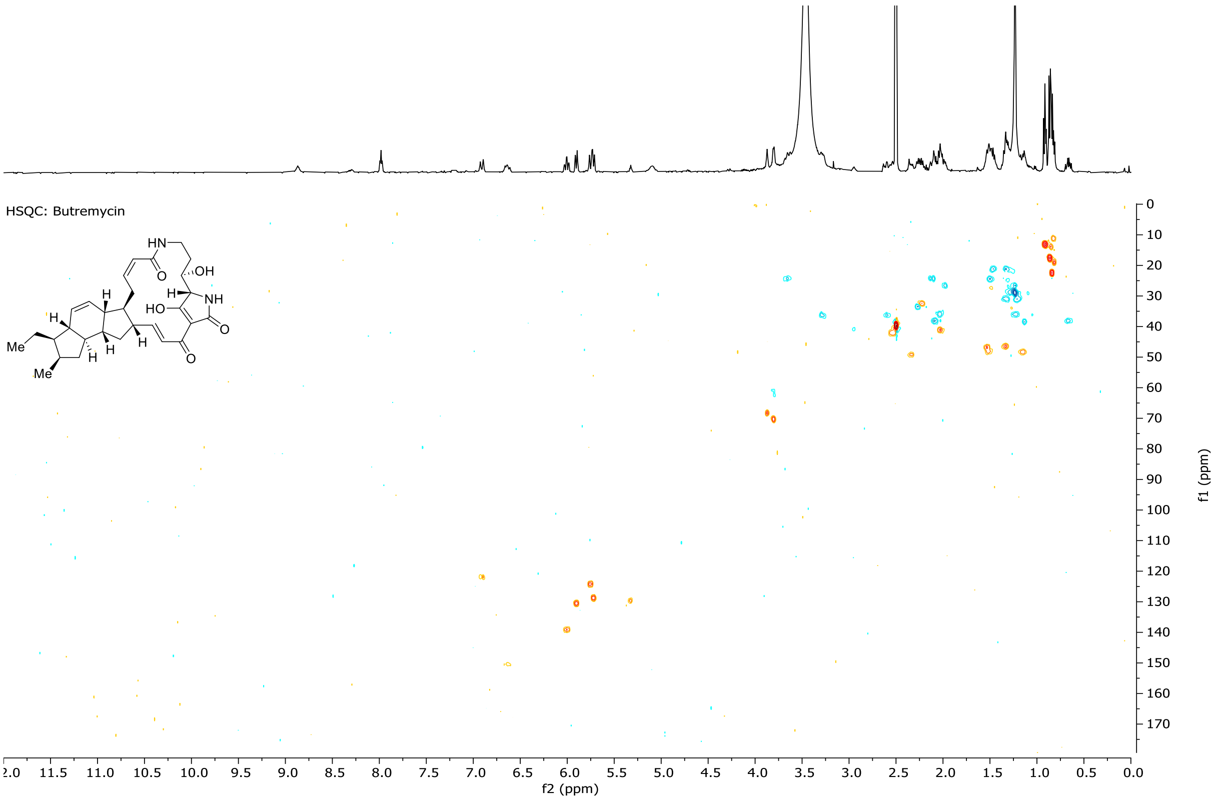

Figure S23. ^{1}H–^{13}C-HSQC NMR of butremycin (**9**).

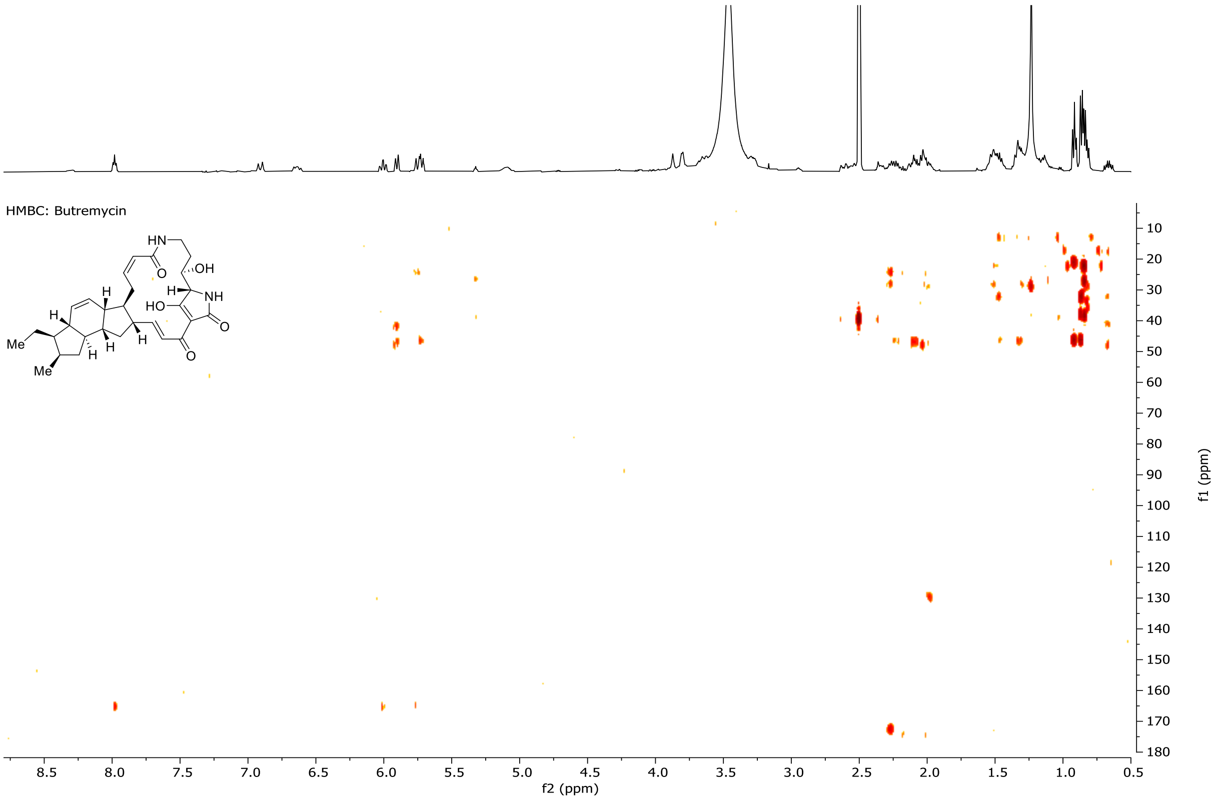

Figure S24. ^{1}H–^{13}C-HMBC NMR of butremycin (**9**).

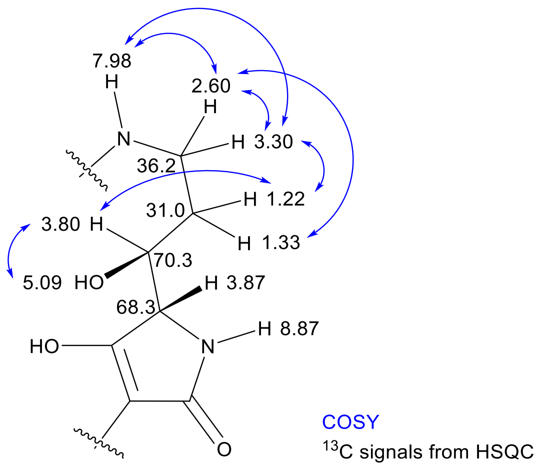

Figure S25. Structure elucidation of side chain of compound **9**. The section depicts the region with divergent NMR signals compared to **1**. Compound **9** was analyzed by 1D and 2D NMR and the position of the hydroxy group was localized at C-3. Crucial COSY interactions are indicated by blue double-arrows.

Table S4. ^{13}C NMR chemical shift values (C-2, C-3) of (2*S*,3*S**)-C-3–OH PoTeM derivatives (in DMSO-d_6).

Entry	Name	δ_{13C} [ppm] C-2	δ_{13C} [ppm] C-3	Ref.
1	Hydroxyikarugamycin A	68.7	70.9	(1)
2	Maltophilin	68.2	69.7	(2)
3	Maltophilin	68.6	70.1	(3)
4	Somamycin A	66.6	70.4	(4)
5	Somamycin B	66.7	70.2	
6	Somamycin C	66.6	70.4	
7	Somamycin D	66.9	70.4	
8	10-*epi*-HSAF	68.6	70.1	(5)
9	Umezawamide B	69.0	69.5	(6)
10	10-*epi*-Maltophilin	68.5	70.1	(7)
11	10-*epi*-Hydroxymaltophilin	68.6	70.1	
12	Clifednamide E	67.2	71.0	(8)
13	Hydroxycapsimycin	68.2	70.3	(9)
14	Brokamycin	68.3	70.4	

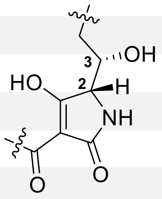

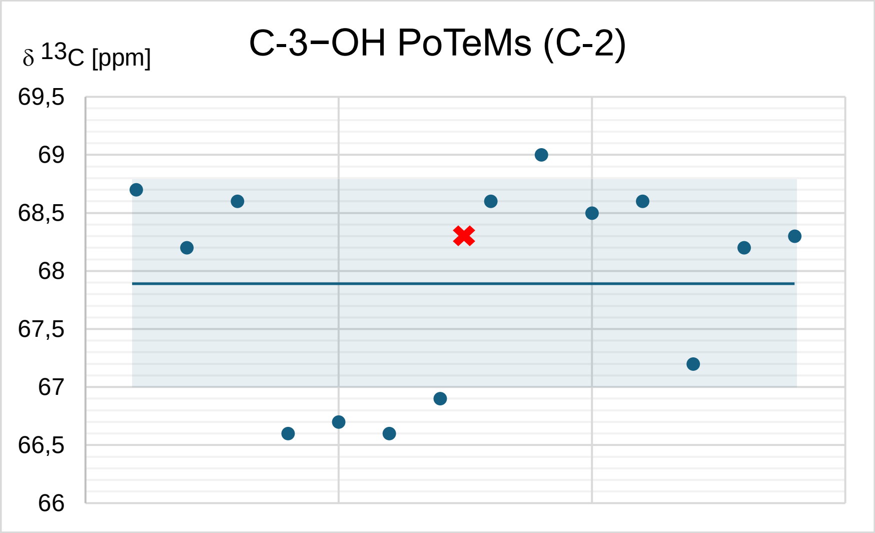

Figure S26. ^{13}C NMR chemical shift comparison (C-2) of C-3–OH PoTeMs (left to right: Table S4 entry 1 to 14; blue line and area: average and standard deviation (67.9 ± 0.9 ppm); red X labels observed shift of compound **9** (68.3 ppm) in our study).

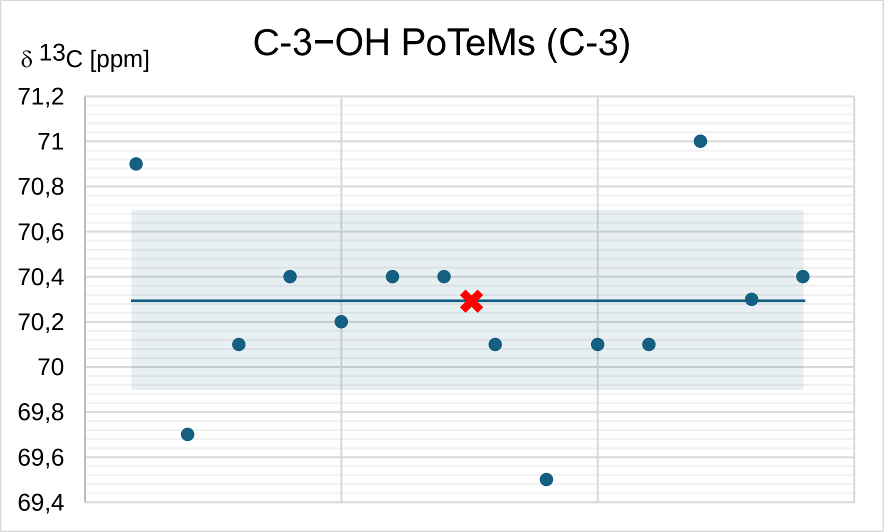

Figure S27. ^{13}C NMR chemical shift comparison (C-3) of C-3–OH PoTeMs (left to right: Table S4 entry 1 to 14; blue line and area: average and standard deviation (70.3 ± 0.4 ppm); red X labels observed shift of compound **9** (70.3 ppm) in our study).

Clifednamide A (10)

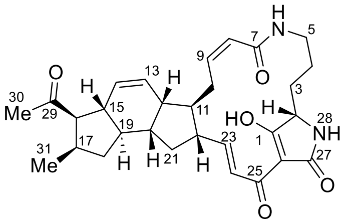

Table S5. ^{1}H, ^{13}C NMR (600 MHz, Pyridine-d_5) of clifednamide A (**10**).

	**δ$_C$**	**δ$_H$ (m, *J*)**	**COSY**
1	197.2	/	
2	62.5	4.12 (d, 5.2 Hz)	3
3	28.3	2.20–2.12 (m) 1H, 2.07–2.00 (m)	2, 4
4	22.0	1.81–1.72 (m) 1H, 1.58–1.49 (m)	3, 5
5	39.7	3.90–3.85 (m) 1H, 2.83–2.75 (m)	4, 6
6	/	8.86 (t, 5.6 Hz)	5
7	167.2	/	
8	125.7	6.25 (d, 11.4 Hz)	9, 10
9	140.1	5.97 (td, 11.0, 3.4 Hz)	8, 10
10	26.0	4.17 (ddd, 15.7, 10.3, 4.2 Hz), 2.62–2.53 (m)	8, 9, 11
11	49.3	1.52–1.45 (m)	10, 12, 22
12	43.2	2.47–2.42 (m)	11, 13, 14, 20
13	129.5	5.70 (dt, 9.8, 2.8 Hz)	12, 14, 15
14	130.6	5.86 (bd, 9.8 Hz)	12, 13
15	43.6	2.52–2.47 (m)	13, 16, 19
16	59.3	2.70 (dd, 12.1, 10.3 Hz)	15, 17
17	34.1	2.59–2.52 (m)	16, 18, 31
18	39.1	2.10–2.01 (m) 1H, 0.67 (td, 12.0, 7.7 Hz)	17, 19
19	48.1	1.15–1.07 (m)	15, 18, 20
20	41.7	2.02–1.96 (m)	12, 19, 21
21	37.1	2.03–1.96 (m) 1H, 1.13–1.06 (m)	20, 22
22	50.1	2.40–2.31 (m)	11, 21, 23
23	150.5*	6.93 (dd, 15.5, 10.3 Hz)	22, 24
24	124.5*	7.71 (d, 14.8 Hz)	23
25	ND	/	
26	ND	/	
27	ND	/	
28	/	9.66 (bs)	
29	209.6	/	
30	31.6	2.16 (s)	
31	19.4	0.85 (d, 7.2 Hz)	17

* ^{13}C shift taken from HSQC. ND: not detected.

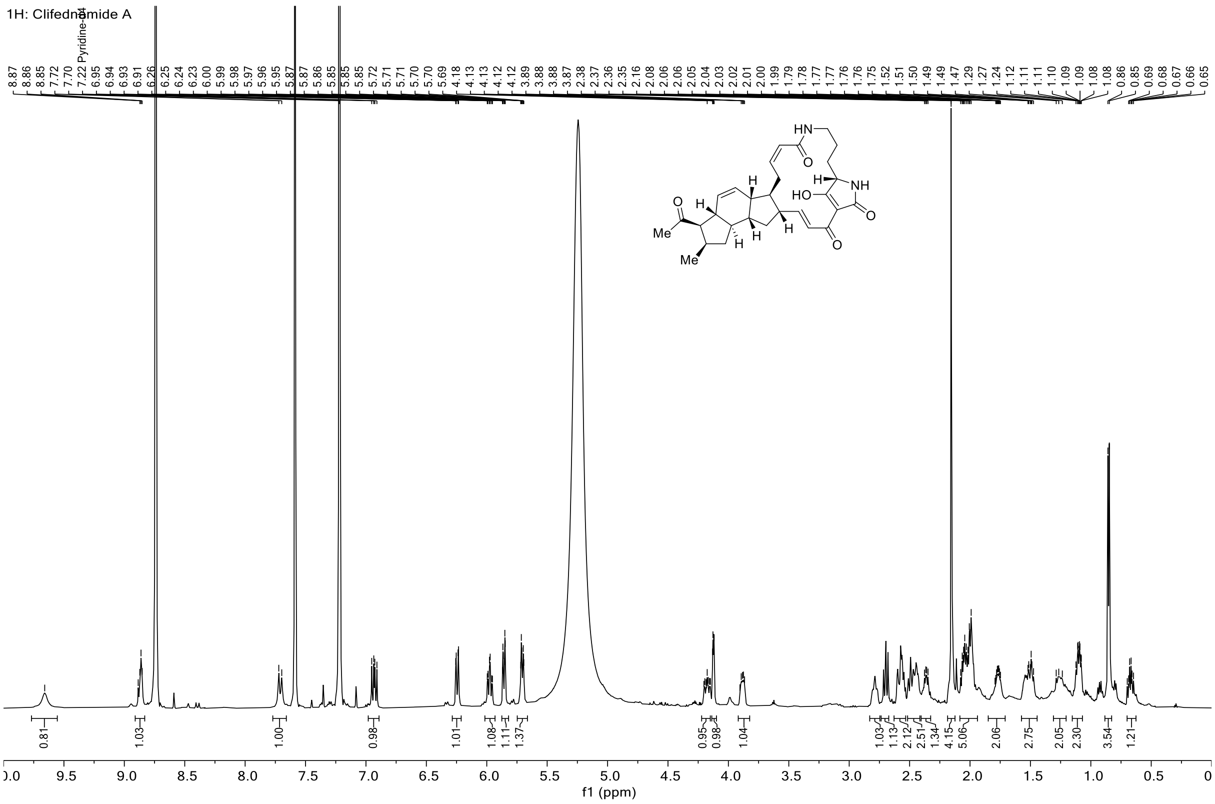

Figure S28. ^{1}H NMR of clifednamide A (**10**).

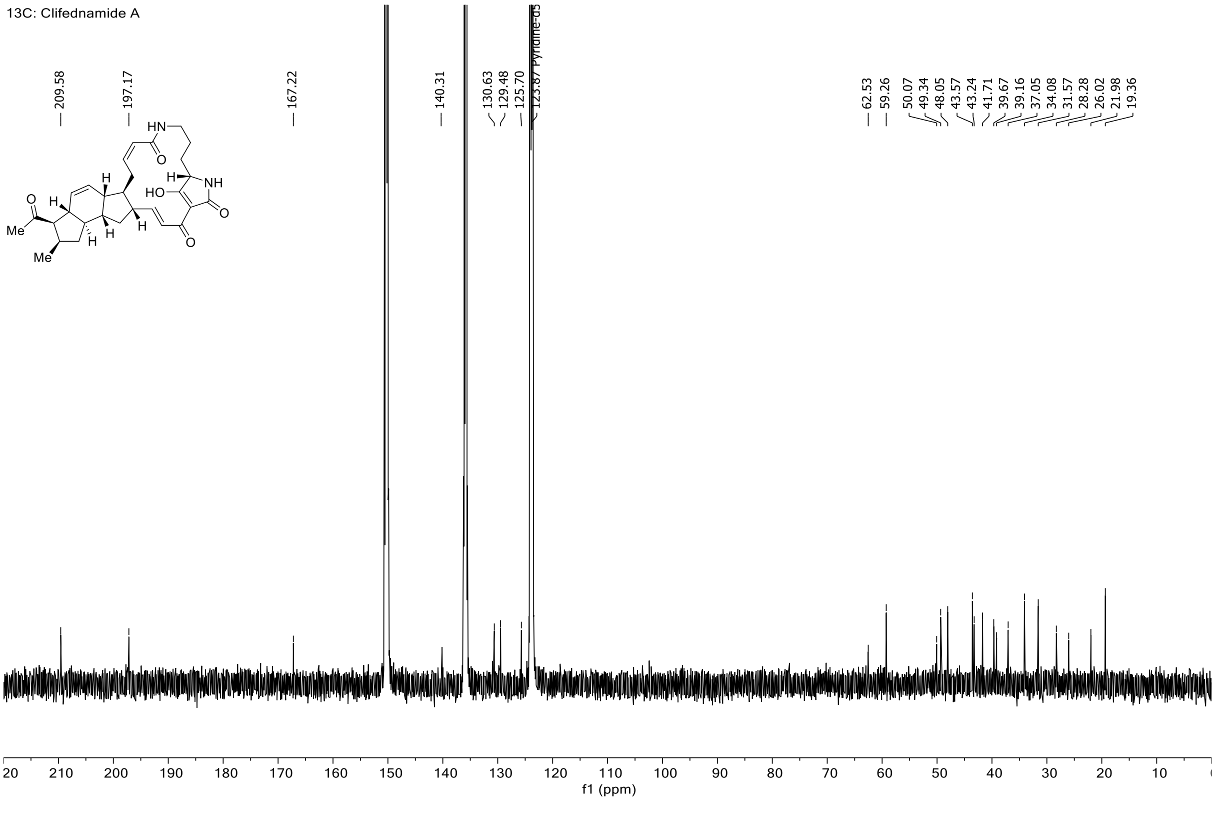

Figure S29. ^{13}C{^{1}H} NMR of clifednamide A (**10**).

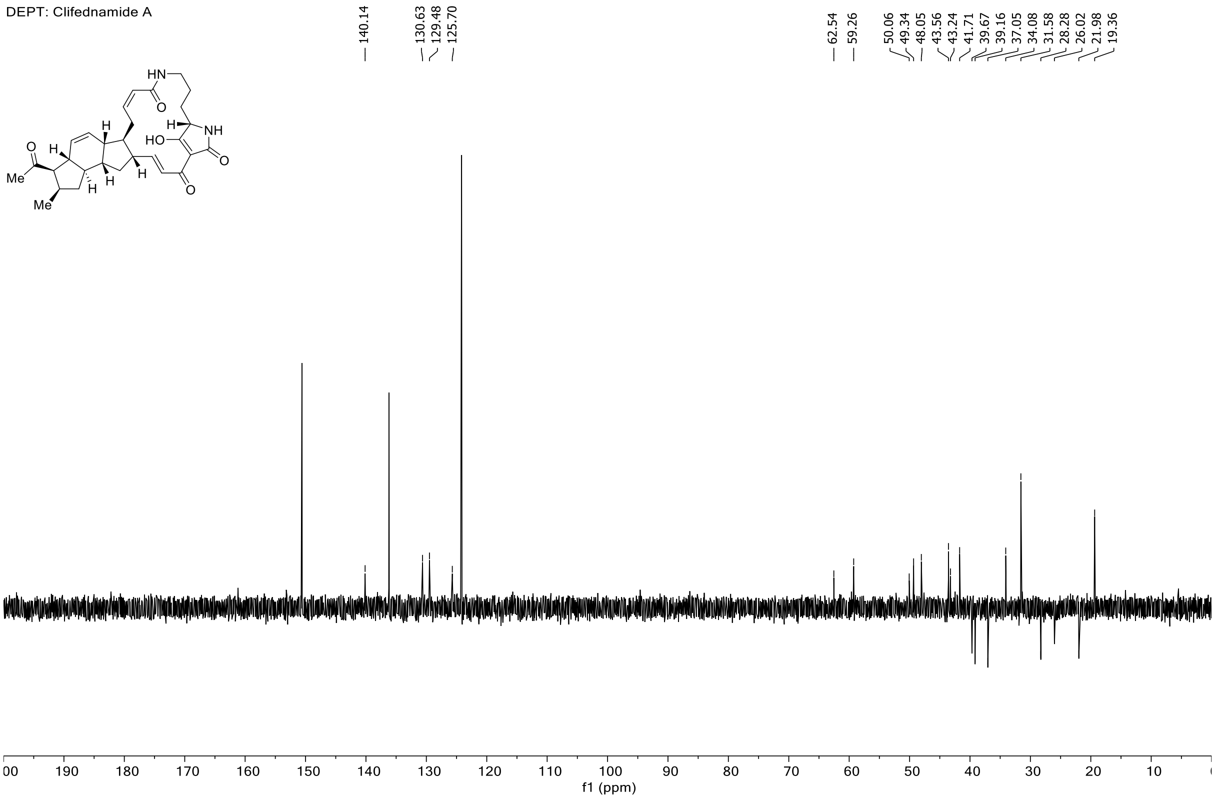

Figure S30. $^{13}C\{^{1}H\}$ DEPT NMR of clifednamide A (**10**).

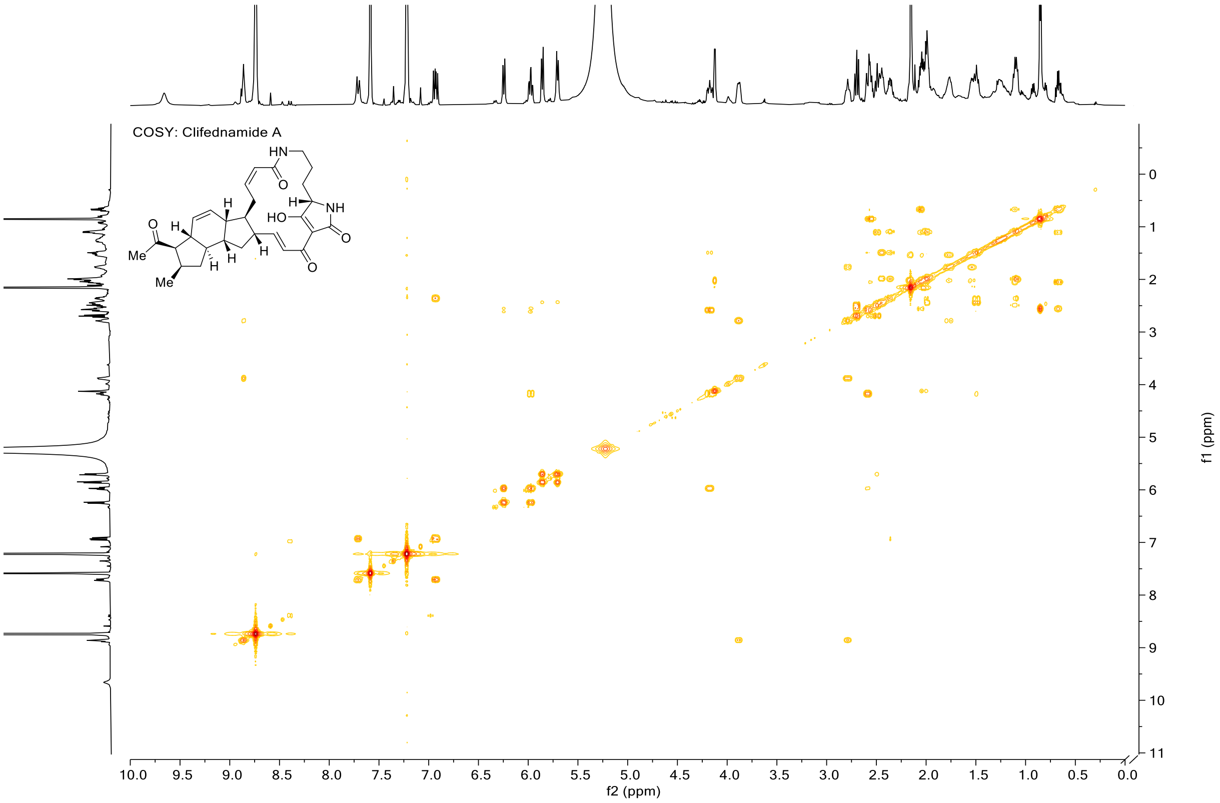

Figure S31. ^{1}H–^{1}H-COSY NMR of clifednamide A (**10**).

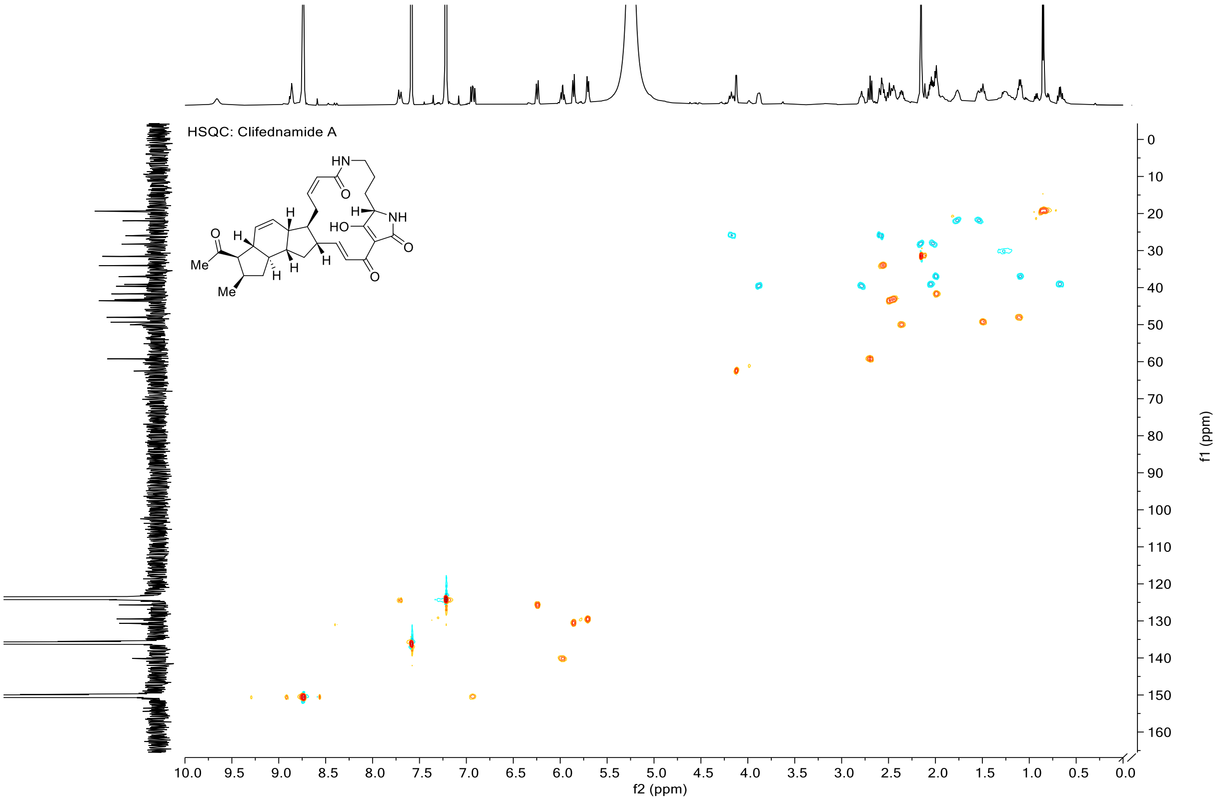

Figure S32. ^{1}H–^{13}C-HSQC NMR of clifednamide A (**10**).

Clifednamide C (11)

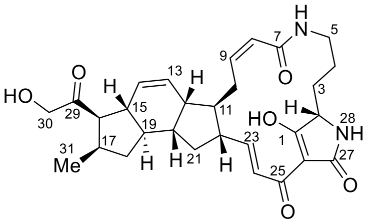

Table S6. ^{1}H, ^{13}C NMR (600 MHz, Pyridine-d_5) of clifednamide C (**11**).

	δ_C	δ_H (m, *J*)	COSY
1	197.2	/	
2	62.4	4.11 (d, 4.9 Hz)	3
3	28.3	2.20–2.14 (m), 2.08–1.98 (m)	2, 4
4	22.0	1.80–1.72 (m), 1.58–1.51 (m)	3, 5
5	39.6	3.91–3.85 (m), 2.82–2.76 (m)	4, 6
6	/	8.86 (t, 5.7 Hz)	5
7	167.2	/	
8	125.7	6.25 (d, 11.3 Hz)	9, 10
9	140.0	5.97 (td, 11.0, 3.4 Hz)	8, 10
10	26.0	4.18 (ddd, 15.8, 10.6, 4.4 Hz), 2.62–2.53 (m)	8, 9, 11
11	49.3	1.52–1.46 (m)	10, 22
12	43.2	2.47–2.41 (m)	13, 14, 20
13	129.6	5.68 (dt, 9.9, 2.8 Hz)	12, 14
14	130.3	5.79–5.76 (m)	12, 13
15	44.0	2.67–2.60 (m)	16, 19
16	54.3	2.96 (dd, 12.0, 10.3 Hz)	15
17	35.0	2.70–2.64 (m)	31
18	39.5	2.08–2.01 (m), 0.72 (td, 12.0, 7.7 Hz)	19
19	48.3	1.18–1.12 (m)	15, 18, 20
20	41.6	2.02–1.96 (m)	12, 19
21	37.1	2.02–1.96 (m), 1.13–1.07 (m)	22
22	50.0	2.36 (dd, 10.9, 6.5 Hz)	11, 21, 23
23	149.9*	6.92 (dd, 15.5, 10.1 Hz)	22, 24
24	125.0*	7.79–7.71 (m)	23
25	174.8**	/	
26	102.5	/	
27	177.5	/	
28	/	9.54 (bs)	
29	213.0	/	
30	70.6	4.63 (d, 18.7 Hz), 4.53 (d, 18.7 Hz)	
31	19.5	0.91 (d, 7.0 Hz)	17

* ^{13}C shift taken from HSQC. ** ^{13}C shift taken from HMBC.

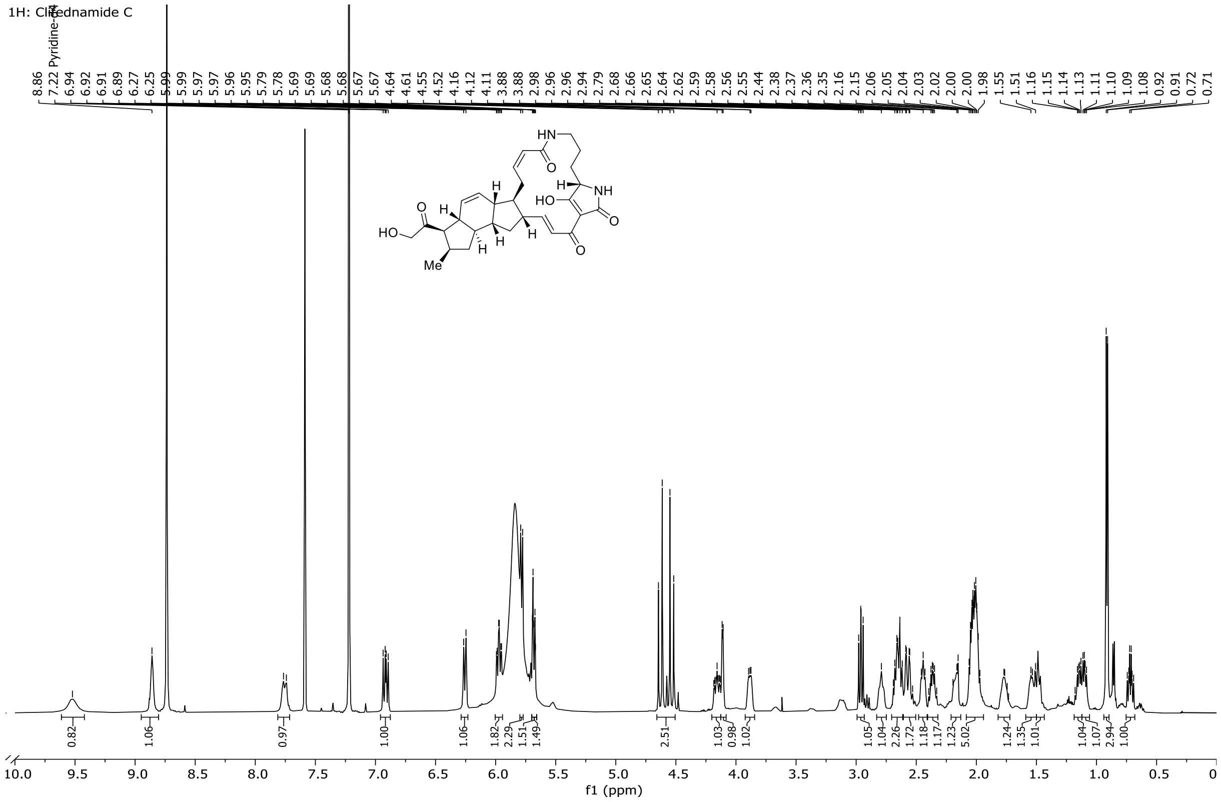

Figure S33. ^{1}H NMR of clifednamide C (**11**).

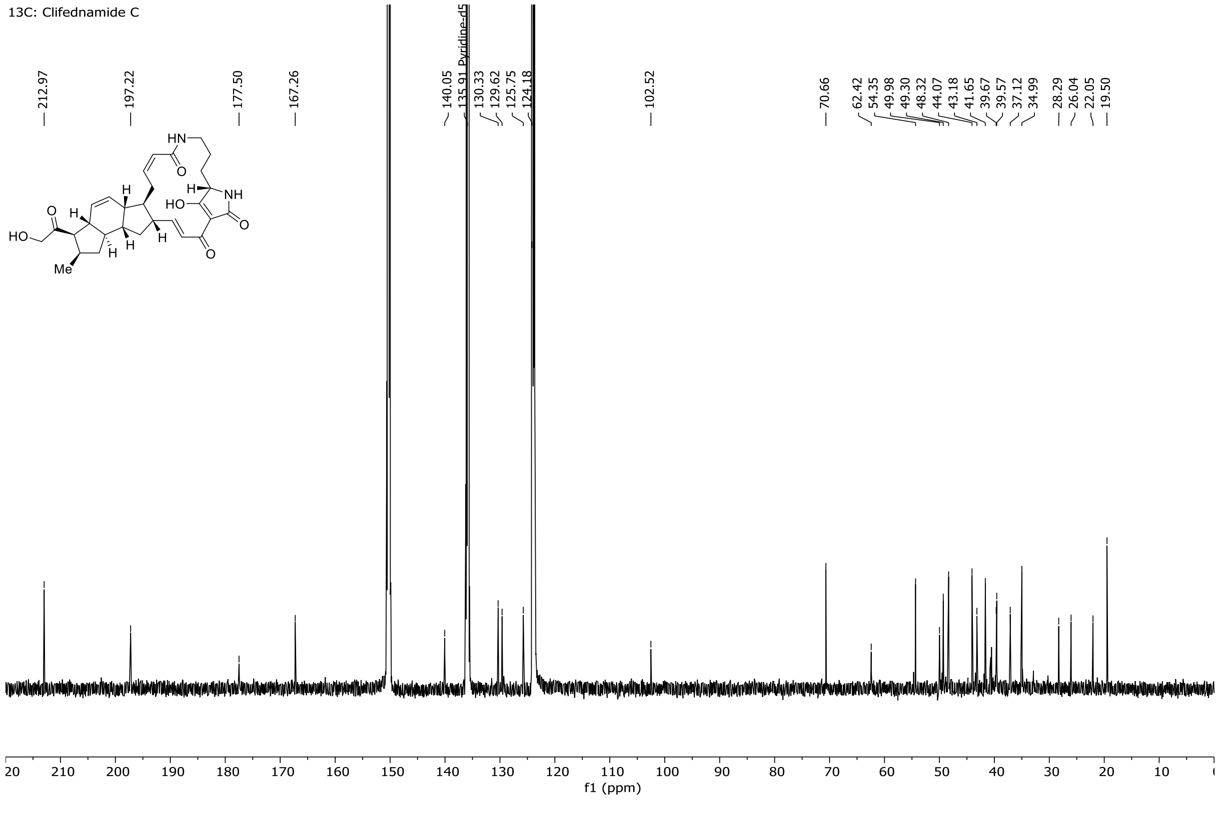

Figure S34. ^{13}C{^{1}H} NMR of clifednamide C (**11**).

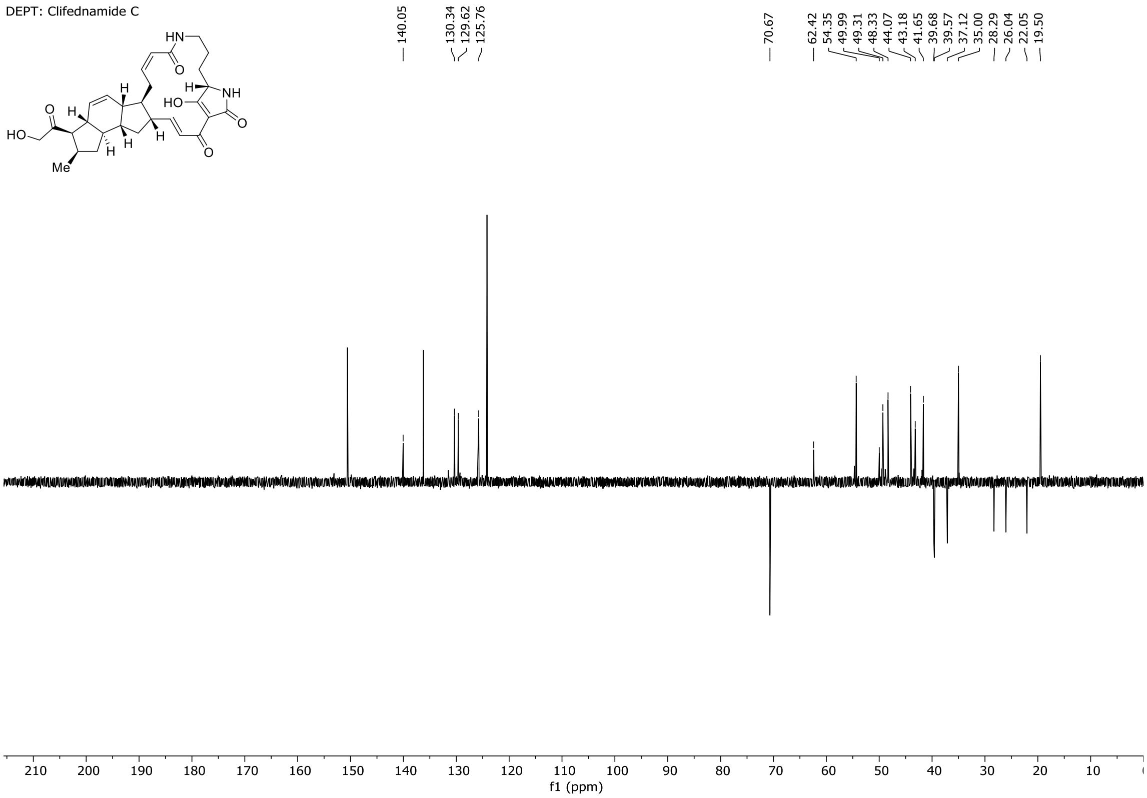

Figure S35. $^{13}C\{^{1}H\}$ DEPT NMR of clifednamide C (**11**).

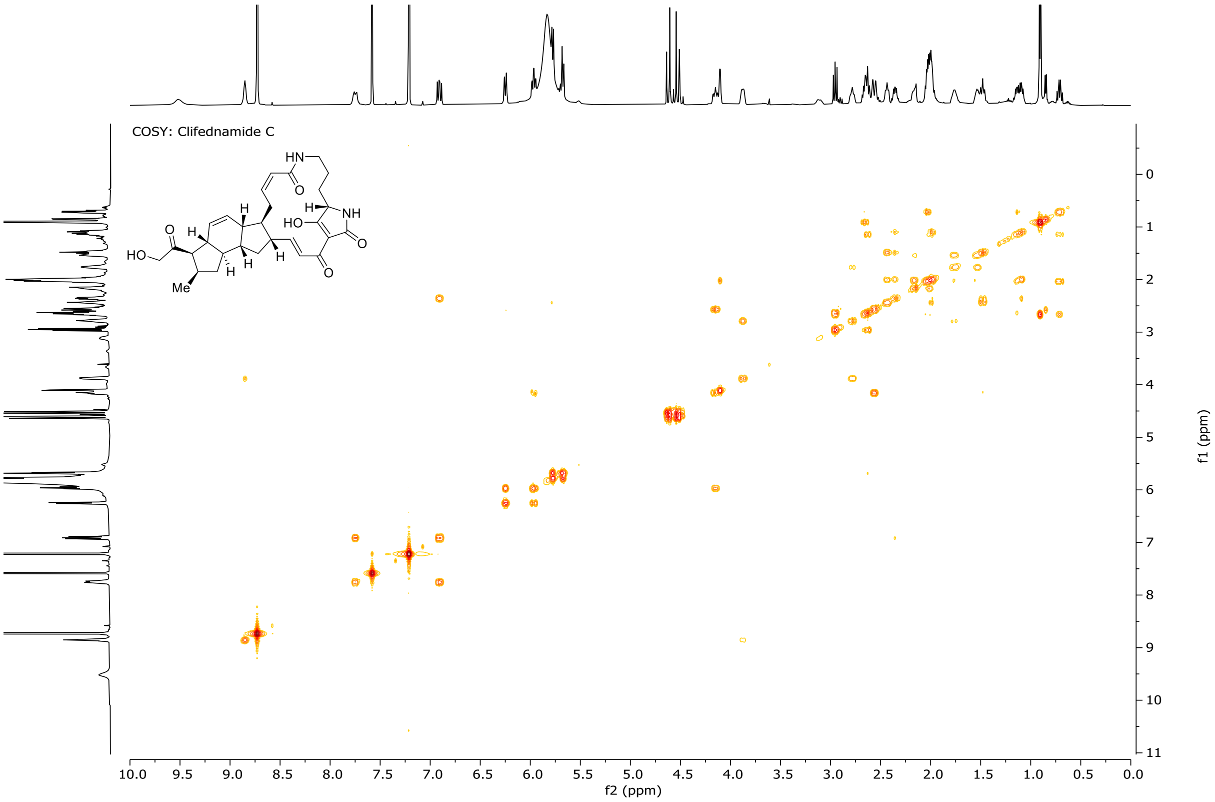

Figure S36. ^{1}H–^{1}H-COSY NMR of clifednamide C (**11**).

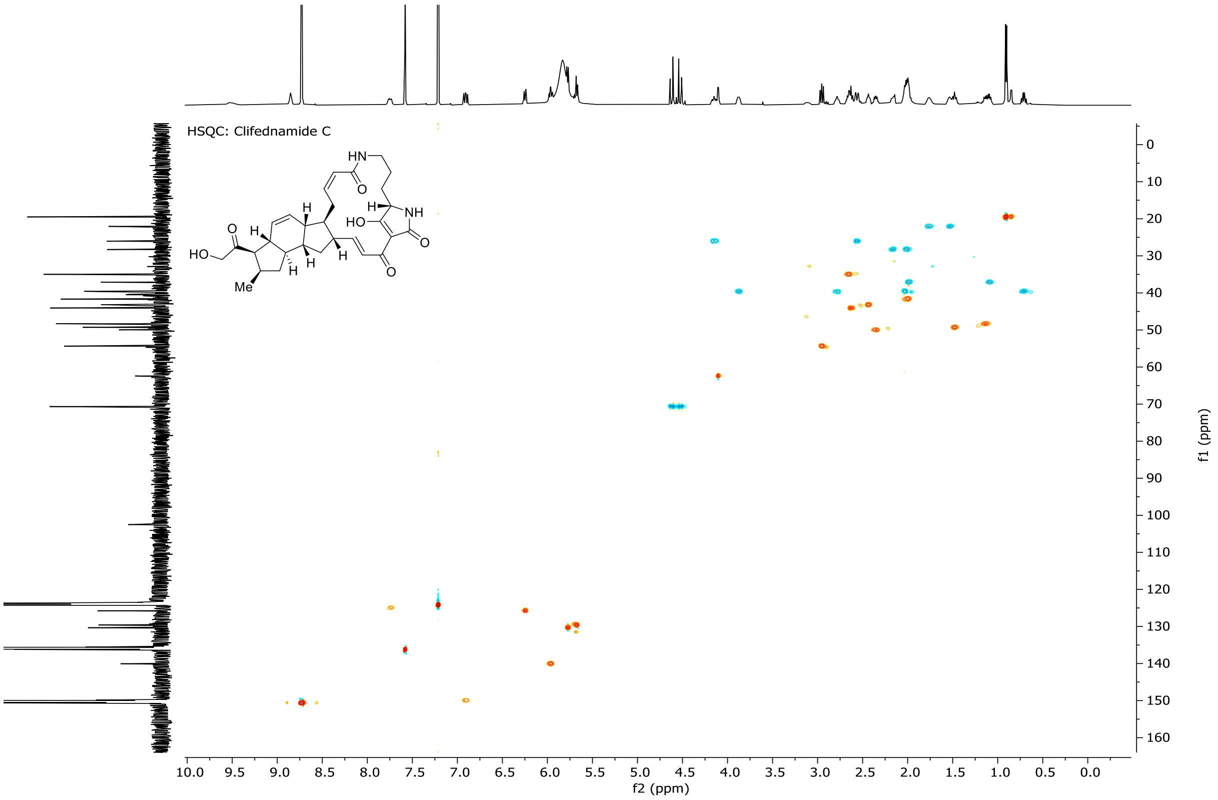

Figure S37. ^{1}H–^{13}C-HSQC NMR of clifednamide C (**11**).

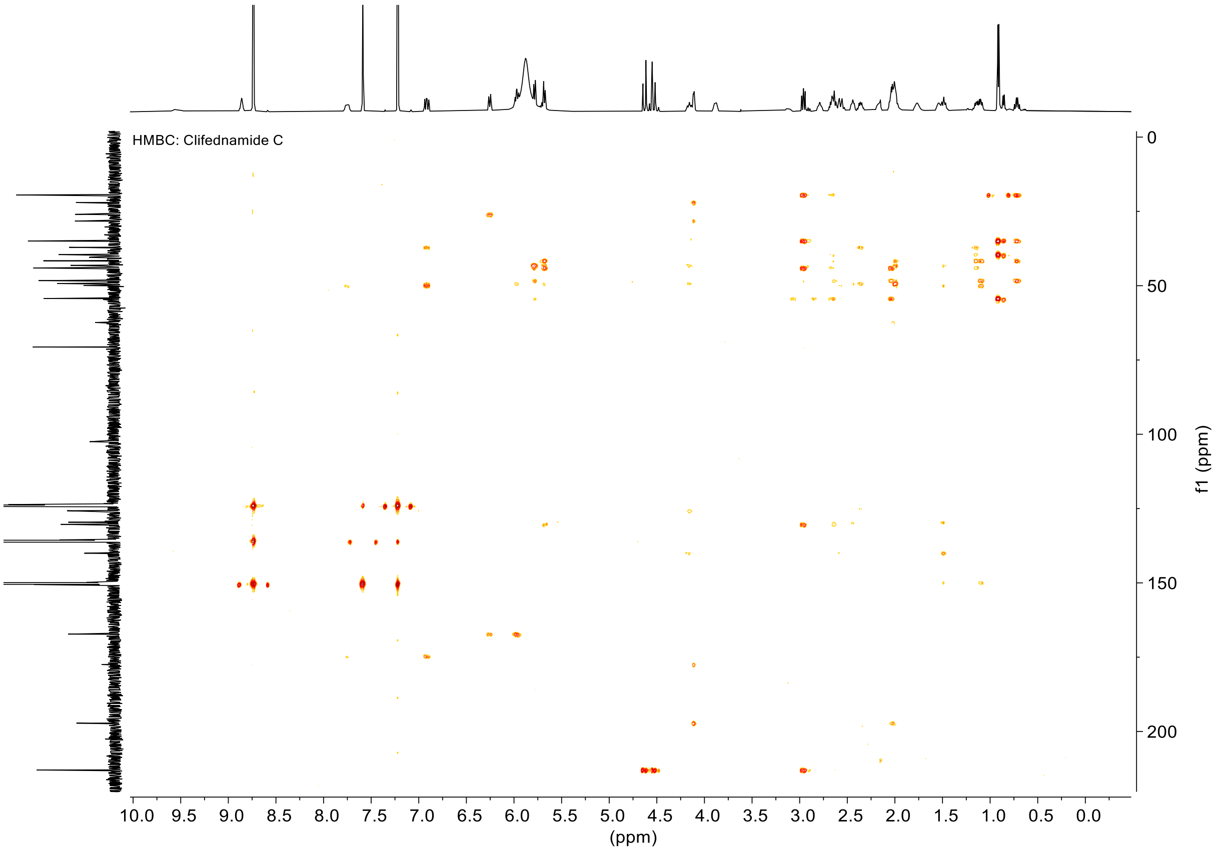

Figure S38. ^{1}H–^{13}C-HMBC NMR of clifednamide C (**11**).

6. Bacterial strains, primer, plasmids, and sequences

Strains

Table S7. Bacterial stains used in this study.

Strain	Usage	Source/Reference
E. coli DH5α	Cloning	NEB
E. coli ET12567 (pUZ8002)	Donor stain for conjugation	(10)
S. albus DSM40313	Heterologous PoTeM expression	DSMZ (11)
S. lividans TK24	Heterologous PoTeM expression	(12, 13)
S. coelicolor M1154	Heterologous PoTeM expression	(14)

Primers

Table S8. Primers used in this study.

Name	Sequence (5′ → 3′)	Function
ikaA-fwd	GCAGGTCGACTCTAGAGAGGCCTTCATACC TCGCCATCACC	*ikaA* amplification for pSET152_ermE*
ikaA-rev	CAACGGAGGTACGGAAGGATGTATTCATGG ATTCCATGCACCACCCTGC	*ika* amplification for pSET152_ermE*
ikaBC-fwd	CCGTCAAGATCGACCGCAGGCTACAGGGCGA CCAGGAC	*ikaBC* amplification for plug-and-play
ikaBC-rev	GTGATGGCGAGGTATGAAGGATGACGCCTT TCGTTCAGC	*ikaBC* amplification for plug-and-play
ikaD-fwd	GTACTCTAGACTACCAGGCGACCGGCAGT	*ikaD* amplification
ikaD-rev	GCTATCTAGAATGCCCGGACAGCAGGAACA	*ikaD* amplification
ptmD-fwd	GTACTCTAGACTACGCGGTGTGGGTCGG	*ptmD* amplification
ptmD-rev	GCTATCTAGAATGGAGATTCTCCGCATGGAAG	*ptmD* amplification
cftA-fwd	GCTTGGGCTGCAGGTCGACTTCACCAGGCG ACCGGGAG	*cftaA* amplification
cftA-rev	GAGCAACGGAGGTACGGACTATGTCGGATC AACACCCCCC	*cftaA* amplification
Lac-promoter	CTTCCGGCTCGTATGTTGT	sequencing primer
M13-fwd	GTAAAACGACGGCCAGT	sequencing primer
ikaA-screen	TTCAACTACCTGATGGGCGA	sequencing primer
ikaC-screen	AGCCATCTGGTGTCCACC	sequencing primer
ikaC-screen2	TGTCGGTGGACCCGTATCT	sequencing primer

Plasmids

The basic plasmid of the plug-and-play system only contained the iPKS/NRPS (*ikaA*) as producer of lysobacterene A (**5**) under the ermE* promoter. A second promoter (Table S7) was located downstream flanked by two unique restriction sites (StuI and XbaI) to enable a fast insertion of modifying enzymes. The construct for functional investigation of modifying enzymes on ikarugamycin additionally contained *ikaB* and *ikaC* upstream to the second promoter to enable production of ikarugamycin (**1**).

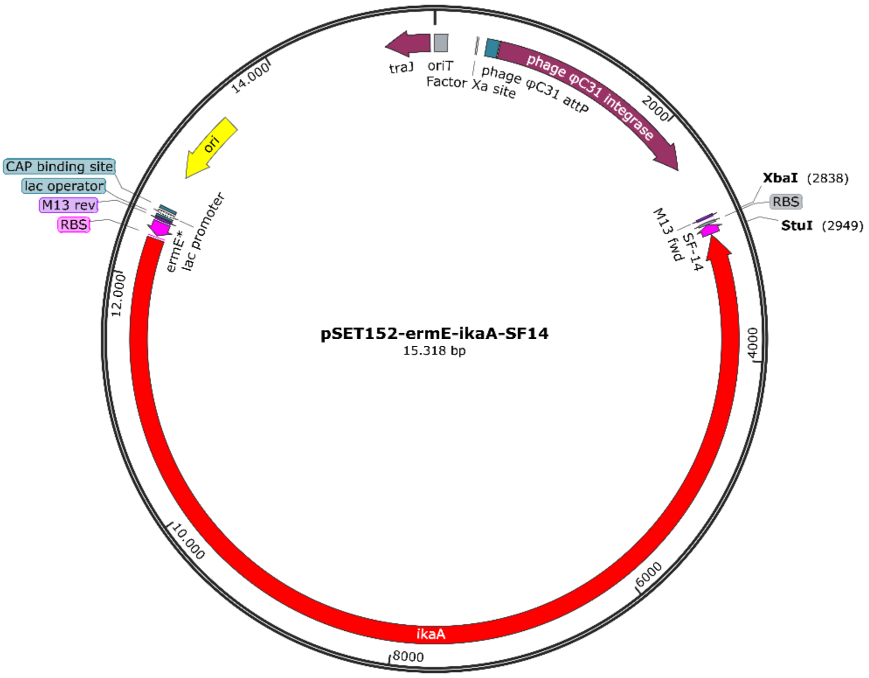

Figure S39. Basic expression construct of the plug-and-play system. The plasmid contained *ikaA* as reliable producer of lysobacterene A (**5**), the common precursor of all PoTeMs. Additionally, a second promoter was present to enable sufficient transcription of modifying genes.

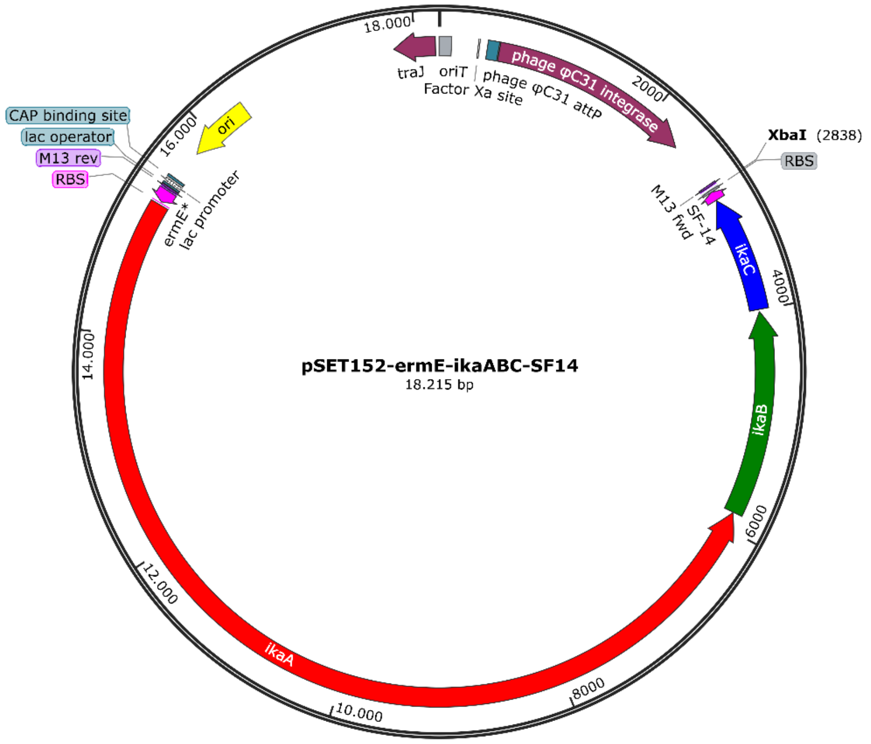

Figure S40. Plug-and-play system for the investigation of the influence of modifying enzymes on ikarugamycin (**1**). The plasmid contained *ikaABC* and produced ikarugamycin (**1**) in high yields. No modifying gene was included.

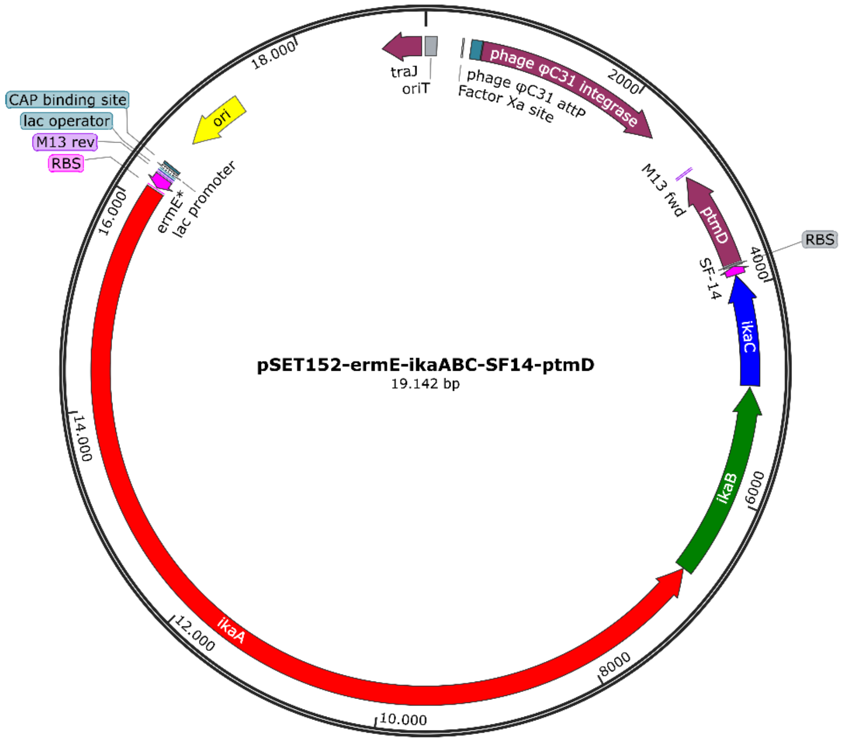

Figure S41. Exemplary expression construct for the investigation of the PoTeM hydroxylase PtmD. The vector contained *ikaABC* as well as *ptmD* under the control of the SF14P promoter.

Gene sequences

Gene sequence of the iPKS/NRPS *ikaA* from *Streptomyces* sp. Tü6239 (9375 bp; 336.7 kDa):

ATGGATTCCATGCACCACCCTGCCCCCGTCCCCGTACCCGAAGTCCCCGCGCCCGTCCCGTCCCAGGACGACGCG
TTCGCCATCGTCGGCATCGGCTGCCGGCTGCCCGGCGGCGCCAGCGACTACCGGACCTTCTGGCGCAACCTCCTC
GACGGCAAGGACTGCATCACCGACACCCCCGCCGACCGCTACGACACCCGCACCCTGGGCAGCGGCGACAAGGCC
AAGCCCGGCCGGCTGGTCGGCGGACGCGGTGGATACATCGACGGCTTCGACGAGTTCGACCCCGCCTTCTTCGGC
ATCAGCCCGCGCGAGGCCGAGCACATGGACCCCCAGCAGCGGAAGCTCCTGGAGGTCGCCTGGGAGGCGCTGGAG
GACGGCGGCCTCAAGCCCGCCGAGCTGGCCGGCAGCGATGTCGGGGTGTACGTCGGGGCGTTCACCCTCGACTAC
AAGATCCTGCAGTTCGCCGACCTCGGCTTCGAGACCCTGGCCGCGCACACCGCCACCGGCACCATGATGACGATG
GTGTCCAACCGGATCTCGTACTGCTTCGACTTCCGCGGACCCTCGGTCTCCGTCGACACCGCGTGCAGCGGCTCC
CTGGTCGCCGTCCACCTCGCCTGCCAGAGCCTGCGCCGCGGCGAGACCTCCGTCGCCCTGGCCGGCGGCACCCTG
CTGCACATGGCGCCGCAGTACACCATCGCCGAGACCAAGGGCGGGTTCCTCTCCCCCGACGGCCGCTCCCGCGCC
CTGGACGCCTCCGCCAACGGCTACGTGCGCGCCGAGGGCGTCGGCATGGTCGCCATCAAGCGCCTCGCGGACGCG
CAGCGCGACGGCGATCCCATCCACGCCGTCATCATCGGCAGCGGCGTCAACCAGGACGGCCGCACCAACGGCATC
ACCGTGCCCAACCCCGACGCGCAGGTCGCCCTGATCGAGCGGGTCTGCGCCGCCGCCGGCGTCACCCCCGGCAGC
CTCCAGTACGTCGAGGCGCACGGCACCTCCACCCCCGTCGGCGACCCGCTGGAGGCCAACGCCCTCGGCCGCGCG
CTCTCCATCGGCCGCGAGCCGGGCGCCCGGACGTACGTCGGCTCGGTCAAGACCAACATCGGGCACACCGAGTCC
GCCGCCGGCATCGCCGGGCTGATCAAGACGGTGCTCAGCCTCAAGCACAAGGTCATCCCGCCGCACATCAACCTG
GAGAAGCTCAACCCGCAGATCGACGAGGCGTCCCTGCCGTACGAGATCCCCCGCGAGCCCACCCCCTGGCCCGAG
CACAGCGGGCCGGCCCGGGCCGGCGTCAACTCCTTCGGCTTCGGCGGGACCAACGCCCACGTCCTGCTCCAGGAG
GCACCGCCGACCGTCGGGGAGCCCGCGCCACCGGCCACCGACGGGTACTCCGTGCTGCCGCTCAGCGCCCGCGAC
CCCGAAGCCTTTCCCGCCATCGCCACCGGCCTGCGCGAACGGCTCGCCGAGGGACTGCCGGTGGGCGACGCCGCC
TACACCCTCGCCCACCGGCGGCAGCATCTGGAGCAGCGGCTGTCCGTCGTGTACGACTCCCCCGAGGCCCTCGAC
GAGGTGCTCGGCGCCGTCGCCCGCGGCGAGAGCCACCCGCGTGCCGTCGCCGGCACCCAGCGGGAGGGCCTGGAC
CGCAGGCTGGTGTGGGTGTTCACCGGCATGGGCCCGCAGTGGTGGGCCATGGGCCGCCAGTTGTACGCGAGCGAG
CCCGTCTACCGGGAGGTCATCGACCGCTGCGACCAGGAGATCGCCGCGCTCACCGGCTGGTCCCTCACCCAGGAG
CTGAACGCCGACGAGGCCGACTCCCGGATGAGCGAGACCTGGCTCGCCCAGCCCGCCAACTTCGCCGTCCAGATC
GCCCTGGCCGCCCTGTGGCGCAGCAAGGGGATCCAGCCCGACGCCGTCACCGGGCACAGCACCGGTGAGGTCGCC
GCGTTCTACGAGGCCGGGGTGTACACCCTCCCCGAGGCCGTGAAGATCGTGGTGCACCGCAGCCGGCTCCAGCAG
AAGCTCATCGGCACCGGCTCCATGCTCGCCGTCAGCCTCACCGAGGCGGAGGCCGCCCGCCGGGTGCGCCCGCAC
GGCGACCGGGTCTCCATCGCCGCCGTCAACAGCCCCACCTCCATCACCCTGGCCGGGGACACCGAGGCGCTGGAG
GTGATCGCCGCCGAGCTGGGCGCCGAGGACATCTTCGCCCGCTTCCTGGAGGTCGGCGTCCCGTACCACAGCCCC
CGCATGGAGCTGATCAAGGACGAGCTGCTGACCTCGCTCGCCGATCTCAAGCCGCAGCAGGCGAAGTTGCCGCTG
TACCTCACCGCGCTGCCGGGCACCGTCGCGCAGGGCACGGAGCTGGACGCCGACTACTGGTGGCGCAATGTGCGC
GAGGCCGTGCACTTCCGGGCCGCCGTGGACCGGCTGCTGGACGACGGCTACGGCGTCTTCCTGGAGATCGGCCCG
CACCCCGTGCTCGCCCACTCCCTGCGCGAGTGCTGCGAGGCCCGCGACGCGCACAGCGTCACCCTGGCCTCCATC
CGCCGCAAGGCGGACGAGCGCGAACGCCTCACCCTGTCGCTCGCCGCGCTGCACAGCCTCGGCTTCGCCGTGGAC
TGGCACGCCCTGCACCCCGCCGGGCGGCCGGCCGAACTGCCGCGCTACCCGTTCCGGCGCGACCGGTACTGGGTC
GAGCCGGCCCCGGTCGCGCAGATCCGGCTCGGCCACCGCGACCACCCGCTGCTGGGCCGCCGCACCGCGAGCGCC
GAGCCGGTGTGGGAGGTGAAGCTGGACGCGGAGGCCGCCCCGTACCTGGAGGACCACCGCATCCAGGGCACCGTG
CTGTTCCCGGCCGCCGGCTATCTGGAGATGGCCGCGCAGGCCATGCGGGCGCTGACCGGTGATGAGCACAGCACC
GCCGCGCTGGCCGGCATCGAGCTGCGCAAGGCGCTGTTCCTGCCGGACGGCGAGCCGCAGACGGTGCAGCTGTCC
TTCTCCTCCGACGCCGCCGCGTTCTCCATCGCCACCGTGGGCGCCGCCGGCGCCGAGCCGACCGTGCACGCCACC
GGTACGGTACGGGCCGCCCAGCGCCGCCGGCTGACCGCGCCGCTGGACACCGTCGCCGTCCGGGCCCGCGCCGCC
CGCCACCTGAGCGGCCCCGACTGCTACGCCGAACTGGCCGCGCTCGGCTACCACTACGGCCCCGCCTTCCAGGGC
ATCGAGGAGGTGTGGATCGGCGAGGGCGAGGCCCTGGCCCGGATCCGTCCGCCGCAGGGGCTCACCCCGGACGCG
GCGGCGCACCACATGCATCCGGTGCTGCTCGACTCCTGCTTCCAGTCGCTGCTGACCCCGCAGCTGCTCACCGCG
CCCGCCGGGCCCGGGGGCACCGGCATCCGGCTGCCGCTGTCCATCGCCGAGGTACGGCTGGACCCGGTCGGCGAC
CGCGAACTGTGGGTGCACGCCACCGTCACCGGCGACGACGAGGACGAACTCACCGGTGACATCGCCGTGTACGAC
GGCGCCGACGGTACGCCGCTGGGCCGCGTCGCCGGCTTCCGCGCCGCCGATGTGGAGAAGGCCGCCACCACCGTG
GGGCTGTCCACCATCGACAGCTGGCTCACCGAACCGAGCTGGGTGCCGTGCCCGCTGCCCGAGGCGGCGTCCGCC
GCGCCGGCGGCCGGGCGGCACGTACTGTTCGCCGACGCGGGCGGGGTCGCGCAGCGGCTGGCCGCGCTGATCGGC
GAGGCCGGCGGGGAGGCCCATCTGGTCCGGCCCGGTGCCGCGTACGGCCTGGACCGCACGGCGAGGACCGCCACC
GTCGTCCCCGGATCCGCGGATGACCTGCGGCGGTTGCTCACCGATCTCGGGCAGGTGGACGGCGTCGTCCACCTG
TGGAACCTGGACCGGCCGGCGCTGGCCGACGCCCCGCGCGGACGGTTCGCGGACATCGCCTCCACCGGCGCGTAC
GCCCTGATCGCCCTCACTCAGGCCCTGCTCGCCGACCCGGAGCGGCACGGCGGCACCCCGGTGCACATCGTCACC
AGAGCCGCCCAGTGCGTGGTCCCCGGTGAGCCGGTGGAGCCGCTGGGCGCGCCCGCCTGGGGCATCGGCCGGGTG
CTGTGGCAGCAGGAACTGGCCGGGCGCGGCGGCAAGCTGATCGACCTGGCGGCCGACGGCGGCGTCGAGGAGGAC

GCGTACGCGCTGCTGCGCGAGCTGGCCGACCCCACCGGCGCGGCCGAGCGCGAGGACGAGATCGCGCTGCGCGCC
GGGGAGCGGCACACCAGCCGGCTGGTGGCCGCCGAGGGGCTGAGCAGGCCGCTGCCCCTGCGGCTGCGCCCGGAC
GGCAGCTATCTGGTGACCGGCGCGTTCGGCGCGCTCGGCAGGCTGCTGTGCCGCACGCTGGTCAGGCGCGGGGCG
CGGCGGCTGATCCTGGTGGGCCGCACCCGGCTGCCGGAGCGCGAGCGCTGGGCCGACCAGGACCCGAACTCGCCG
GCCGGGCGGCACGTGGCCTTCCTCAAGGAGCTGGAGGCGCTGGGCGCGCAGCCGATTCTCGCGCCGCTGGACATC
ACCGACGAGGACGCGCTGGCCGGCTGGCTCGCCGGGTACCGGCGCGCCCAGGGGCCGCCGATCCGCGGGGTGTTC
CATCTGGCGGGGCAGGTGCGCGACACCCTGGTGCCGGAGATGGACCGGGAGGTGTTCGACGCCGTCCACGACCCG
AAGGTGGTGGGCGCGGCGCTGCTGCACCGGCAGCTGAGCGGCGAACCGCTGGAGCACTTCGTGCTGTTCGCCTCG
GTCGCGGCCTGGCTGACGACGGCCGGACAGACCAACTACGCGGCGGGGAACGCCTTCCTGGACGCGCTGGCGCAC
CACCGCCGCGCGCAGGGGCTGCCGGCGCTGGCGCTGGACTGGGGCCCGTGGGCCACCGGCATGATCGAGGAACTG
GGCCTGATCGACCACTACCGCAACAGCCGGGGCATGTCCTCGCTGGCGCCCGAGGCGGGCATGGCGGTGCTGGAG
CGGGTCATCGGGCAGGACCGGGCACAGCTGCTGGTGGCCACGGTCGTGGACTGGCCGGTGTTCATGTCCTGGTAC
GCGGCGCCGCCGCGGCTGGTCACGGAGCTGGCGGCCACCGCCCAGGGACCGGGGTCCGAGGGCGACGGCAGTTTC
CTGGACGCGTTCCGGGAGGCCACCGCGGACAAGCGGCGGCTGCTGCTGACCGAGCGGTTCACGACGCTGGTGGCG
GGTGTGCTGCGGGTGCGGGCCGAGCAGGTGGATCCGGCGGTCAGCCTGAATCTGCTGGGGCTCGACTCGCTGCTG
GCGATGGAGCTGCGAGCGCGGGTGGTGGCCGAGGTGGGCATCGCGCTGCCGGTGGTGGCGCTGCTGTCCAGCGCG
CCGGCCGGGGACCTGATCACCCAGCTGCACGAGGGCCTGGAGGAGTTGCTGGCCGAGGAGGGCAGCGGCGCCGCG
GTGACGGCGGTGGAGCGCTTCGAGGACGAGGCCGAGTTCCCGCTGACGCAGAACCAGAAGGCGCTGTGGTTCCTG
AAGCAGCTGAACCCGGACGGCTTCGCGTACAACATCGGCGGCGCCGTCGAGGTGCGGGTCGAGCTGGACCCGGAC
CTGATGTTCGAGGCGTTTCGCCGGCTGCTGGCCCGGCATCCCGTGCTGCGGGCGAACTTCCTGCTGGTGGAGGGG
CAGGCGGTGCAGCGGATCTCCCCGGAGATCAAGGAGGACATCGCGCTCTTCGACGTCGAGGACCGCGCGTGGGAC
GACATCTACCGGATGATCATCGAGGAGTACCGCAAGCCGTACGACCTGGCGACCGATCCGCTGATCCGGTTCCGC
CTCTTCCGGCGCGGCCCGGACCGCTGGGTCATCACCAAGGCCGTCCACCACATCATCTCGGACGCCATCTCCACC
TTCACCTTCATCGAGGAACTGCTGTCCCTGTACGAGGGGCTGCGGCAGGGCCACGACGTCGAACTGCCGCCGGTG
TCCGCCCGCTATCTGGACTTCCTCAACTGGCAGAACGCGTTCCTGGCCGGCCGCGAGGCGCAGAAGATGCTCGCG
TACTGGCGGGGGCAGCTGCCGGACGAGGTGCCGGTGCTGGCGCTGCCCACCGACAAGCCGCGCCCGGCGGTGCTC
ACCCACAACGGGGCGTCCGAGTTCTTCGCCCTGGACGCGGAGTTGAGCGCCCGGGTGCACGCGCTGGCGCGGGAG
CACAACGTCACCGTCTTCATGGTGCTGCTGAGCGCGTACTACCTGCTGCTGCACCGCTATGCGGGGCAGGACGAC
ATCATCGTCGGCTCCCCCGTCACCGGCCGCACCCAGGAGGAGTTCGGCGCCGTCTACGGGTACTTCGTGAACCCG
CTGCCGCTGCACGCCTCGCTGGCCGGTGACCCCACGGTCGCCGAGCTGCTGGACCAGGTGCGCACCACGGTGCTG
GGCGGCCTGGACCACCAGGAGTACCCGTTCACGCTGCTGGTGGAGCAGCTGGGGCTGGCCCACGACCCGAGCCGG
TCGGCGGTCTTCCAGGCGATGTTCATCCTGCTGCACCACAAGGTGGCCACCGAGAAGTACGGCTACAAGCTGGAG
TACATCGAGCTGCCCGAGGAGGAGGGCCAGTTCGACCTGACGCTGTCCGCGTACGAGGAGGAGGCGGACGGGCGG
TTCCACTGCGTCTTCAAGTACAACACCGACCTCTTCGAGGCGGAGACGATCCGGCGGCTCGCCGGGCACTACACG
CAGCTCCTGGAGTCGCTGACCGCGGCGCCCGCCGACGCCGCCACCGGTGGACTGCGGATGCTGTCGGGCGGCGAG
CGGGAGCGGATCCTCACCGAGTGGAGCGGGGCCGGGCAGGGCGCGCAGGACGCGCCGGTGCCGGTGCACCGGCTG
ATCGCCGAGGCGGCGCACCGTACCCCGCAGGCGATCGCGGTGGCCGCGCCCGCCGAGAGCGGGGAGACCCGGCGG
CTGACGTACGGCGAACTGGAGGAGCGCGCCGGCGAACTGGCCGGGCGGCTGCGGGCGCGCGGCGTGCGCGAGGGC
ACCGTCGTCGCGCTGTGCCTGGAGAAGTCGCCCGAGCTGATCACCGCCCTGCTGGCGGTCCTCAAGGCGGGCGGC
GCCTATCTGCCGCTGGACCCGGACTATCCGGCCGACCGGCTCGCGTACATGGTGCGCAACGCCGGGGCCACGCTG
GTGATCGGCGGGACGGGCGGCGCGGCCGAGGGGCTGCCGGGCACCGTGGTCACCCTGGAGGAACTGCTCGCGGGC
GAGGCCGGCGAAGCGGGGCCGGACGCCGAGCCGGGGCCCGACTCCCCCGCCTACGTCATCTACACCTCGGGCTCC
ACCGGGCGCCCCAAGGCGGTCGCGGTCAGCCACCGCAATCTGGCCTCGGTGTACGCCGGATGGCGCGACGCCTAC
CGCCTGGAGGAGGGCGGCATCCGGGTCCATCTCCAGATGGCCAGCCCCTCCTTCGACGTCTTCACCGGCGACCTG
ACCCGAGCCCTGTGCTCGGGCGGCACGCTGGTGCTGGTCGGCCGGGAGCTGCTGTTCAACACCGCCCGGCTGTAC
GAGACGATGCGCGCCGAACGGGTGGACTGCGGCGAGTTCGTGCCCGCCGTGGTGCGCACCCTGGTGCGGCACTGC
GAGGACACCGGCGCCCGGCTGGACTTCCTGCGGCTGCTGATCGTGGGCTCGGACTCCTGGAAGGCCGAGGAGTAC
GAGCGGCTGCGCGCGCTGGGCGCACAGCGCCTGGTGAACTCGTACGGGCTCACCGAGGCCACCATCGACAGCGCC
TGGTTCGAGGGTCCCGCGGATGACCTGGAGGGCGGCCGGATGGTGCCCATCGGGCGGCCGTTCCCGGGCAGCGCG
CTGTACATCCTGGACTCGCGCGGCGAGCCGGTGCCGCCCGGTGTCCCCGGCGAGCTGTGGATCGGCGGCACCGGG
GTGGCGCTCGGCTACCTCGGCGACGAGGCGCTGACCGGGGAGCGGTTCCTCACCCGCGCCCTGGCCGGCGACGCT
CCGGTACGGCTGTACCGCACCGGTGACCTCGCGCGCTGGGACGCGGCCGGCACCGTCCATCTGCTGGGCCGGGCC
GACTCGCAGATCAAGGTGCGCGGGCACCGCATCGAGATCGGGGAGATCGAGTCGCACCTGGCGGCCTGCCCCGAG
CTGGCCCAGGCGCAGGTCACCGTGCGGCCGGACGCGGGCGGCGAGAACGTGCTGTGCGCGTACGGGGTGGCGGCC
CCGGGCGCCGTGCTGGACTGGCGCGAGGTGCGCCGGCGCCTGGCGGACTATCTGCCGACGTTCATGATCCCCACC
CACTTCACCGAGCTGCCCGCCCTGCCGCTCACCCCGAACGGCAAGGTGGACGTGGCGGCGCTGCCCGCCCCGCGC
ACCGGCGACGGCGCGGACGGGCCGGTGTACGAGGCCCCCGTCACGCTGTACGAGACCCGGATGGCCGAGCACTGG
CAGCGGCTGCTGGGCATCGAGGCCCCCGGGCCCGGTCTGGGCCACGACTTCTTCGAGACCGGTGGCAGCTCCATC
CGGCTGATCGAGCTGATCTACCACCTGCAGGCCGAGTTCGGGATCTCCATCCCGGTCAGCCGGCTGTTCCAGGTG
ACGACGCTGCACGGCATGGCCAAGACGGTCGAGCGGATCGTCACCGGGGAGATCGAGGGGTCGCTGCCGTATCTG
CGGTTCAACGAGAACGCCGCGGCGGGCACGGTGTTCTGCTTCCCGCCGGCCGGTGGCCACGGCCTGGTCTACCGG
GAGTTCGCGGCGCGGCTGCCGGAGTTCGAGTTCCTCGCCTTCAACTACCTGATGGGCGAGGACAAGGTAAGCGGG

```
TACGCCGACCTGGTGGCCGGGCACCGGCCGGAGGGCGAGATCGACCTGCTCGGCTACTCGCTGGGCGGCAACCTC
GCCTTCGAGGTGGCCAAGGAGCTGGAGCGGCGCGGCCGCACCGTGCGCCACGTCGTCATCATGGACTCGCTGCGG
GTGACGGAGTCCTACGAGCTGGGCCCGGAGCACCTGGCCGTCTTCGAGCGCGAGCTGGCCGAGCATCTGCGCAAG
CACACCGGCTCGGCGCTGGTCGCGGAGAAGACGCGCGAACAGGCCAAGGACTACCTGGAGTTCACCGGCCGCACC
GCCAACCCCGGCACCACCGGGGCCCGGATCGCGGTGATCAGTGACGAGGAGAACGCGGCCGCGTACGACAGCGGC
GCCGAGGGCAGCTGGCACGGCGCCTCCCGTACCGGAACCGACGTGCTGCGCGGGGTGGGCCGGCACGCCGACATG
CTCGATCCGGGGACGGTCGAGCACAACGCGCGCCTGGCGCGCGGCATTCTCACCGGCGGTGATGGCGAGGTATGA
```

Gene sequence of the FAD-dependent oxidoreductase *ikaB* from *Streptomyces* sp. Tü6239 (1833 bp; 67.5 kDa):

```
ATGACGCCTTTCGTTCAGCCGGCGGTCGACACCAAGGAGCACAGCGCCATGTCATCCCCCACCACCTCCGGCACC
CCGGGCAGGCAGTCGATGATCATCATCGGCGGCGGCCTGGGGGGCCTGTCCACCGGCTGCTACGCGCAGATGAAC
GGCTACGCGACGCGGGTCTTCGAGATGCACGAGATCCCGGGCGGTTCCTGCACCGCCTGGGAGCGCGGGGACTTC
ACCTTCGACTGGTGCGTCAGCTGGCTGCTGGGCAGCGGTCCCGGCAACGAGATGTACCAGATCTGGATGGAACTG
GGGGCGTTGCAGGGCAAGGAGATGCGCCAGTTCGACGTCTTCAACATCGTGCGGGTGCGCGGCGGCCAGCCGGTG
TACTTCTACTCCGACCCGGACCGGCTCCAGGCGCACCTGCTGGAGATCTCCCCGGCCGACGCCCGCCGCATCAAG
AACTTCTGCGAGGGGGTGCGCACCTTCCAGAAGGCGCTGTCGGTCTACCCGTTCCTCAAGCCGGTGGGGCTGATG
GGGCGGTGGGAACGGTGGAAGATGCTGGCCTCGTTCCTGCCGTACTTCAACGCCATCCGCAAGTCCATCACCGAG
CTGATGACGGACTACGCGGAGAAGTTCCAGCACCCGGTGCTGCGCGAGGCCTTCAACTACGTGCTGTACGAGAAG
CACGCCGACTTCCCCGTCCTGCCGTTCTGGTTCCAGCTGGCCTCGCACGCCAACGGCTCGGCGGGGGTGCCCGAG
GGCGGCTCGCTGGAGCTGGCCCGGTCCGTGGAGCGGCGCTACCTGGGGCTCGGCGGGGAGATCACCTACAACGCC
AAGGTGGAGAAGATCCTCGTCGAGCACGACAAGGCGGTGGGAGTGCGGCTCACCGACGGCCGCGAGTTCCGCGCG
GACATCGTGGTGTCGGCGGCCGATCTGCACACCACCGCCATGGAGATGCTCGGCGGCCGGTATCTCAACGACACC
TGGCGCAAGCTGCTCACCGAGACGATCGACGAGGTGGGCACGATCTCCCCCGGCTATGTCTCGCTGTTCCTGGGG
CTGCGCCGGCCGTTCCCCGAGGGCGAGCCGTGCACCACGTACGTGCTGGAGGACAGCATGGCGGAGAAGCTCACC
GGCATGCGGCATCCCAGCATGAACGTGCAGTTCCGCAGCTGCCACTACCCGGAGCTGTCGCCGCGCGAGACCACG
GTCATCTTCGCCACGTACTTCTCGGAGGCCGAGCCGTGGCGGGCGCTGCGCGACGACGTGCCGGAACAGGCGGGC
CGGGTGCGGCGCGGTCAGGTGCTGCACACCCTGCCGGTGAAGCACGGCAAGGCGTACACCCAGGCCAAGCGGCAG
GCGCGGATCACCATCGAGAACTTCCTGGACGAGCGGTTCCCCGGTCTCAAGGACGCGGTCGCCGTGCGGGACGTG
TCCACGCCGCTGACGCAGGTGCGCTACACGGGCACCTACAACGGCGGGTTCCCCGGCTGGCAGCCGTTCGTGGAC
GGCGGGGAGACCGTGGAGGTGGAGATCAACAAGAACGGCCCGGTGCTGCCGGGGCTCTCCAACTTCTATCTGGCC
GGGGTGTGGGTCACCGTCGGCGGGCTGATCCGGGCGGTGGCCTCGGGCCGGCAGGTCACGCAGGTGATCTGCCGG
GACGACGGGCGGGAGTTCACGGCGAGCGTGGACGAGAGCGCGCCGCCGCCCACCCAGGTCGCCATCCCGGTGGGC
AAGCAGCCGGGCGTGCCGGATCTGGCGGCCGGGTTCCCCGCCCAGACCGCCGGCGGCGAGACCGCCACCGGCGCC
AACAACACCGTCACATCGTCGAGGAGCGTGTAG
```

Gene sequence of the alcohol dehydrogenase *ikaC* from *Streptomyces* sp. Tü6239 (1047 bp; 37.5 kDa):

```
GTGATCGCCGAGCACATCCCGGGGGTCCCGGACACGGACCGGATCTACCGGAAGGTGACGCGGGAGTTCGATCCC
GCCTCGCTGGCGGACGATCAGATGCTGCTGCGCACCCGGTACGTGTCGGTGGACCCGTATCTGGTGGGGCTCTCG
CTCCAGACGCCGATCGGGGACACCGTGCGCGGTGACTCGATCATGGAGGTGGCCGTGGCGGGGCCGCGCGCCCGC
TTCCAGGTCGGGGACCTGGTGCAGGGGTACGGCGGCTGGTGCAGCCATCTGGTGTCCACCGGGGGGCCCAGCGGA
TGGAACGACGACGGCGCCGAGTTCGCCGTCCAGTTGCCGCCGTTCCGCAAGCTGGACCCGCGGCGGTACGACGAG
GCGCTGCCGCTGTCCACGGCGCTGGGCGTGATGGGCACCCCGGGCATCACCGCGTTCGGCGCGATGAAGACGTTC
CTGACCGTGGGCTCCGAGGACACGGTGGTGATCAGCGGGGCGTCCGGGACGGTGGGCACCCTGGTGGGCCAGCTC
GCCAAGCGGGCCGGGGCCCGGGTGGTGGGCACCACCTCCTCGCCGGGGAAGGCCGCGTATCTGACGCAGCTGGGC
TTCGACGCGGTGGTGAACTACCGGCAGGGCGACGACACGGACACGGTGCGCGAGGCGCTGGCGGCGGCAGCGCCC
AACGGAATCGACAAGTACTTCGACAACCTGGGCGGCACCGTGACGGACGCGGTGTTCACGATGCTCAACGTGCAC
TCCCAGGTGGCGGTGTGCTGGCAGTGGGCCACCACGGTCAACGGGGACTGGACGGGGCCGCGGCTGCTGCCGTAC
ATCATGTTCCCGCGCACCACGATCCGGGGGATCTTCGCCGACGAGTGGTACACGGAGGAGATGGTCGACGCGCTG
CACGAGGAGGTGGGCGGGCTGATCCGCAAGGGTGAGCTGGCCTACCACCAGACCATCCACCAGGGCTTCGACGCC
CTCCCGGACGCGTACCGCTCCCTGTACACCGGCCAGGAGGGCAACCGCGGCAAGGTCCTGGTCGCCCTGTAG
```

Gene sequence of the monooxygenase *ikaD* from *Streptomyces* sp. Tü6239 (1224 bp; 45.7 kDa):

```
ATGCCCGGACAGCAGGAACAGCAGGCACCGTCGGAGCACCCGGAGCAGGAACTCCTCACCTTCCCCTTCCCCTCG
ACGGGCCTGGAGTTTCCCCCCGTCTACCACGAGCTGTACCAGCAGCGGCTCACCAAGGTCCGGCTCCCGTACGGC
GACGACGCCTATCTCGCCATCCGGTACGCCGATGTGAAGACCGTGCTGTCCGACTCCCGGTTCTCCATCGTCGCC
TCCCTCGGCCAGGACCAGCCGCGCACCCGGGCCGGGGCCCGCGTCGGCAACGGCCTGTTCTCCCTCGACCCCCCG
CAGCACTCCCGGCTGCGCTCCGTCCTGGGCCGGGACTTCACCCCGCGCCGGGTGGAGAAGCTGCGCGAGCGGGTG
CGGGAGCTGACCGACCAGTGCCTGGACCGCATGGAGGCCGCCGGGTCCCCCGCCGATCTCGTCGCCCACCTCGCC
GTGCCGATGCCCACCGCCGTGGTCTGCGAGATGATGGGCGTACCCGAGCCCGACCACCACCTGTTCTGGGGCTGG
GCCGAGACGATCCTGTCGAACGACACCACGCCCGACGACCTCATCCGGCGCTACCAGGAGTTCACCGCCTACATG
GGCGGCATGGTCGAGGAGCGCCGCGCCCGTCCCACCGACGACATGTTCGGCATGCTGGTGCGGGCCTGCGACGAG
GAGGGCCGGATCACCGAGATCGAGATGCACGCGCTCGCCTCCGACCTGCTCAGCGCCGGCTTCGTCAGCACCGCC
CACCAGATCGCCAACTTCACGGCCATGCTGCTGGCCCGCCCCGAACGGCTCCAGCCCCTGGTGGACAAGCCGGAG
CAGATCCCGGCCGCCGTCGAGGAACTCATGCGCCACGTCCCGATCCTCAGTGGCTTCTCCTTCCCCCGCTACGCC
ACCGAGGACCTGGAGATGAGCGGGGTGACCGTCCGCCGCGGCGAGGCCGTCATCCCGGTGATCGCCGCCGCCAAC
CGCGACCCCGACGTCTACCCGGACGCGGGCCGCCTCGACCTCGAACGCAACGGGCTTCCGCACCTCGGCTTCGGC
CAGGGCCCGCACTTCTGCATCGGCGCCCATCTGGCCCGGGTCGAGCTGCAGGTGGTCCTCGAAGCCCTCACCGAG
CGCTTCCCCGACCTGCGCTTCGGCGTCCCCGAGAACGCCCTCAAGTGGAAGCAGGGCCACTTCATGAACGGCCTG
CACGAACTGCCGGTCGCCTGGTAG
```

Gene sequence of the sterol saturase *ptmD* from *S. pactum* SCSIO 02999 (921 bp; 33.4 kDa):

```
ATGGAGATTCTCCGCATGGAAGATCGACCCGATACGGGGTCGCTGCCGGCGAATGTGTGGACCGCCGGCGCGTCC
GCCCGCGAGTGTCTCCGCTATGCCGCGTACCCGATTCTGCTCTTGTCCGCAGTCTGGCTCTTCGCCTCGGTTCTG
CGCTTCGGCTGGGACCGGGGCCAGGCCATCCAGCTCTTCCTGATCGGCACCATCGTCTATCTCGCGGCGCTGGAG
CGGCTGATCCCGCACCGGACCGACTGGCACCCGAGCGGTCGGGAACTGTGCTGGTACGCAGCCTATTTCGGGTTC
ACCATGGTCGGCGCCGTGCTCGGCCAGTCGGTCGTCGCGGCCGTTCTGGCCGCGACCCCCGTCACGGGACCGGGG
CTCGCGCTCGGGGCCGAGGTGCCCCTCGCGCTGCTGGCCTCCTCACTGACCGGCTACCTCGCCCACCGCTGGGCG
CATTCCAATCGGTGGCTGTGGAAGGTACACGGAATTCATCACGTCCCCGGAAAGGTGAACGTCGCCAATAACGGC
GTCAACCACCTACTGGATGTCGCCTTCAAACAGGGCGCGGTCCAATTGACACTGGGATTTCTCGGATTCTCCGCC
GACTGCCTTTTCGCGGTGGCGCTCTTCACCCTTGTCCAAGGATATTTCGTGCACGCGAACGTGGACGTCCGACTC
GGTCCGCTCCACCACGTACTGGCCAGCCCCGAGCAGCACCGGCTGCACCACAGCGCCGACCTGGCCGAGGCCGGT
CACTTCGGCGTGGACCTCTCGGTCTGGGACCGCCTCTTCGGCAGTTTCACCTGGCGACCGGACCGCCGCCCGGCG
ATCGTCGGTGTCAAGGACCCCGCCACGTTCCCGGAGACCGGCTCGATCGTCGCCAGCCTGCTCCACCCCGTGCGC
CGCCCGACCCACACCGCGTAG
```

Gene sequence of the monooxygenase *cftA* from *Streptomyces* sp. JV178 (44.0 kDa; 1191 bp):

```
ATGTCGGATCAACACCCCCCACTCCCCTACCCCTTCGCCCCACGCGGCCTCGACCTCGACCCCACCTACGCGGAG
TTGCGCGACCGCGCACCGGCCCGTATCCGTATGCCGTACGGCGACGACGCCTGGCTGGTCACCCGGTACGAGGAC
GTACGGACGGTCCTGGCCGACCCGCGGTTCAGCCTGGCCGCCTCGATGGGCCGGGACCAGCCGCGGATGCGCCCG
GTGGCCCGTACCGGCGCCGGCCTGTTCTCCACGGAGCCGCCCGACCACACCCGGCTCCGCTCACTGGTCGCACGG
CAGTTCAGCGCCCGCCGGGTCGAGCCGCTGCGCGCACGGGCCGGGGAGCTGGCCGACGAGCTGATCGACGGGATG
GTGGCCGCAGGCCAGCCGGCCGACCTGGTCGAGGACTTCGCCATCCCCATGCCGACCACCATCATCTGCGAGGTG
CTCGGCATCCCCGCCAAGGACCACCGGATGCTCTGGCACTGGGCGGAGACGGTGCTCTCCGCCATCACCCCCCAG
GAGGTGCTCGCCACCGAGGGCCGGGCCTTCATGGAGTACATGGTCGGCGTGCTGGAGCTGCGGGGCCGCGAGCCG
GGCGACGACCTGCTCACCACCCTGGTCCGGGCCTGCCGCGAGGAGGGCCTGATCAGCGAGGAGGAGCTGCTGTCG
ATCGCCTGCGACCTGCTGATCGCCGGCTTCGTCTCCACCACCAACCAGATCGGCAACTTCTTCCACCAACTCCTC
GTACATCCAACGGAGTTGACGCGGCTACGGGAGCGCCCCGAGCTGATCCCGAAGGCCGTAGAGGAGCTGATGCGC
TACGTGCCACTGCTCACCGGCTTCAACCTGGCACGGTACGCGACCGCCGATGTCGAGCTGGGCGGCATCACGATC
CGGGCCGGCGAGGCGGTGATGATCGCCACCGCCGCCGCCAACCGCGACCCGGGGGTGTTCCAGGAGCCGGAGCGC
CTCGTCTTGGACCGCGACGCCAACCCGCACATCGGCTTCGGCCACGGCGTGCACTACTGCGTCGGCGCCCATCTG
GCCCGGCTGGAGCTCCAGGTGGCGATCGAGCGGGTGCTGCACCGGCTGCCCGGCCTACGGCTGGCCGTCCCCGAG
AGCGAACTGAGCTGGAAGCAGGACGCGATGGTCAACGGTCTCCAGGCACTCCCGGTCGCCTGGTGA
```

Sequences of the used promoters

Table S9. Strong, constitutive active promoter sequences used for plug-and-play system. The promoter sequences were elongated by restriction sites (underlined; StuI: AGGCCT and XbaI: TCTAGA), and RBS (bold) were added if not already present.

Promoter	Sequence
**ermE***	AGGCCTGCGGTCGATCTTGACGGCTGGCGAGAGGTGCGGGGAGGATCTGACCGACGCGGT<br>CCACACGTGGCACCGCGATGCTGTTGTGGGCACAATCGTGCCGGTTGGTAGGATCCAGCG<br>GAGCAACGGAGGTACGGATCTAGA
**gapdhP(EL)**	AGGCCTGCTGCTCCTTCGGTCGGACGTGCGTCTACGGGCACCTTACCGCAGCCGTCGGCT<br>GTGCGACACGGACGGATCGGGCGAACTGGCCGATGCTGGGAGAAGCGCGCTGCTGTACGG<br>CGCGCACCGGGTGCGGAGCCCCTCGGCGAGCGGTGTGAAACTTCTGTGAATGGCCTGTTC<br>GGTTGCTTTTTTTATACGGCTGCCAGATAAGGCTTGCAGCATCTGGGCGGCTACCGCTAT<br>GATCGGGGCGTTCCTGCAATTCTTAGTGCGAGTATCTGAAAGGGGATACGC**CGAGCAACG<br>GAGGTACGGAC**TCTAGA
**rpsLP(XC)**	AGGCCTGCCCTGCAGGCGGAAGTCAGGTAGACACGACTTCCGCTAGTCCTTGCAAGGTCT<br>GCTGACGTGAGGCGGGGCGGTCGTTTTTGACCGCCCCGCCTTCGTCATGTAGGCTCGCTC<br>GCTGTGCCTGGCGTGTCTTCAGACGCCCAGGTCCCGGTGCCGTGAGGCCCGGGCCATCGA<br>GCCGGTGGTACGTGGCTGCGGTCCCCTTGTGAGGGCTGCGCGCCGTGTGCTGTCCGGCGC<br>GCACAGCCTTGAATCCACCCGCGGGGGCCGGCCGGTCTCCGTGAGCTCGAGAAGACGACG<br>GAGACGTA**CGAGCAACGGAGGTACGGAC**TCTAGA
**kasOP***	AGGCCTTGTTCACATTCGAACGGTCTCTGCTTTGACAACATGCTGTGCGGTGTTGTAAAG<br>TCGTGGCCAGGAGAATACGACAGCGTGCAGGACTGGGGGAGTT**CGAGCAACGGAGGTACG<br>GAC**TCTAGA
**SF14P**	AGGCCTCCTATCCAGGAGATATTATGAGTTACGTAGACCTACGCCTTGACCTTGATGAGG<br>CGGCGTGAGCTACAATCAATACTCGATTA**CGAGCAACGGAGGTACGGAC**TCTAGA
**P-2**	AGGCCTGCCCGGCCATATCCGGCCCGGCCAAATCTCGGCCGGCCACCTCGGCCTGGCCAG<br>CCTGGCCCGGCCAATCTCGGCCCGACCAACTTCAGCCCGGCCGGCGCTTGAGGCCGATGA<br>GCCGCGGAGCGGCGAGTCTTCCGCCCGGCCGGTCCGGGTGGCCTCAAGCGCCGGCCGGGC<br>TGGTTTTGGTGCGGACACGTCTGACCGTGCCGGTCACCGATGGCCTCAAGCGCCGGCCGG<br>GCTGGGAGTGGTGGCCGAGGCTTCGGGCGTACGTGCCAGCCCGCAAGGGGCTGCGGTGGG<br>GTGGCCTCAAGCGCCGGCCGGGCTGAGGTTGGCTGGCTGGGCCGGGTTCGGCCGGTGGGT<br>CGAGGTGGCCTGGCCGGGCTCGCCAGGGTGAGTTGGCCGACGGGCCGAGGCGGCCCGCCC<br>GGGCTCCCCGGGCCGAGTTGGCGCGGCCAGGCCAGGGCTCAGCAGGGTGGGGGAGTGGGG<br>CAGGCGGCCCGGTAGGGGAGTGCGGGAGGGCAGCGCGCGCCGCGCGCATTGGCACTCCGC<br>TTGACCGAGTGCTAATCGCGGTCATAGTCTCAGCTCTGGCACTCCCCGCAGGAGAGTGCC<br>AACACAGCGACGGGCAGGTCCGGCACCCGCGACGACGGATCGACCTGGTCGCCACACTCA<br>GATCAGTTAACCCCGTGATCTCCGAAGGGGGAGGTCGGATCTCTAGA
**P-6**	AGGCCTGGCGCCGACCGCACCACACTCACGAGGGCCCGCCCCACCAACAGGGGGCGGGCC<br>CTCTGTGCTGGCCTCAGGCGCCGACCGGGCTCGGTGCCCTCAAGCGCCGGCCGGGCTCCA<br>AGGGTGGCCTCAAGCGCCGGCCGGGCTGAGTTGGGCCGGTCTGGGCCCGCACGCGCGCCT<br>CACTGACGGCCTCAAGCGCCGGCCGGGCTATCTATAGCCCGGCCGGCGCTTGAGGCCGTC<br>TTTGGCGCGCGCCTGTGAGCGGACGGCCCGTCAAAGATCAGCCCGGCCGGCGCTTGAGGC<br>CATCTTTCGAGCCCGGCCGGCGTTTGAGGCCACCCCACCCCCGCCCCGGCAGGGGCGGCC<br>TGACCTCCGCATCCGCCGGCGCGGACAGGGCACCCCCAGTAGACGGGCGCGGGGCCGGAG<br>GCCCCTAGCGCCTTGCACTCTCCTACCCCGAGTGCTAATTATTGGCGTTAGCACTCTCCG<br>AGTGAGAGTGACAGAAGGACCGGGTCGGTGAGGCCCGCTGGCCACGCGGGGCAAGGAACC<br>GCGAGGCAGGCAGGCCGTCCGTCGCGGGCGCCAGCACGGTCCGGAGTATCCACCCTCCCC<br>CAGACAGAGTCCGGGGGGACCCCCAGTCCTGGGAGGACCACTTCACTCTAGA
**P-15**	AGGCCTTCCGCGCCGCCGGCCCGACGGTGCCCGGCCCCGTACCCCCCCCGGGTGGTGCGG<br>GGCCGGGCACCGGCCTTTTGGCGCTGCGGAGTTGACGGAAGTTGGCCGAACCGGATGCGC<br>TCGGCGCCCGGGGGCTGAAAGATGCTCACAGCCCCTTTCCACGGCGGTCCGGGAGGGGAG<br>GCCGGGCAACCGGTTTTCGGGGGCGGAGTGTCCGGTATGCGGACGGCCGCGCCCGATAGA<br>TGTGTAACGAGTCCGTTTCGCAACCATCTATCTCGGATCGGTTTGTCCGGATTTTGGAAG<br>ATGTGAGTGTCAGGTGTGATCGAACCGAGACCAAAAGGGTGTGGTCGGGCCGAACACCAT<br>GGCTAATAGTTGAGCGCGTAGAGCTCGGGTCAATGGGTCACGCGCTGTGGGGAGCGCCGA<br>CTCACGAGCACACTGGGGCACTCGATCTTCGCCGTCAGGGGTGTCGGCGGATCGTCCTGT

GCCCTCTCTTGCAGTGAACAAGTGGACTCATGAGGAGGAACCCTCTAGA

P-31

AGGCCTCCGGACCTCTCCTCACGCTCACCCTGCGCGCTTCCGCGCGACAGGCACAATTAC
CCGTATATGTCCCGACTCGCCCACAGTCTCCGCCTTCGGCCGGGTCATTCCCCCGACCGA
CCCGGCCCGGCCCACCCATTTCCGGCCCGGCCGGCGTTTGAGGCCGACCGGTGACGGACA
CCCGAAGCCCTCGGAGCGCGCTCGGCATCAGCCCGGACGACGCTTGAGGCCACCTCGACC
GCCGCCGGACGGCTTCATCCGAAGTGCCTCTGAACTGGTAAAACGAGCCGTGCTGGCAGC
TCTCTGCACAACCAGGCAGAACAAAACTTGAGCCCGTCCGACTCAACCGCATTGACGCGC
CGCGTCCCCTCGTGCATCCTTGAGTGAGTTCCACTCAAGTAGTCAGCTGGAGGAATTGAC
TCTAGA

7. Cloning

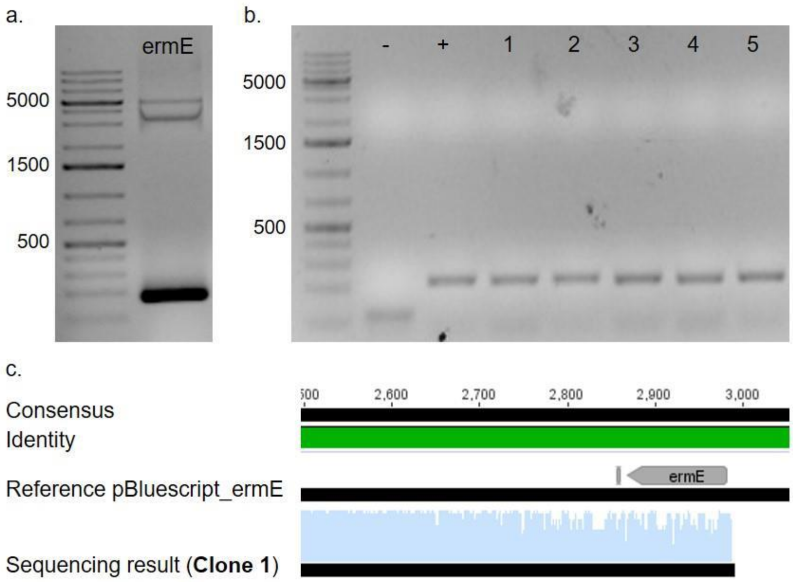

Figure S42. Assembly of pSET152-ermE* expression vector. **a.** Amplification of the ermE* promoter (183 bp). **b.** Colony PCR of clones obtained after transformation (positive: 266 bp; negative: 151 bp). **c.** Sanger sequencing revealed 100% identity with the reference sequence (green).

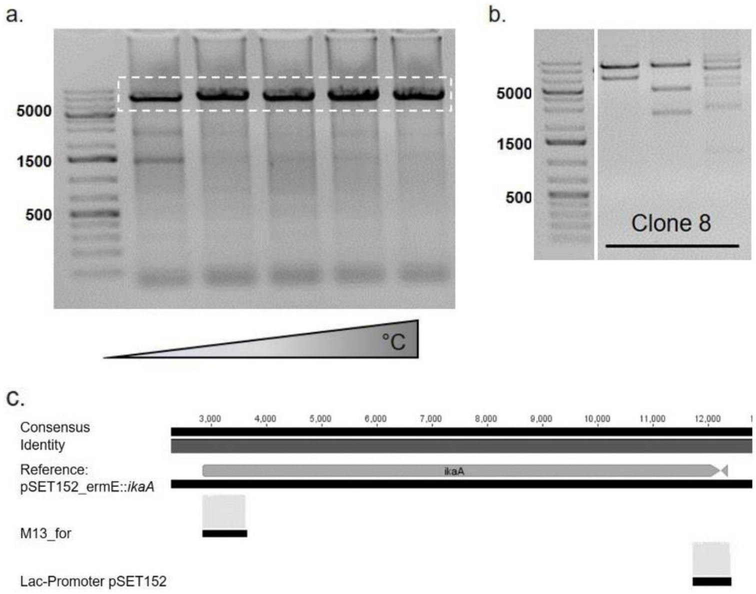

Figure S43. Assembly of pSET152-ermE*-*ikaA* expression vector. **a.** Gradient PCR (50-70 °C) for the amplification of *ikaA* (9375 bp), suitable for Gibson assembly into pSET152-ermE*. **b.** Analytical restriction digest (NsiI/StuI, NcoI/PciI, KpnI/BciVI) with clone 8 showing the predicted restriction pattern of pSET152-ermE*-*ikaA*. **c.** Sanger sequencing revealed 100% identity with the reference sequence.

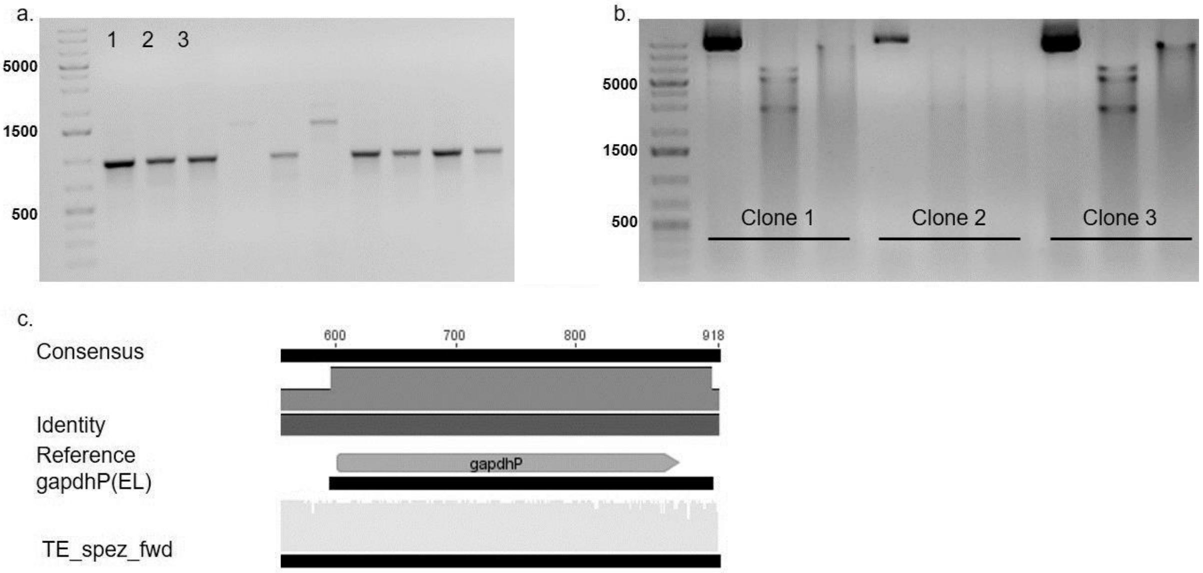

Figure S44. Verification of the cloning of pSET152-ermE*-*ikaA*-gapdhP(EL). **a.** Colony screening PCR with expected bands for clones 1, 2, and 3 (919 bp). **b.** Analytical restriction digest (uncut, StuI/XbaI, PvuI/EcoRI) with clone 1 and 3 showing the predicted restriction pattern. **c.** Sanger sequencing of clone 1 revealed 100% identity with the reference sequence.

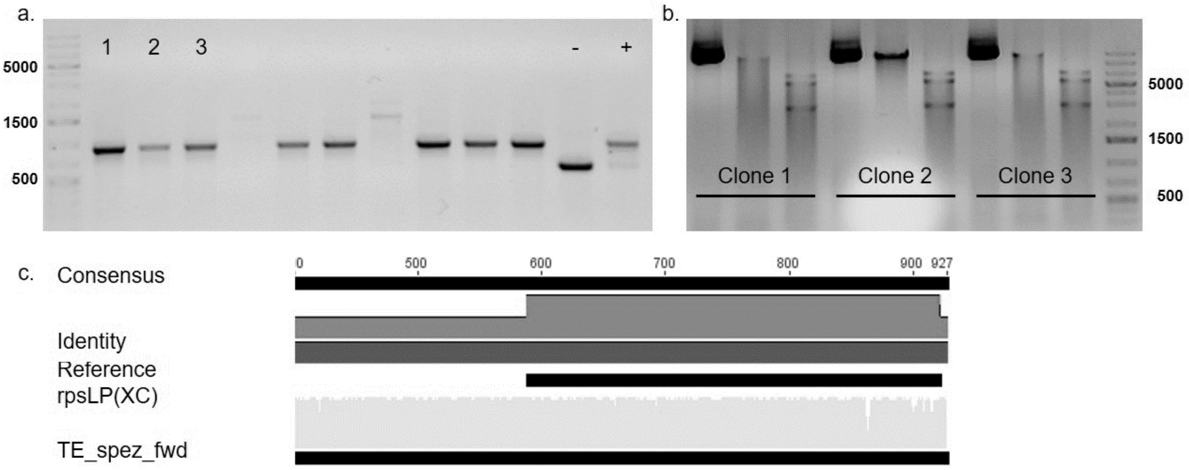

Figure S45. Verification of the cloning of pSET152-ermE*-*ikaA*-rpsLP(XC). **a.** Colony screening PCR with expected bands for clones 1, 2, and 3 (936 bp). **b.** Analytical restriction digest (uncut, StuI/XbaI, PvuI/EcoRI) with all clones showing the predicted restriction pattern. **c.** Sanger sequencing of clone 1 revealed 100% identity with the reference sequence.

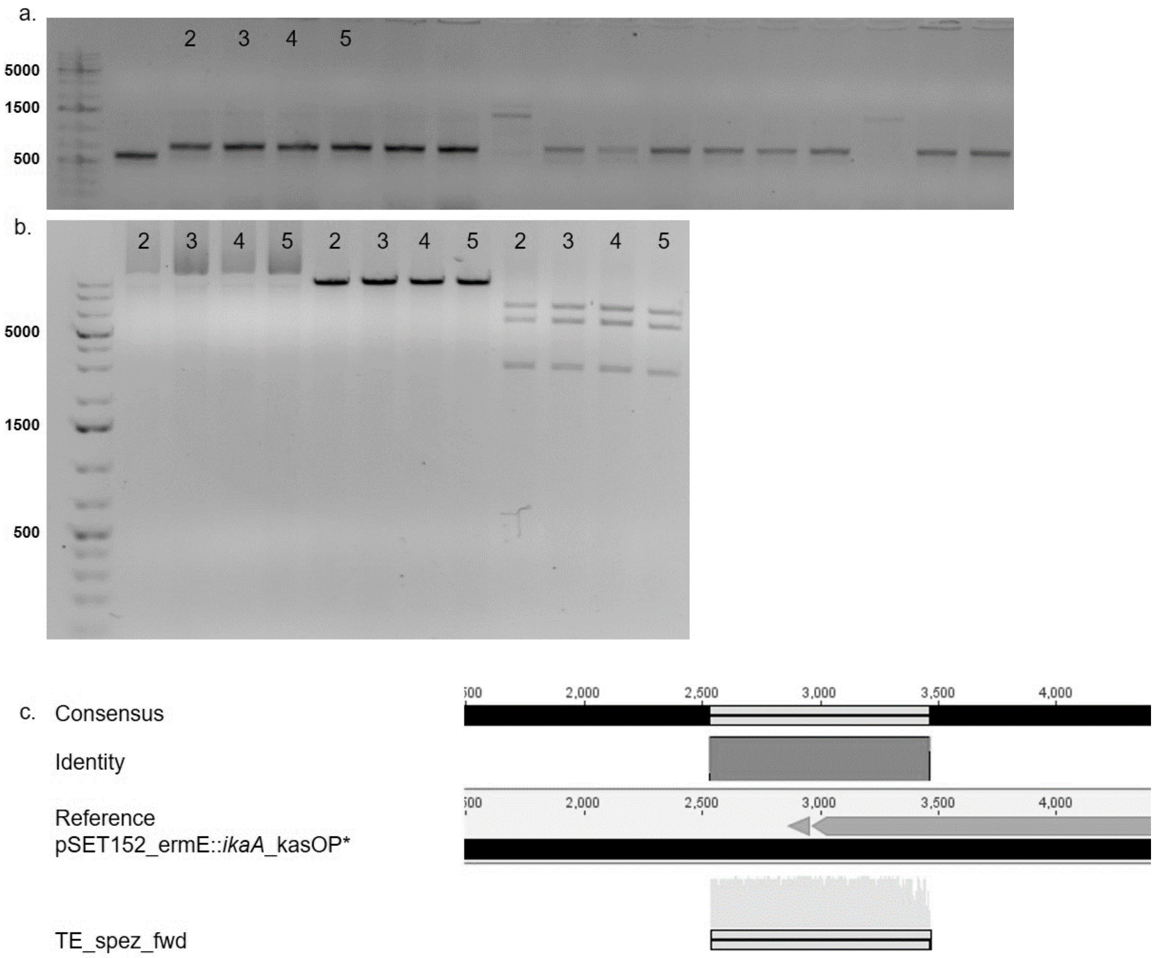

Figure S46. Verification of the cloning of pSET152-ermE*-*ikaA*-kasOP*. **a.** Colony screening PCR with expected bands for clones 2, 3, 4, and 5 (731 bp). **b.** Analytical restriction digest (uncut, StuI/XbaI, PvuI/EcoRI) with all clones showing the predicted restriction pattern. **c.** Sanger sequencing of clone 2 revealed 100% identity with the reference sequence.

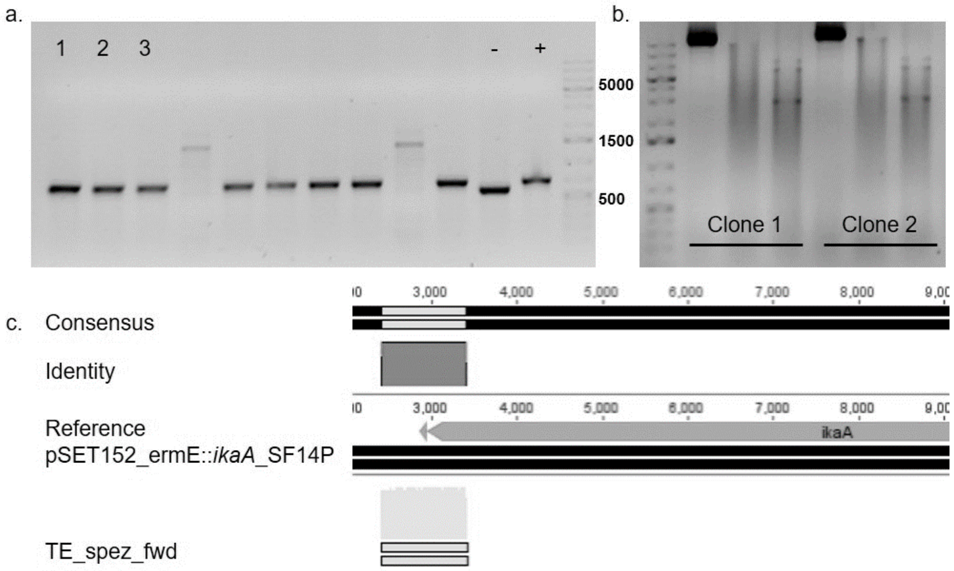

Figure S47. Verification of the cloning of pSET152-ermE*-*ikaA*-SF14P. **a.** Colony screening PCR with expected bands for clones 1, 2, and 3 (717 bp). **b.** Analytical restriction digest (uncut, StuI/XbaI, PvuI/EcoRI) with all clones showing the predicted restriction pattern. **c.** Sanger sequencing of clone 1 revealed 100% identity with the reference sequence.

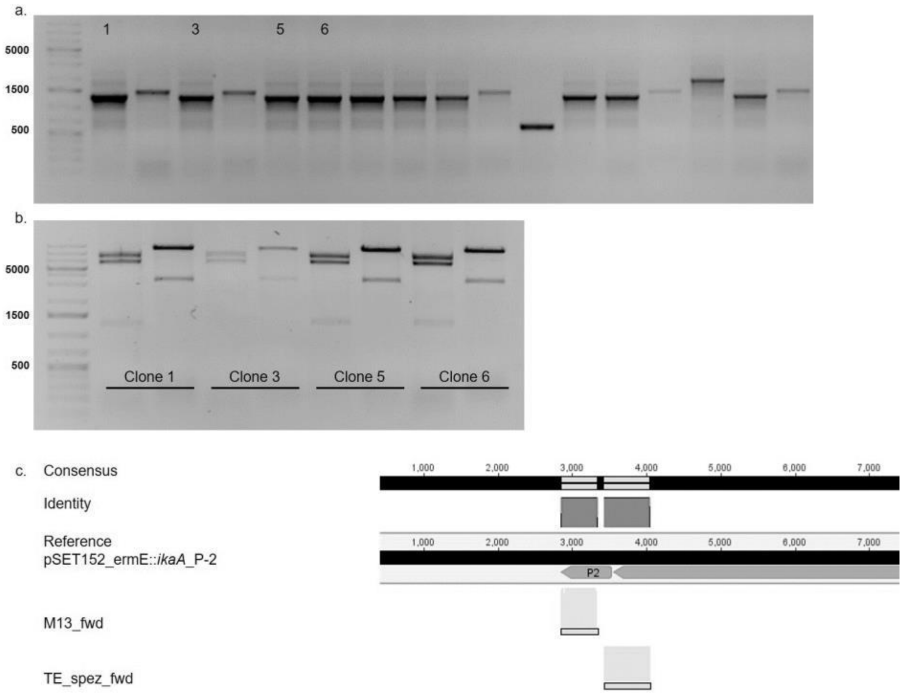

Figure S48. Verification of the cloning of pSET152-ermE*-*ikaA*-P-2. **a.** Colony screening PCR with expected bands for clones 1, 3, 5, and 6 (1309 bp). **b.** Analytical restriction digest (NcoI/XbaI, PvuI) with all clones showing the predicted restriction pattern. **c.** Sanger sequencing of clone 1 revealed 100% identity with the reference sequence.

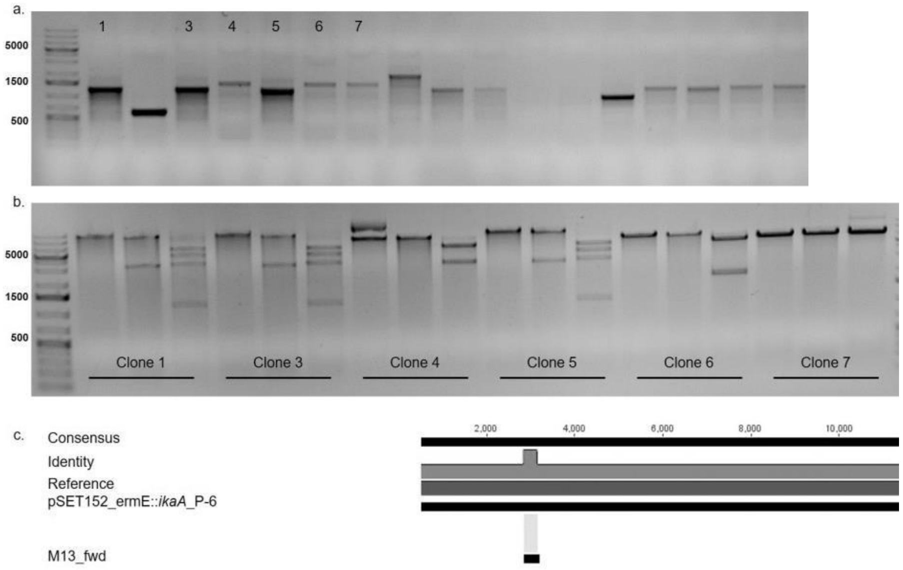

Figure S49. Verification of the cloning of pSET152-ermE*-*ikaA*-P-6. **a.** Colony screening PCR with expected bands for clones 1, 3, 4, 5, 6 and 7 (1254 bp). **b.** Analytical restriction digest (XbaI/StuI, PvuI, XmnI) with clones 1, 3, and 5 showing the predicted restriction pattern. **c.** Sanger sequencing of clone 1 revealed 100% identity with the reference sequence.

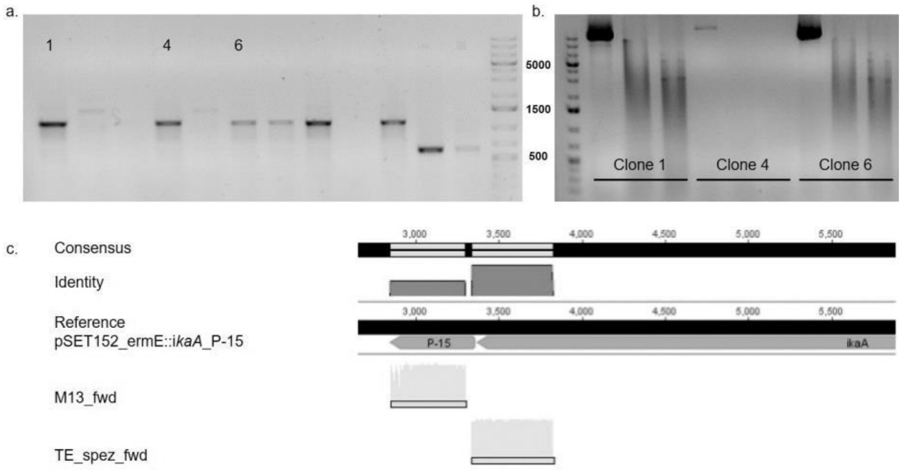

Figure S50. Verification of the cloning of pSET152-ermE*-*ikaA*-P-15. **a.** Colony screening PCR with expected bands for clones 1, 4, and 6 (1131 bp). **b.** Analytical restriction digest (uncut, StuI/XbaI, PvuI/EcoRI) with all clones showing no conclusive bands, but **c.** Sanger sequencing of clone 1 revealed 100% identity with the reference sequence.

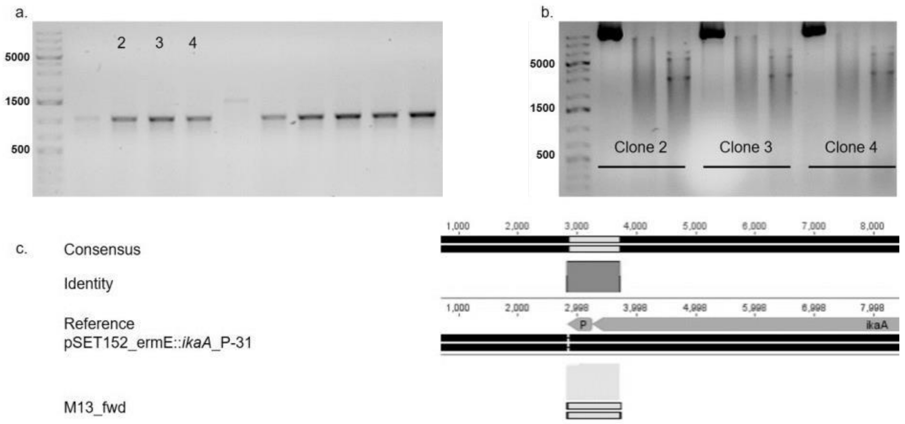

Figure S51. Verification of the cloning of pSET152-ermE*-*ikaA*-P-31. **a.** Colony screening PCR with expected bands for clones 2, 3, and 4 (1028 bp). **b.** Analytical restriction digest (uncut, StuI/XbaI, PvuI/EcoRI) with all clones showing the predicted restriction pattern. **c.** Sanger sequencing of clone 2 revealed 100% identity with the reference sequence.

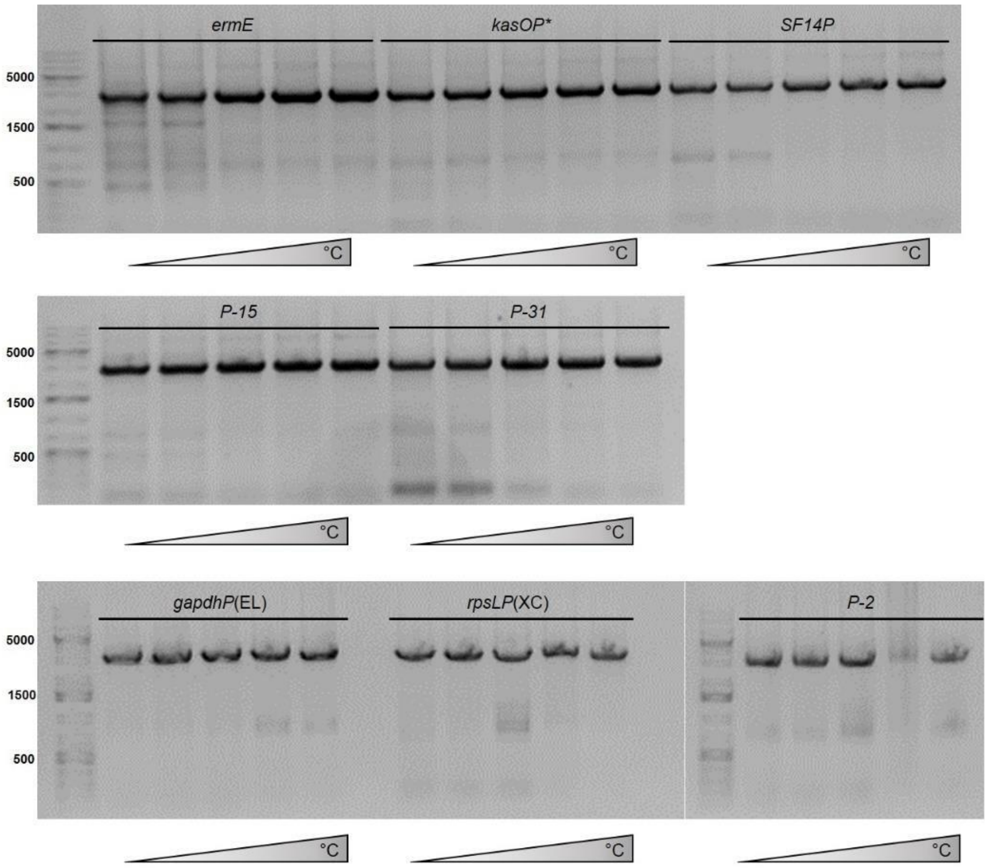

Figure S52. PCR amplification of *ikaBC*. The two genes (2937 bp) were amplified together for the eight different expression constructs and simultaneously homologous arms were added for SLIC cloning and an annealing temperature of 50–65 °C.

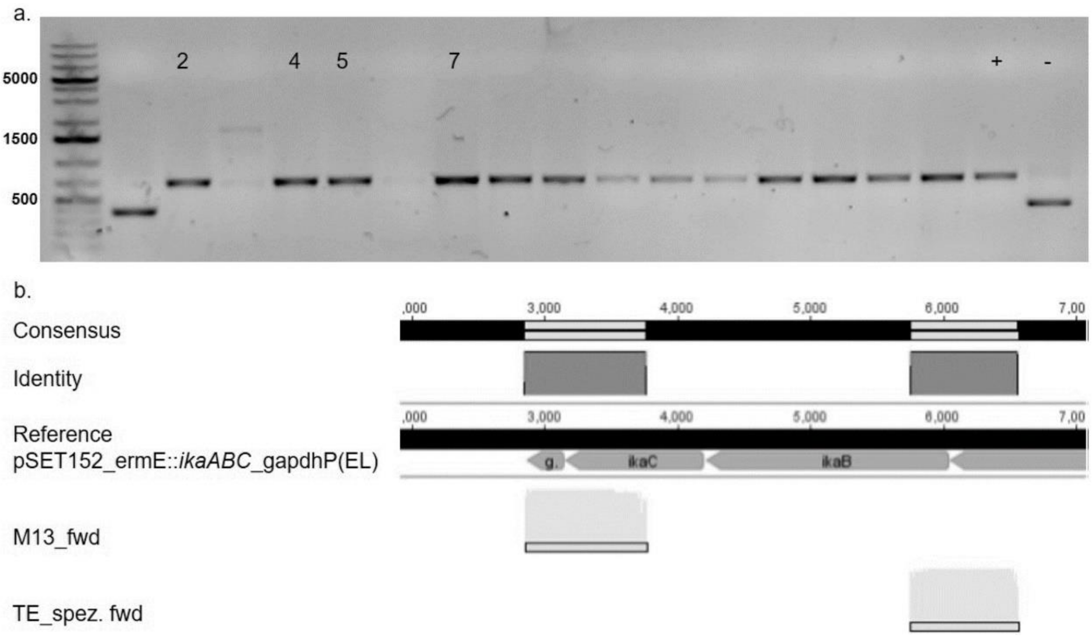

Figure S53. Verification of the cloning of pSET152-ermE*-*ikaABC*-gapdhP(EL). **a.** Colony screening PCR with expected bands for clones 2, 4, 5, and 7 (677 bp). **b.** Sanger sequencing of clone 2 revealed 100% identity with the reference sequence.

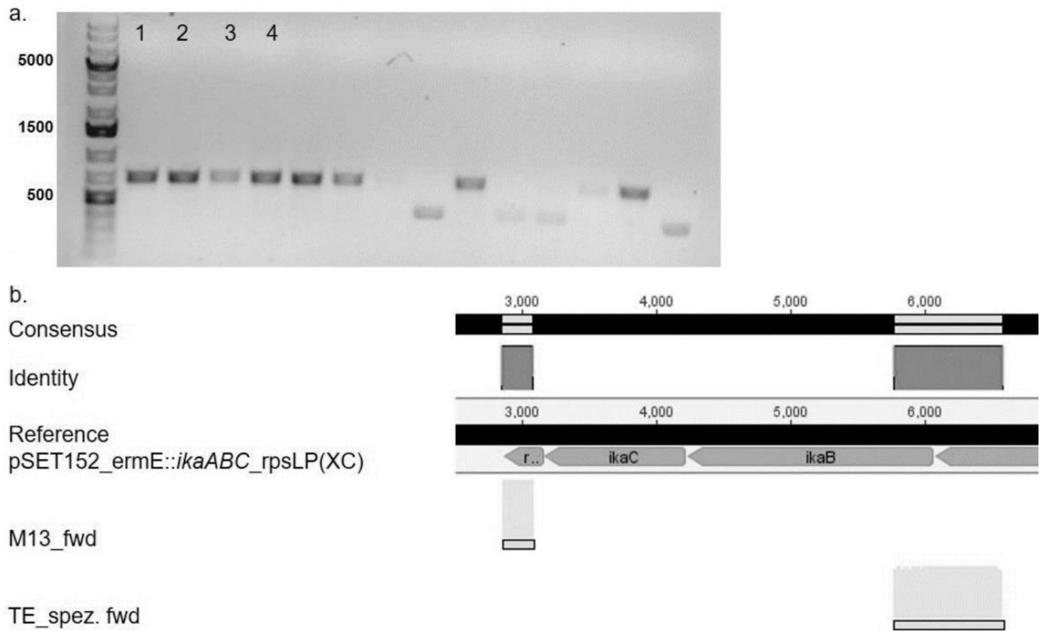

Figure S54. Verification of the cloning of pSET152-ermE*-*ikaABC*-rpsLP(XC). **a.** Colony screening PCR with expected bands for clones 1, 2, 3, and 4 (694 bp). **b.** Sanger sequencing of clone 1 revealed 100% identity with the reference sequence.

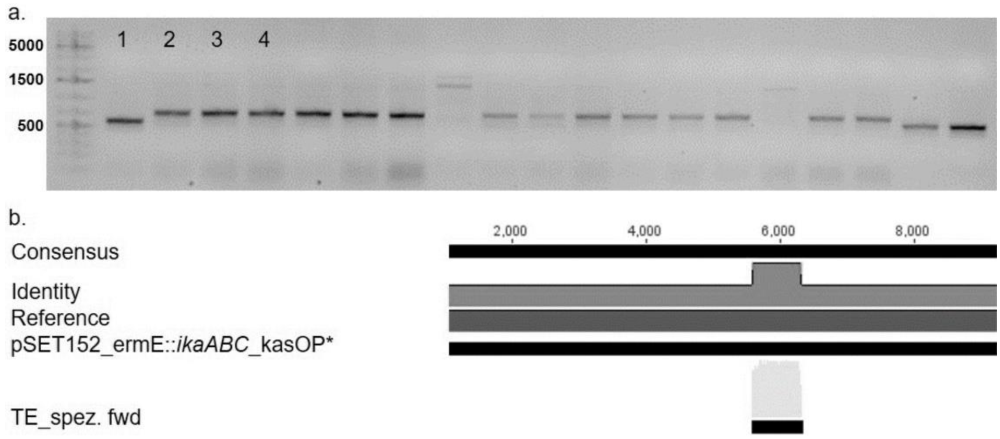

Figure S55. Verification of the cloning of pSET152-ermE*-*ikaABC*-kasOP*. **a.** Colony screening PCR with expected bands for clones 2, 3, and 4 (731 bp). **b.** Sanger sequencing of clone 3 revealed 100% identity with the reference sequence.

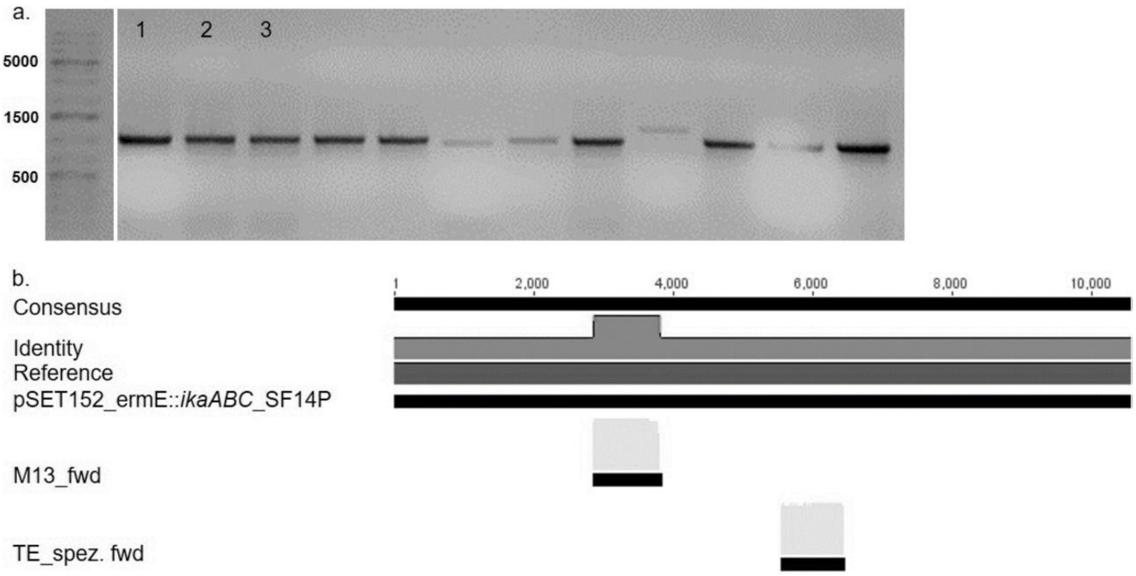

Figure S56. Verification of the cloning of pSET152-ermE*-*ikaABC*-SF14P. **a.** Colony screening PCR with expected bands for clones 1, 2, and 3 (1082 bp). **b.** Sanger sequencing of clone 1 revealed 100% identity with the reference sequence.

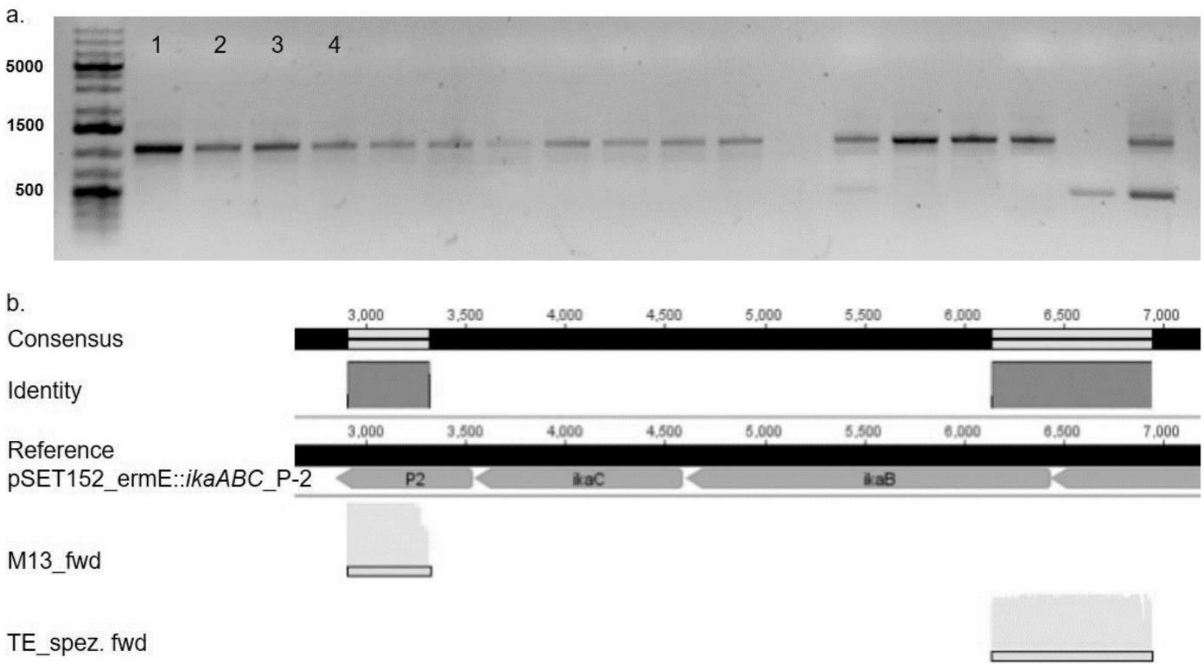

Figure S57. Verification of the cloning of pSET152-ermE*-*ikaABC*-P-2. **a.** Colony screening PCR with expected bands for clones 1, 2, 3, and 4 (1067 bp). **b.** Sanger sequencing of clone 1 revealed 100% identity with the reference sequence.

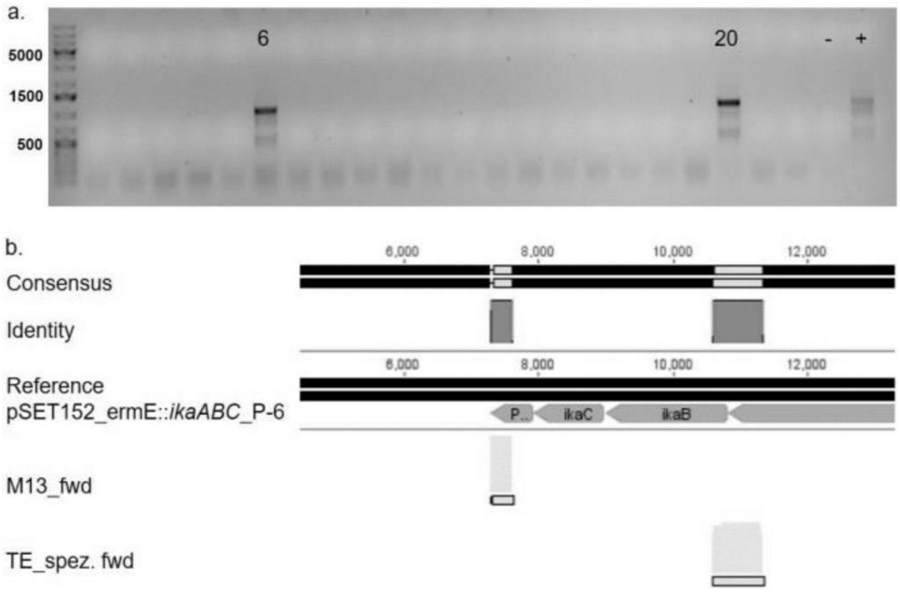

Figure S58. Verification of the cloning of pSET152-ermE*-*ikaABC*-P-6. **a.** Colony screening PCR with expected bands for clones 6 and 20 (1012 bp). **b.** Sanger sequencing of clone 20 revealed 100% identity with the reference sequence.

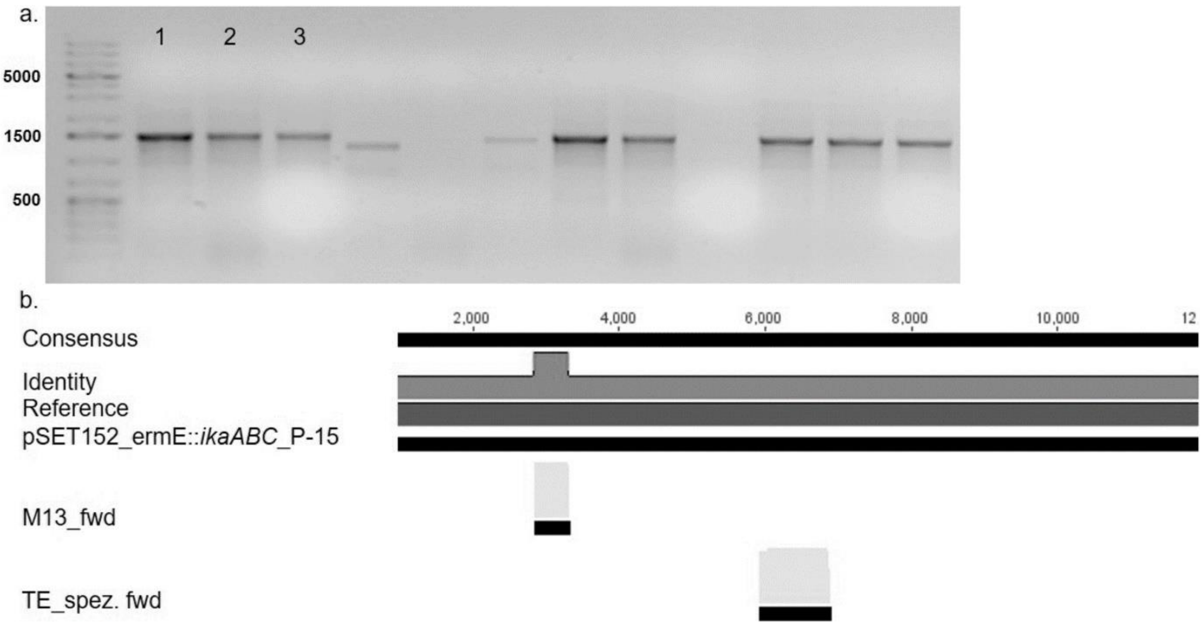

Figure S59. Verification of the cloning of pSET152-ermE*-*ikaABC*-P-15. **a.** Colony screening PCR with expected bands for clones 1, 2, and 3 (1496 bp). **b.** Sanger sequencing of clone 1 revealed 100% identity with the reference sequence.

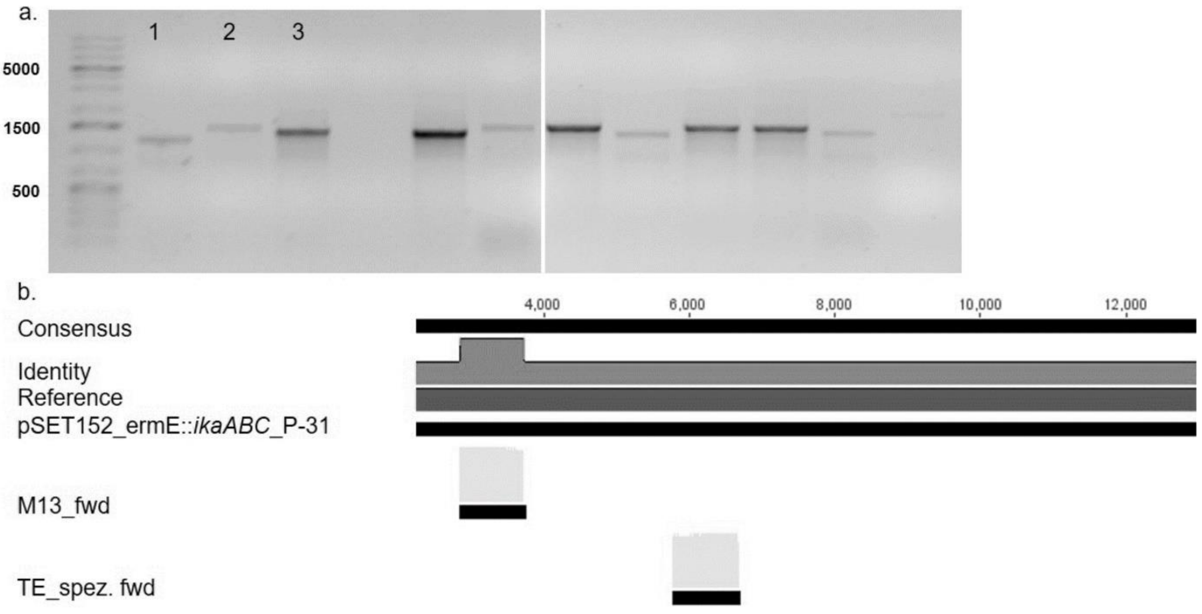

Figure S60. Verification of the cloning of pSET152-ermE*-*ikaABC*-P-31. **a.** Colony screening PCR with expected bands for clone 1 (1393 bp). **b.** Sanger sequencing of clone 1 revealed 100% identity with the reference sequence.

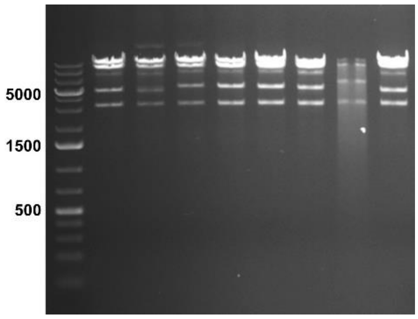

Figure S61. Analytical restriction digest of the plasmids pSET152-ermE*-*ikaABC*-P2. The second promoters (P2) were gapdhP(EL), kasOP*, P-2, P-6, P-15, P-31, rpsLP(XC), and SF14P (from left to right). All plasmids were digested with SphI/EcoRI and showed the predicted restriction pattern.

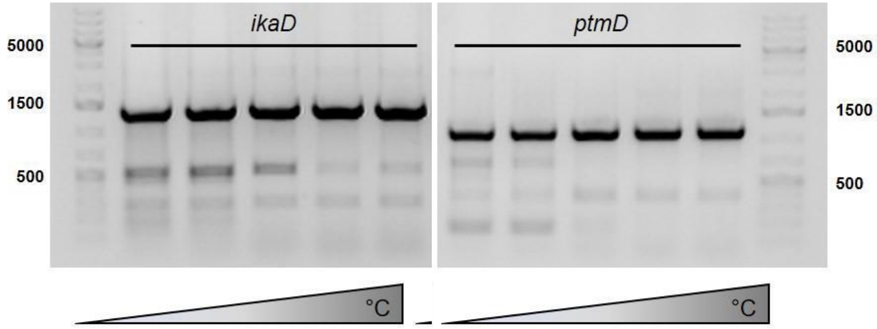

Figure S62. Amplification of *ikaD* (1388 bp) and *ptmD* (1085 bp). Annealing temperature 50–65 °C.

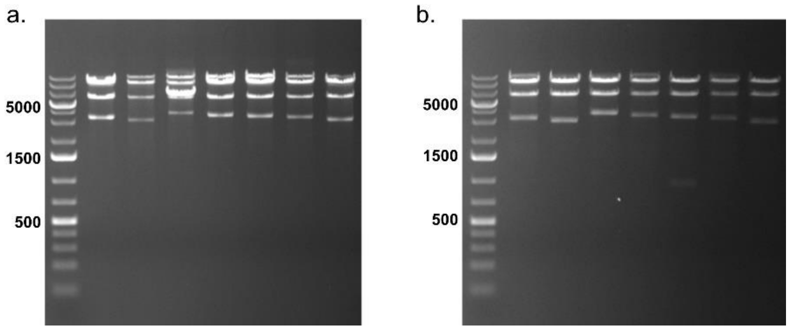

Figure S63. Analytical restriction digest of the final expression plasmids containing the modifying genes. **a.** Digest of pSET152-ermE*-*ikaABC*-P2-ikaD with PciI/StuI. The second promoters (P2) were gapdhP(EL), kasOP*, P-2, P-15, P-31, rpsLP(XC), and SF14P (from left to right). All plasmids showed the predicted restriction pattern. **b.** Digest of pSET152-ermE*-*ikaABC*-P2-*ikaD* with EcoRI/StuI. The second promoters (P2) were gapdhP(EL), kasOP*, P-2, P-15, P-31, rpsLP(XC), and SF14P (from left to right). All plasmids showed the predicted restriction pattern.

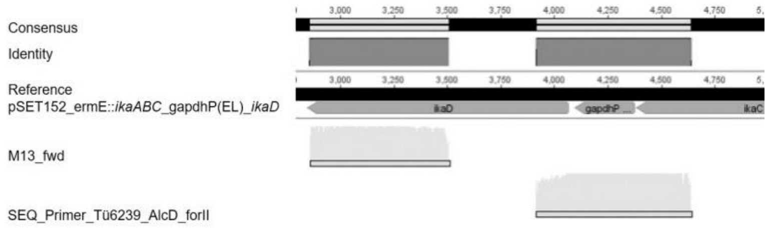

Figure S64. Sequencing result pSET152-ermE*-*ikaABC*-gapdhP(EL)-*ikaD*.

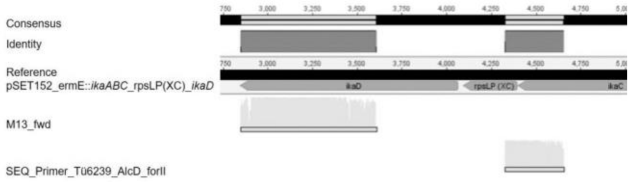

Figure S65. Sequencing result pSET152-ermE*-*ikaABC*-rpsLP(XC)-*ikaD*.

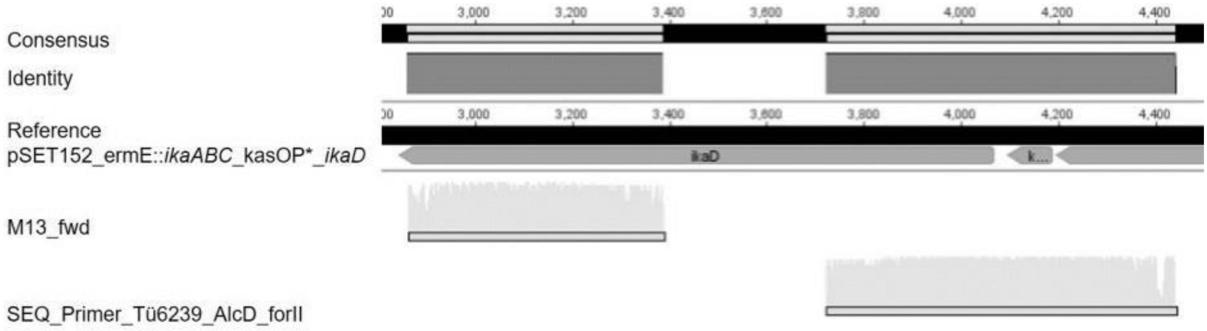

Figure S66. Sequencing result pSET152-ermE*-*ikaABC*-kasOP*-*ikaD*.

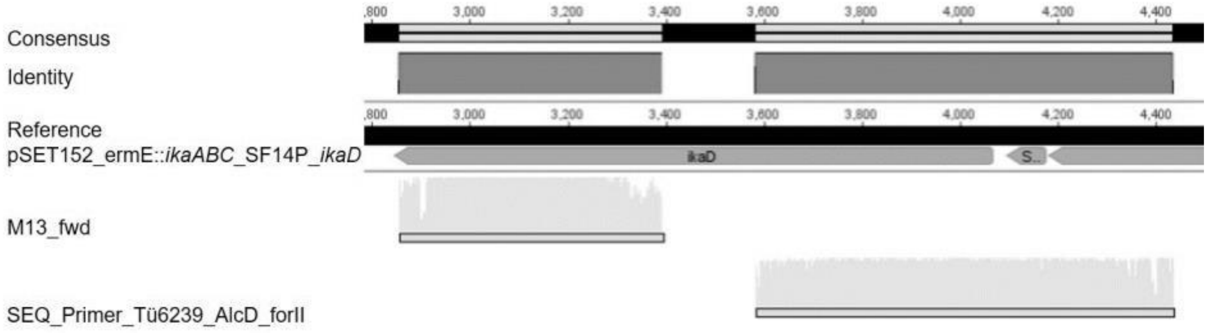

Figure S67. Sequencing result pSET152-ermE*-*ikaABC*-SF14P-*ikaD*.

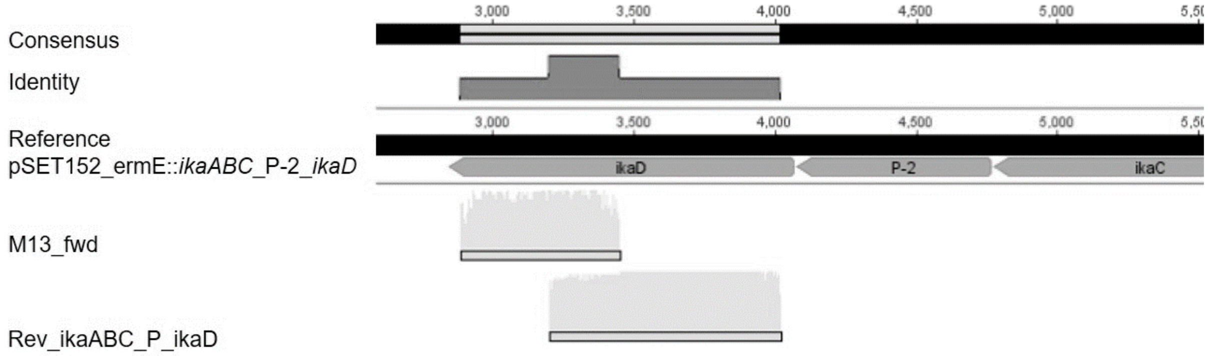

Figure S68. Sequencing result pSET152-ermE*-*ikaABC*-P-2-*ikaD*.

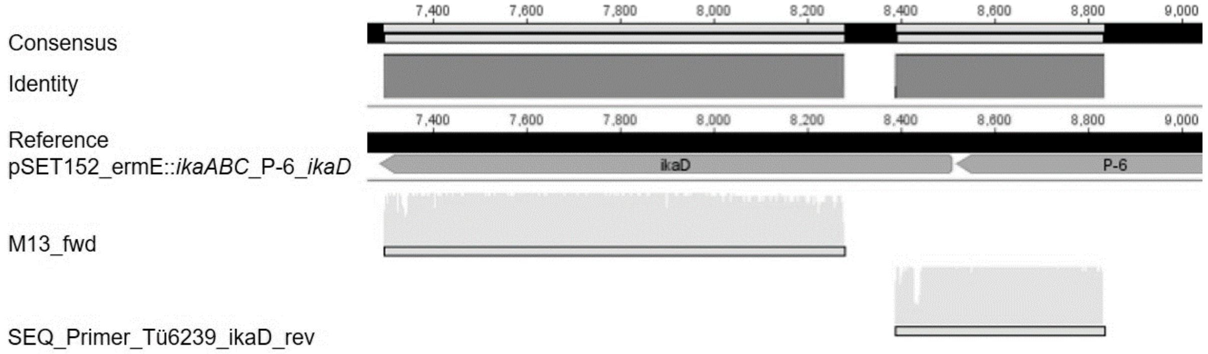

Figure S69. Sequencing result pSET152-ermE*-*ikaABC*-P-6-*ikaD*.

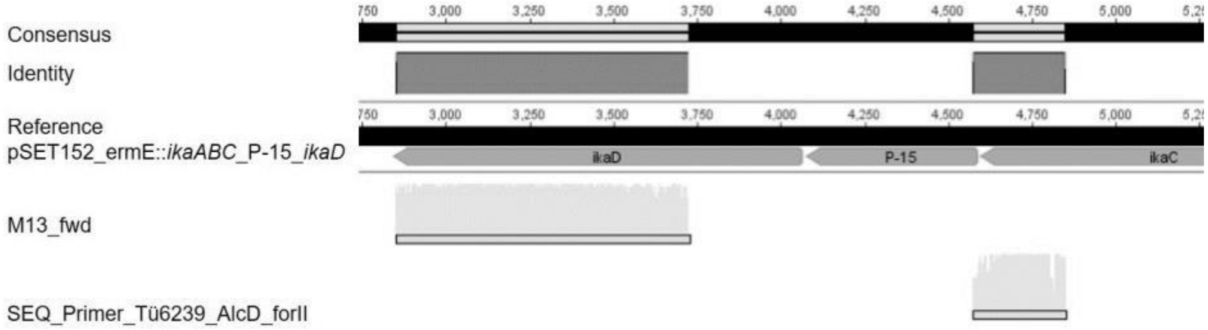

Figure S70. Sequencing result pSET152-ermE*-*ikaABC*-P-15-*ikaD*.

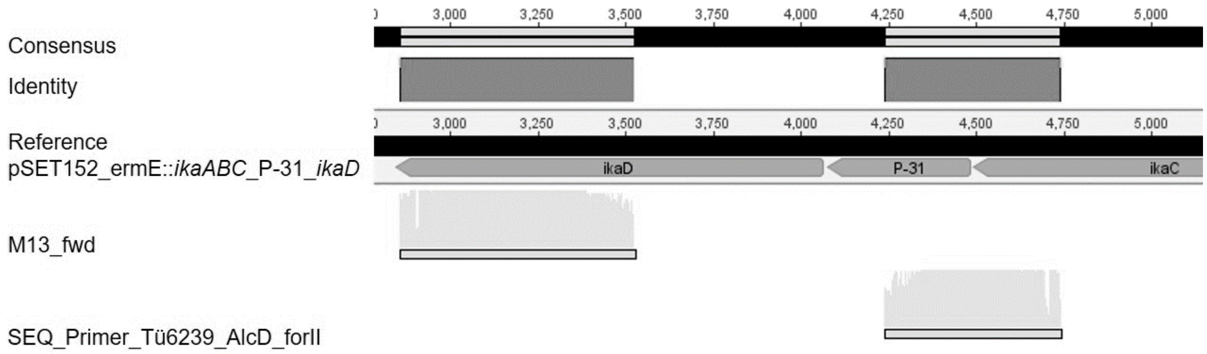

Figure S71. Sequencing result pSET152-ermE*-*ikaABC*-P-31-*ikaD*.

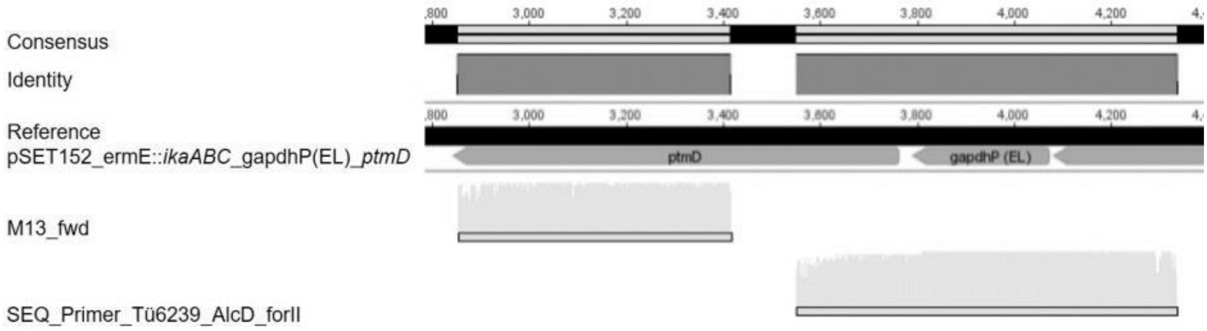

Figure S72. Sequencing result pSET152-ermE*-*ikaABC*-gapdhP(EL)-*ptmD*.

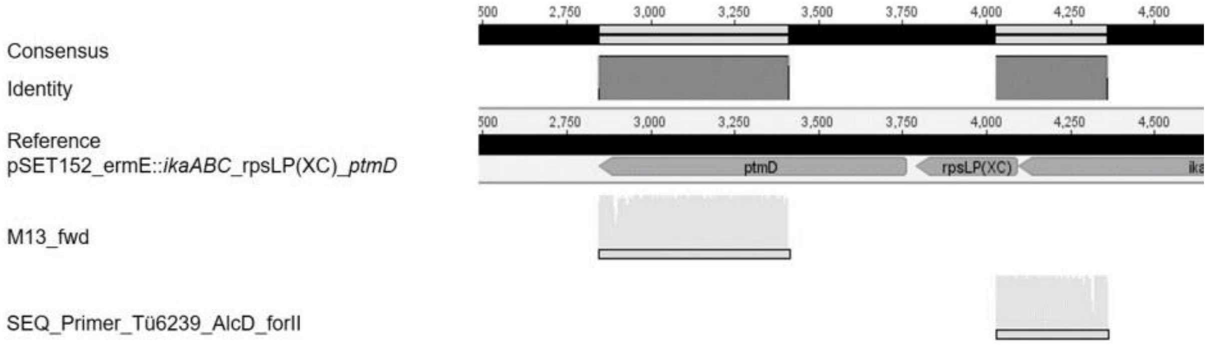

Figure S73. Sequencing result pSET152-ermE*-*ikaABC*-rpsLP(XC)-*ptmD*.

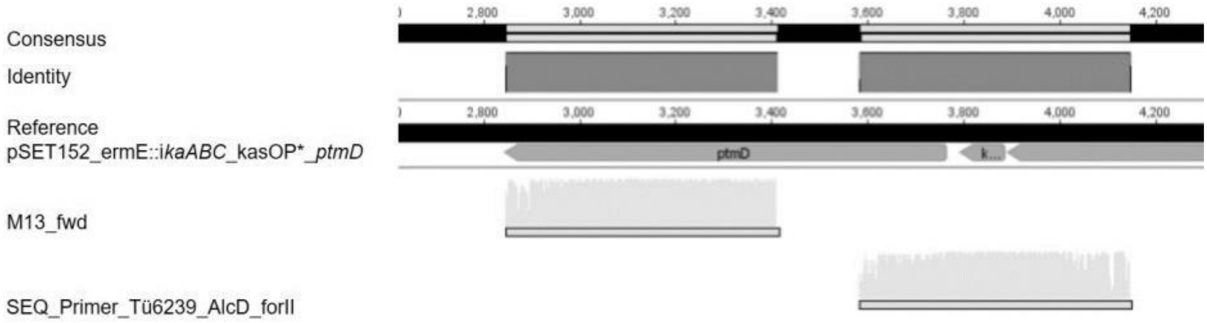

Figure S74. Sequencing result pSET152-ermE*-*ikaABC*-kasOP*-*ptmD*.

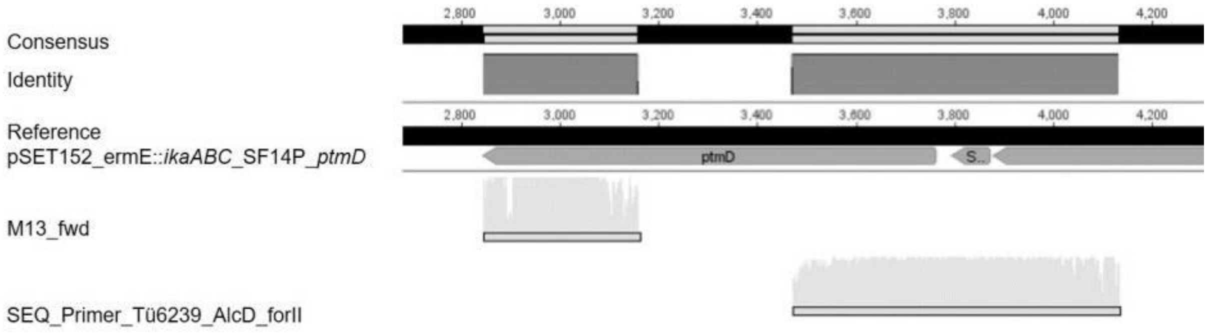

Figure S75. Sequencing result pSET152-ermE*-*ikaABC*-SF14P-*ptmD*

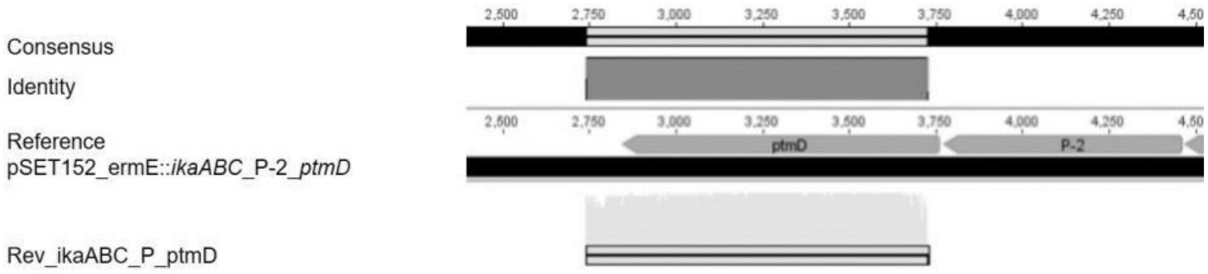

Figure S76. Sequencing result pSET152-ermE*-*ikaABC*-P-2-*ptmD.*

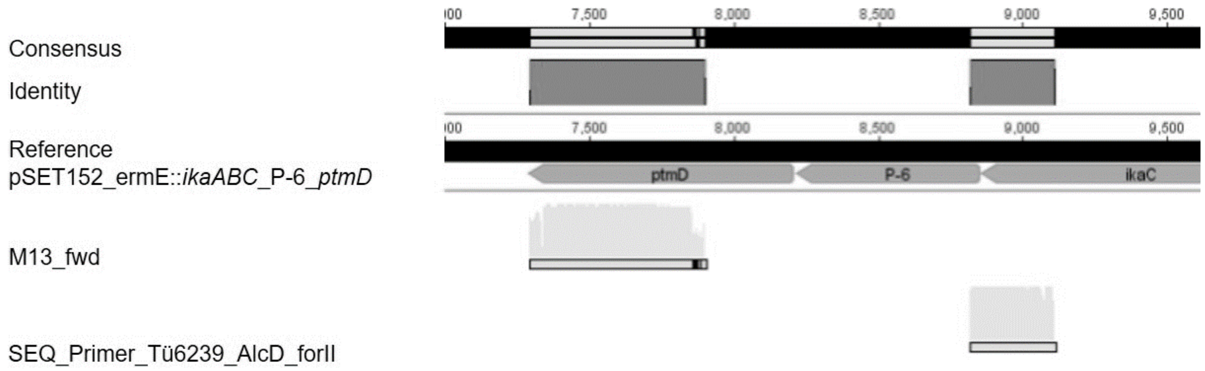

Figure S77. Sequencing result pSET152-ermE*-*ikaABC*-P-6-*ptmD.*

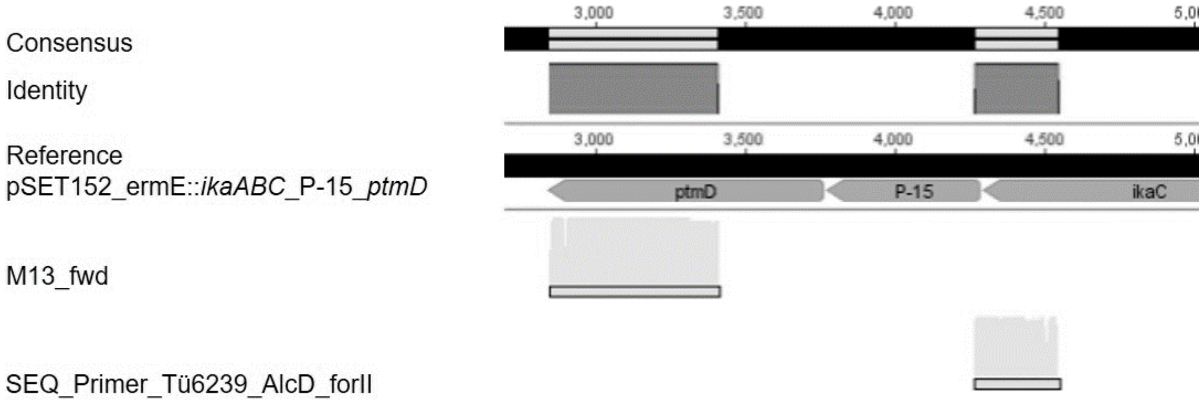

Figure S78. Sequencing result pSET152-ermE*-*ikaABC*-P-15-*ptmD*.

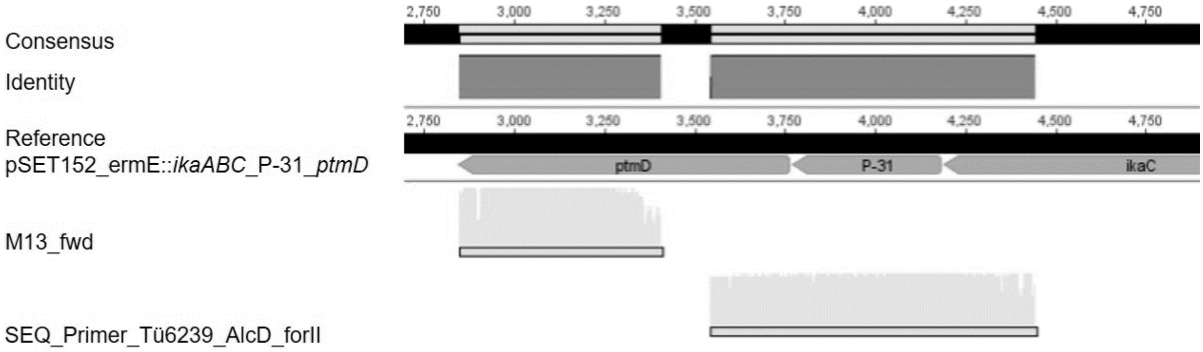

Figure S79. Sequencing result pSET152-ermE*-*ikaABC*-P-31-*ptmD*.

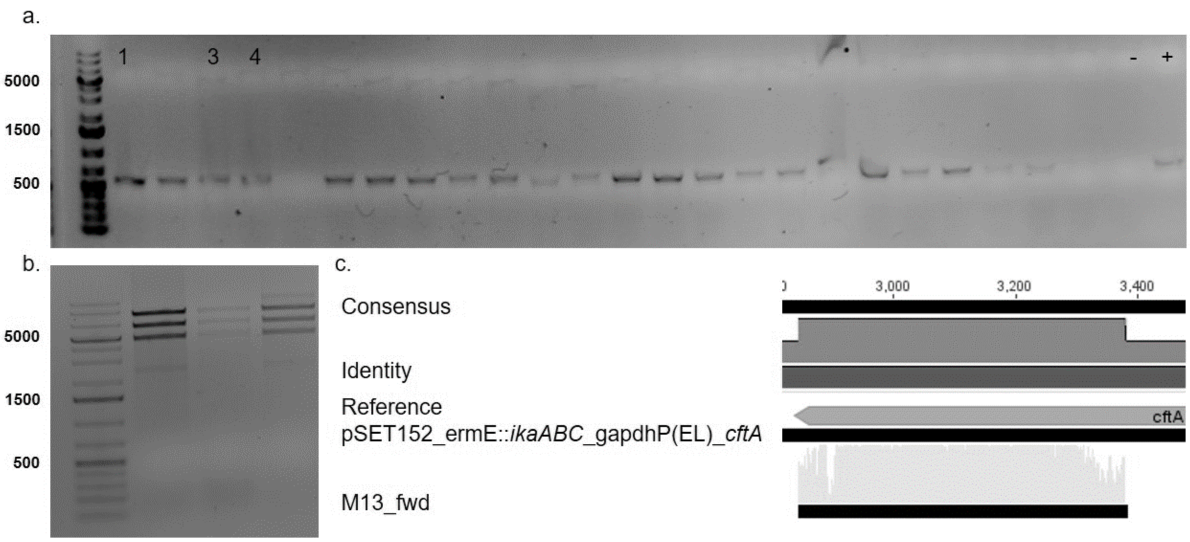

Figure S80. Verification of the cloning of pSET152-ermE*-*ikaABC*-gapdhP(EL)-*cftA*. **a** Colony screening PCR with expected bands for clones 1, 3, and 4 (480 bp). **b.** Analytical restriction digest (NcoI) with all clones showing the predicted restriction pattern. **c.** Sanger sequencing of clone 1 revealed 100% identity with the reference sequence.

8. Literature

1. Zhang W, Zhang G, Zhang L, Liu W, Jiang X, Jin H, et al. New polycyclic tetramate macrolactams from marine-derived *Streptomyces* sp. SCSIO 40060. Tetrahedron. 2018;74:6839–45.
2. Jakobi M, Winkelmann G, Kaiser D, Kempler C, Jung G, Berg G, et al. Maltophilin: a new antifungal compound produced by *Stenotrophomonas maltophilia* R3089. J Antibiot. 1996;49:1101–4.
3. Zhu W, Mei X, Wang Y, Liu P; Ocean Univ China. 5,5,6-polycyclic tetramic-acid containing macrocyclic lactam compound and preparation method and application thereof, 2018, CN108623607B.
4. Hou L, Liu Z, Yu D, Li H, Ju J, Li W. Targeted isolation of new polycyclic tetramate macrolactams from the deepsea-derived *Streptomyces somaliensis* SCSIO ZH66. Bioorg Chem. 2020;101:103954.
5. Jin H, Zhang W, Zhang G, Zhang L, Liu W, Zhang C. Engineered Biosynthesis of 5/5/6 Type Polycyclic Tetramate Macrolactams in an Ikarugamycin (5/6/5 Type)-Producing Chassis. Org Lett. 2020;22:1731–5.
6. Hoshino S, Wong CP, Ozeki M, Zhang H, Hayashi F, Awakawa T, et al. Umezawamides, new bioactive polycyclic tetramate macrolactams isolated from a combined-culture of *Umezawaea* sp. and mycolic acid-containing bacterium. J Antibiot. 2018;71:653–7.
7. Liu W, Zhang W, Jin H, Zhang Q, Chen Y, Jiang X, et al. Genome Mining of Marine-Derived *Streptomyces* sp. SCSIO 40010 Leads to Cytotoxic New Polycyclic Tetramate Macrolactams. Mar Drugs. 2019;17:663.
8. Jiao Y-J, Liu Y, Wang H-X, Zhu D-Y, Shen Y-M, Li Y-Y. Expression of the Clifednamide Biosynthetic Pathway in *Streptomyces* Generates 27,28-*seco*-Derivatives. J Nat Prod. 2020;83:2803–8.
9. Shigeno S, Kadowaki M, Nagai K, Hosoda K, Terahara T, Nishimura T, et al. New polycyclic tetramate macrolactams with antimycobacterial activity produced by marine-derived *Streptomyces* sp. KKMA-0239. J Antibiot. 2024;77:265–71.
10. MacNeil DJ, Gewain KM, Ruby CL, Dezeny G, Gibbons PH, MacNeil T. Analysis of *Streptomyces avermitilis* genes required for avermectin biosynthesis utilizing a novel integration vector. Gene. 1992;111:61–8.
11. Labeda DP, Doroghazi JR, Ju K-S, Metcalf WW. Taxonomic evaluation of *Streptomyces albus* and related species using multilocus sequence analysis and proposals to emend the description of *Streptomyces albus* and describe *Streptomyces pathocidini* sp. nov. Int J Syst Evol Microbiol. 2014;64:894–900.
12. Hopwood DA, Kieser T, Wright HM, Bibb MJ. Plasmids, Recombination and Chromosome Mapping in *Streptomyces lividans* 66. J Gen Microbiol. 1983;129:2257–69.
13. Kieser T, Hopwood DA, Wright HM, Thompson CJ. pIJ101, a multi-copy broad host-range *Streptomyces* plasmid: Functional analysis and development of DNA cloning vectors. Mol Gen Genet. 1982;185:223–38.
14. Wang G, Hosaka T, Ochi K. Dramatic Activation of Antibiotic Production in *Streptomyces coelicolor* by Cumulative Drug Resistance Mutations. Appl Environ Microbiol. 2008;74:2834–40.
